# Pre‐Planarized Triphenylamine‐Based Linear Mixed‐Valence Charge‐Transfer Systems

**DOI:** 10.1002/anie.202014567

**Published:** 2021-02-03

**Authors:** Marcel Krug, Nina Fröhlich, Dominik Fehn, Alexander Vogel, Frank Rominger, Karsten Meyer, Timothy Clark, Milan Kivala, Dirk M. Guldi

**Affiliations:** ^1^ Department of Chemistry and Pharmacy Interdisciplinary Center for Molecular Materials (ICMM) Friedrich-Alexander-Universität Erlangen-Nürnberg Egerlandstrasse 3 91058 Erlangen Germany; ^2^ Department of Chemistry and Pharmacy Chair of Organic Chemistry I Friedrich-Alexander-Universität Erlangen-Nürnberg Nikolaus-Fiebiger-Strasse 10 91058 Erlangen Germany; ^3^ Department of Chemistry and Pharmacy Inorganic Chemistry Friedrich-Alexander-Universität Erlangen-Nürnberg Egerlandstrasse 1 91058 Erlangen Germany; ^4^ Institute of Organic Chemistry Ruprecht-Karls-Universität Heidelberg Im Neuenheimer Feld 270 69120 Heidelberg Germany; ^5^ Centre for Advanced Materials Ruprecht-Karls-Universität Heidelberg Im Neuenheimer Feld 225 69120 Heidelberg Germany; ^6^ Department of Chemistry and Pharmacy, Computer-Chemistry-Center Friedrich-Alexander-University Erlangen-Nürnberg Naegelsbachstrasse 5 91052 Erlangen Germany

**Keywords:** electron transfer, mixed-valence compounds, *N*-heterotriangulenes, oxidation, radical cations

## Abstract

Three linear dimers with two redox‐active planarized triphenylamines were synthesized and their structures verified by X‐ray crystallography. Their radical cations, which exhibit electron self‐exchange between the two redox centers, are of great interest. This process was thoroughly investigated by means of electron paramagnetic resonance spectroscopy, absorption spectroscopy, and (time‐dependent) density functional theory calculations. A comparison of the key parameters of electron transfer with non‐planarized nitrogen‐centered building blocks emphasizes the impact of using redox centers with low internal reorganization energies. However, the distance‐dependence attenuation factor of the super‐exchange mechanisms remains similar.

## Graphical abstract

Novel building blocks for mixed‐valence charge‐transfer compounds: Planar *N*‐heterotriangulene dimers with low reorganization energies and activation barriers for electron self‐exchange have been synthesized and characterized.

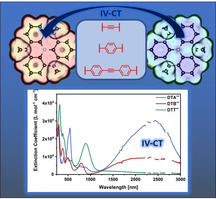

## Introduction

Electron‐transfer processes are to a large extent governed by two fundamental parameters; the electronic coupling *V* between electron donor and acceptor and the reorganization energy *λ*, which is composed of inner‐ and outer‐shell contributions.[^1^, ^2^] Mixed‐valence charge‐transfer (MV‐CT) systems, which contain at least two chemically equivalent entities with, however, different charges have been developed and probed in order to comprehend the interplay between the factors that control electron transfer.[^3^, ^4^]

Robin and Day proposed a classification system for MV‐CT systems based on the electronic interaction strength between the redox‐active centers.^[5]^ Class I is the extreme case of diabaticity in which negligible electronic communication leaves the redox centers isolated and independent from each other. Class II systems exhibit sizeable electronic communication between the redox entities. This is manifested in, for example, a splitting of the redox potentials. It is notable that the charge is located on one of the two specific redox entities, but can be transferred by a thermally or optically induced electron transfer. A double‐well shaped potential‐energy surface along the electron‐transfer coordinate is typical for Class II compounds. In Class III, extraordinarily strong coupling forces the charge to be delocalized across the entire system. Class III compounds are also referred to as “charge resonance systems” and exhibit a single‐minimum potential‐energy surface along the electron‐transfer coordinate.

In weakly‐coupled Class II MV‐CT systems, optically induced intervalence charge transfer (IV‐CT) manifests itself in a strong near‐infrared (nIR) absorption. According to Mulliken–Hush theory,[^3^, ^4^, ^6^, ^7^] reorganization energy and electronic coupling are derived by analyzing the shape, intensity, and energetic position of the IV‐CT bands. In cases of a degenerate electron transfer between chemically identical redox sites, the reorganization energy is equal to the absorption maximum $\tilde{\nu }$_max_ [Eq. 1].

(1) $${\lambda =\tilde{\nu }}_{max}$$

The electronic coupling is proportional to the intensity of the IV‐CT band [Eqs. 2–5].

(2) $$V=\;\frac{\mu_{eg}{\tilde{\nu }}_{max}}{\Delta \mu_{12}}$$

(3) $$\mu_{eg}=0.09584\;\sqrt{\frac{\int \epsilon \left(\tilde{\nu }\right)d\tilde{\nu }}{{\tilde{\nu }}_{max}}}$$

(4) $$\Delta \mu_{12}=\sqrt{\Delta \mu_{eg}^{2}+4\mu_{eg}^{2}}$$

(5) $$\Delta \mu_{eg}=e\times r$$

In these equations, *μ*_eg_ is the transition dipole moment, which is calculated by integrating the IV‐CT band. Δ*μ*_12_ is the diabatic dipole moment difference, which is derived from *μ*_eg_ and Δ*μ*_eg_, the adiabatic dipole moment difference. The latter is approximated as the distance *r* between the redox centers multiplied with the elementary charge *e*.

Molecular MV‐CT systems feature two oxidizable or reducible building blocks covalently linked through molecular bridges. The molecular bridges serve two roles: as an electronic coupling medium and as a distance keeper. They are typically built from carbon frameworks, such as aromatics,[^8^, ^9^, ^10^, ^11^] alkynes,[^8^, ^12^] cyclophanes,[^13^, ^14^, ^15^] oligo‐*p*‐phenylene vinylenes,[^16^, ^17^] etc., or combinations thereof.[^18^, ^19^] All of these allow the electronic coupling to be fine‐tuned at either short or long distances.

**Figure 1:**
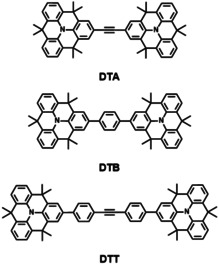
Chemical structures of the *N*‐heterotriangulene dimers DTA, DTB, and DTT.

Triarylamines are among the most studied redox sites for MV‐CT systems.[^3^, ^4^] Important features are a reversible oxidation at low potentials and a reasonable stability of the respective radical cations. In triphenylamine MV‐CT systems, IV‐CT bands are typically well separated from local transitions, which simplifies the band‐shape analysis. Propeller‐shaped triarylamines tend, however, to undergo planarization upon oxidation, leading to large inner reorganization energies. To address this aspect, we have designed three different dimers of pre‐planarized triarylamines (*N*‐heterotriangulenes (*N*‐HTAs))[^20^, ^21^, ^22^, ^23^] as redox centers and acetylene (DTA), *p*‐phenylene (DTB), and tolane (DTT) as molecular bridges (Figure 1).

Fang et al. have pointed out that the oxidation potential in CH_2_Cl_2_ of unsubstituted *N*‐HTA (+0.34 V vs. Fc/Fc^+^) is less positive than that of triphenylamine (+0.54 V vs. Fc/Fc^+^).^[24]^ The radical cation of *N*‐HTA generated upon oxidation is also found to be slightly more persistent than that of triphenylamine.[^24^, ^25^] To evaluate the impact of the planarization on the reorganization energy and the electronic coupling, we investigated the dimers and their radical cations by means of electrochemical methods, absorption spectroscopy, and electron paramagnetic resonance spectroscopy (EPR). IV‐CT parameters were obtained by absorption‐band analysis in the context of the Mulliken–Hush theory. The experimental findings were further clarified by calculations using the BLYP35 density functional, which mixes 35 % Hartree–Fock (HF) exact exchange into the BLYP exchange correlation functional. This functional has been shown to provide good results for problems in which the localization/delocalization of charge centers is important.[^26^, ^27^]

## Results and Discussion

The differently linked dimers, that is, DTA, DTB, and DTT, were synthesized from known *N*‐HTA building blocks and the molecular bridges through transition‐metal‐catalyzed cross‐coupling reactions. The dimers are colorless to pale yellow solids, which are environmentally stable and soluble in common organic solvents. The synthetic details and characterization data are given in the Supporting Information (SI) Section 3.

**Figure 2:**
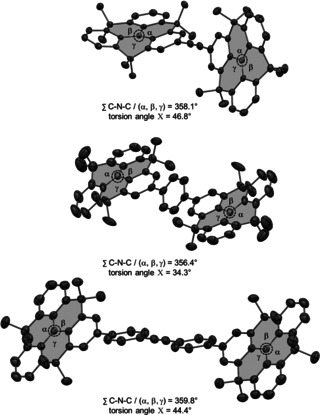
ORTEP diagram of DTA (top), DTB (middle), and DTT (bottom) (thermal ellipsoids at 50 % probability, hydrogens are omitted for clarity).^[36]^ Overview of present torsion angles *Χ* and ∑C‐N‐C/(α, β, γ) angles.

Single crystals of DTA and DTT suitable for X‐ray crystallography were obtained from slow liquid diffusion of MeOH into CH_2_Cl_2_ solutions of the compounds at room temperature, while single crystals of DTB were obtained from slow evaporation of CD_2_Cl_2_ (Figure 2). Although a coplanar orientation of the two *N*‐HTAs is expected due to the acetylene bridge, DTA shows a rather distorted structure in which the two *N*‐HTAs are twisted along the linear C(sp) bridge, with a torsion angle *Χ* of 46.8°. Nevertheless, both *N*‐HTAs show a characteristic planarization around the nitrogen center with a sum of the C‐N‐C/(α, β, γ) angles of 358.1°. For DTA, no specific packing motif is observed in the solid state (for details regarding the crystal packing, see the SI Section 5). In contrast, the *N*‐HTAs connected through the benzene bridge in DTB are arranged in a coplanar fashion due to the crystallographic inversion symmetry. Moreover, a torsion angle *Χ* of 34.3° is found between the *N*‐HTAs and the phenylene bridge. Again, the nitrogen centers of the *N*‐HTAs in DTB are almost planar. Analogously to DTB, the *N*‐HTAs in DTT are connected similarly through the tolane bridge in a coplanar fashion. Again, due to an inversion symmetry center, we note a lack of “global torsion”. A torsion angle *Χ* of 44.4° is observed between the *N*‐HTAs and the adjacent phenyl rings of the tolane bridge. Furthermore, due to symmetry reasons, no torsion is found between the phenyl groups adjacent to the acetylene moiety, which is exactly the same feature as in the unsubstituted tolane.^[28]^ A characteristic planarization around the nitrogen centers of the *N*‐HTAs with a sum of the C‐N‐C/(α, β, γ) angles of 359.8° is found for DTT, consistent with DTA and DTB. Overall, the arrangement is most likely enforced by crystal‐packing forces.

The peak separations in the Square Wave voltammograms (SWV, Figure S22) allow a qualitative assessment of the electronic interactions between the *N*‐HTAs. In CH_2_Cl_2_ and in the presence of 0.2 m TBAPF_6_, two well‐separated oxidations are noted at +0.37 and +0.54 V vs. Fc/Fc in the SWV of DTA. The peak separations for the oxidations of DTB are far lower with +0.38 and +0.48 V vs. Fc/Fc^+^. In DTT, which features a tolane linker, the peak separation is as small as 0.01 V based on oxidations at +0.39 and +0.40 V vs. Fc/Fc^+^. Table 1 summarizes the electrochemical data together with the estimated comproportionation constants calculated from the peak separation. At this point, we conclude that the electronic communication is the strongest in DTA, followed by DTB and DTT.

**Table 1:** First and second oxidations in V vs. Fc/Fc^+^ determined by Square Wave voltammetry for DTA, DTB, and DTT and the comproportionation constants derived from the peak separation.

Compound	*E*_ox1_	*E*_ox2_	Δ*E* [V]	*K*_CO_
DTA	+0.37	+0.54	0.17	840
DTB	+0.38	+0.48	0.10	53
DTT	+0.39	+0.40	0.01	1.5

Also, the electronic coupling is evaluated from orbital interaction diagrams (see SI Section 8.2.3). The energy differences between HOMO and HOMO−1 allow a coarse estimate of the electronic coupling, which itself is a consequence of orbital interactions between the bridges and the electron donors.[^29^, ^30^] For DTA, we calculate an energetic separation of 0.42 eV. This energy difference decreases to 0.20 and 0.12 eV for DTB and DTT, respectively, matching the trend found in the electrochemical investigations.

**Figure 3:**
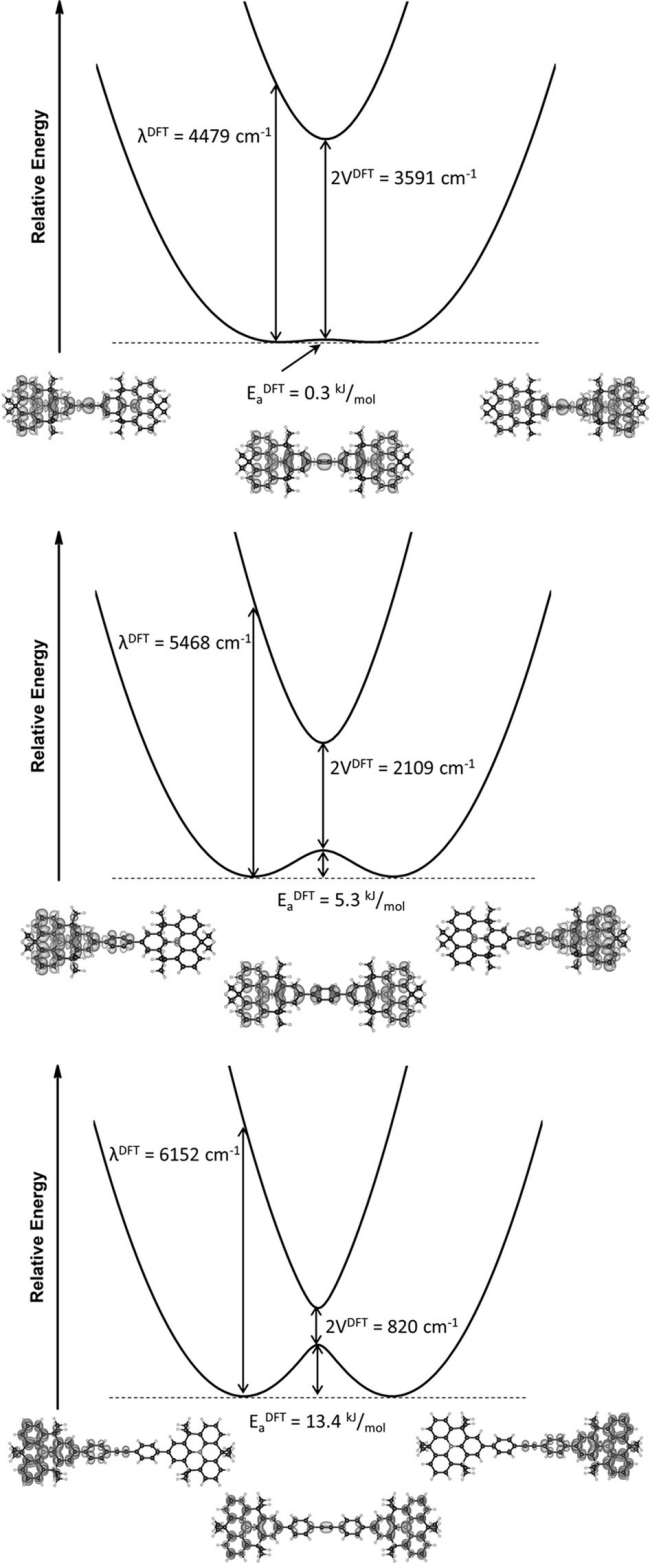
Energy diagrams of IV‐CT within DTA^.+^ (top), DTB^.+^ (middle), and DTT^.+^ (bottom) estimated by TD‐BLYP35 calculations in CH_2_Cl_2_. The unsymmetric spin densities correspond to the localized charge distribution and the symmetric ones to the transition state (isosurface: ±0.001 a.u.).

Density functional theory (DFT) allows us to estimate the electronic coupling and reorganization energy of the dimers (Figure 3).[^29^, ^31^] For this approach, unsymmetric and symmetric structures were calculated. These correspond to the localized electronic structures and the transition state of thermal electron transfer, respectively.

In Mulliken–Hush theory, the reorganization energy is easily obtained through time‐dependent DFT (TDDFT) calculations as the excitation energy of the lowest energetic transition of the unsymmetric structure. The charge‐transfer nature of this transition was confirmed through natural transition orbital (NTO) analyses (SI Section 8.3). In other words, the initial NTO is located on the neutral *N*‐HTA and the final NTO on the oxidized one. Reorganization energies of 4479, 5468, and 6152 cm^−1^ were found for DTA^.+^, DTB^.+^, and DTT^.+^, respectively.

The activation barriers for electron transfer, calculated as the energy difference between the unsymmetric and the symmetric structures, are found to be 0.3, 5.3, and 13.4 kJ mol^−1^ for DTA^.+^, DTB^.+^, and DTT^.+^, respectively.

Finally, the electronic coupling was derived from the excitation energy of the lowest vertical transition of the symmetric transition‐state structure. According to Mulliken–Hush theory, this energy corresponds to twice the electronic coupling and yields 1796, 1054, and 410 cm^−1^ for DTA^.+^, DTB^.+^, and DTT^.+^, respectively.

The fact that the localized structures are the most stable minima suggests that the *N*‐HTA dimers belong to the Robin–Day Class II. DTA^.+^ is, however, an exception. Although the calculated spin density is largely concentrated on one *N*‐HTA, some of it is also seen on the other. This suggests that the positive charge is delocalized to some extent and, in turn, communicates strongly via the acetylene bridge.

In addition, localization/delocalization of the positive charges within the radical cations were investigated with EPR spectroscopy. Temperature‐dependent CW X‐band EPR spectroscopy was conducted after in situ oxidation of 1 mm solutions of the dimers in CH_2_Cl_2_ with SbCl_5_. In all cases, isotropic signals were detected at 95 K with effective *g*‐values centered at *g* ${}_{\mathrm{iso},\mathrm{DTA}•{}^{+}}$ =2.0016, *g* ${}_{\mathrm{iso},\mathrm{DTB}•{}^{+}}$ =2.0043, and *g* ${}_{\mathrm{iso},\mathrm{DTT}•{}^{+}}$ =2.0015 with linewidths of *W* ${}_{\mathrm{iso},\mathrm{DTA}•{}^{+}}$ =0.720×10^−4^ cm^−1^/GHz, *W* ${}_{\mathrm{iso},\mathrm{DTB}•{}^{+}}$ =0.774×10^−4^ cm^−1^/GHz, and *W* ${}_{\mathrm{iso},\mathrm{DTT}•{}^{+}}$ =0.290×10^−4^ cm^−1^/GHz.

**Figure 4:**
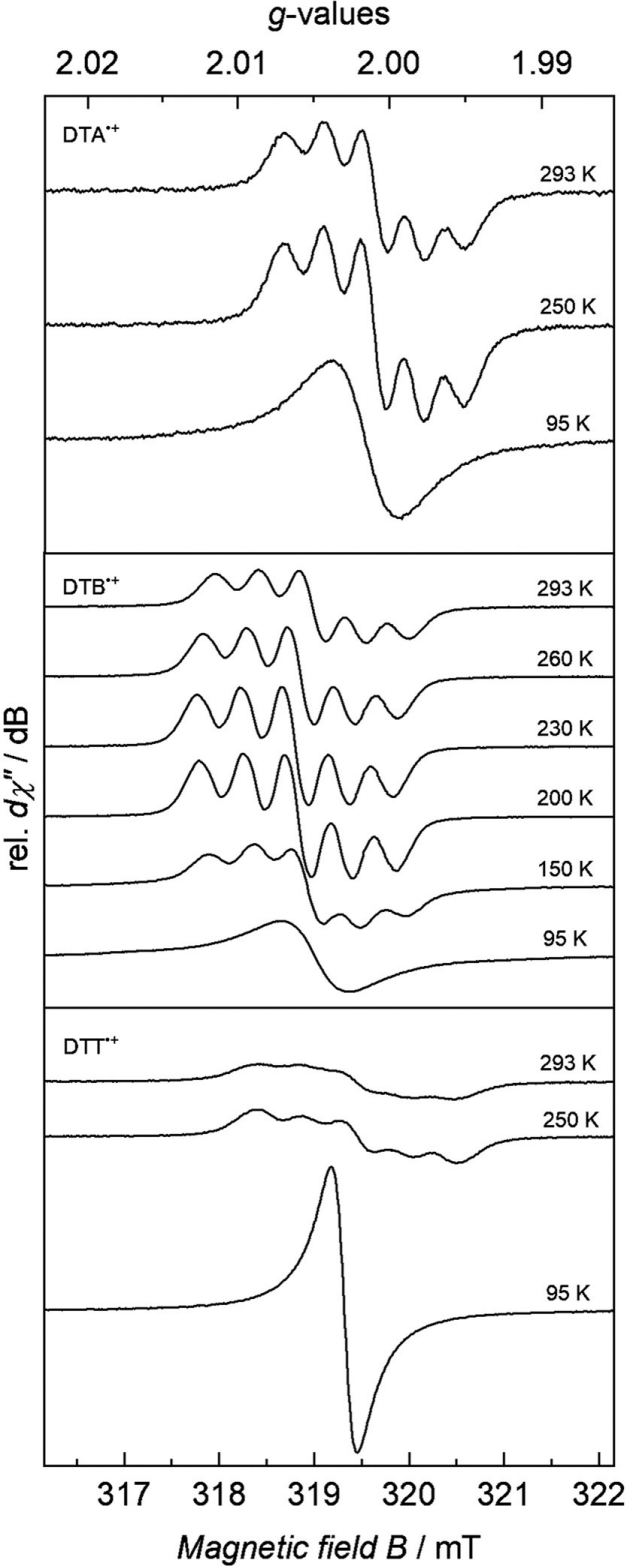
Experimental CW X‐band EPR spectra of DTA^.+^, DTB^.+^, and DTT^.+^ as 1 mm solutions in DCM at different temperatures. Experimental conditions: microwave frequency *ν*=8.959 GHz, modulation width=0.1; 0.01 mT, microwave power=1.00 mW, modulation frequency=100 kHz, time constant=0.1 s. For data simulation, see Supporting Information.

At low temperatures, the linewidth is larger than the hyperfine interaction. This increase in linewidth prevents the observation of a presumed three‐line pattern at low temperatures. The increase in linewidth is attributed to a large number of different mechanisms that affect the line broadening.^[32]^ With increasing temperature, a five‐line pattern is observed (Figure 4), which implies superhyperfine interaction of the unpaired electrons with two equivalent ^14^N (^14^N, *I*=1, 99.6 % nat. abundance) nuclei. The superhyperfine coupling constants were determined to be *A* ${}_{\mathrm{iso},{}^{14}N,\mathrm{DTA}•{}^{+}}$ =3.73×10^−4^ cm^−1^, *A* ${}_{\mathrm{iso},{}^{14}N,\mathrm{DTB}•{}^{+}}$ =3.99×10^−4^ cm^−1^, and *A* ${}_{\mathrm{iso},{}^{14}N,\mathrm{DTT}•{}^{+}}$ =4.10×10^−4^ cm^−1^ at RT. Increasing the temperature from 95 to 293 K leads directly to the formation of a fully delocalized five‐line pattern, which implies that the spin‐exchange process occurs on timescales faster than the timescale of the EPR measurements.

From the DFT calculations, DTA^.+^ appears to be at the border between Robin–Day Class II and III. For an unambiguous assignment, we performed electrochemical oxidation of DTA and monitored the changes in the IR region (SI Figure S30). Besides the emergence of a strong absorption tail below 3500 nm, a narrow and much less intense absorption is seen at 4671 nm (2141 cm^−1^). This corresponds to the stretch vibration of the central triple bond. Its presence corroborates an unsymmetric electronic structure for the monocation. Due to symmetry restrictions, neutral DTA and its dication, with their symmetric electronic structures, lack this feature. This finding places DTA^.+^ unambiguously into Robin–Day Class II and allows a treatment through Mulliken–Hush analyses. Similarly, a less intense vibrational absorption is found for DTT^.+^ at 4523 nm (2211 cm^−1^), but not for DTT or DTT^2+^ (Figure S34). In contrast, DTB^.+^, which has no C−C triple bonds, does not show vibrational features in this region (Figure S32). All cations exhibit absorptions in the range from 5960 to 6300 nm; these stem from aromatic ring vibrations of the *N*‐HTAs. The assignment has been verified by DFT calculations of the normal vibrations.

**Figure 5:**
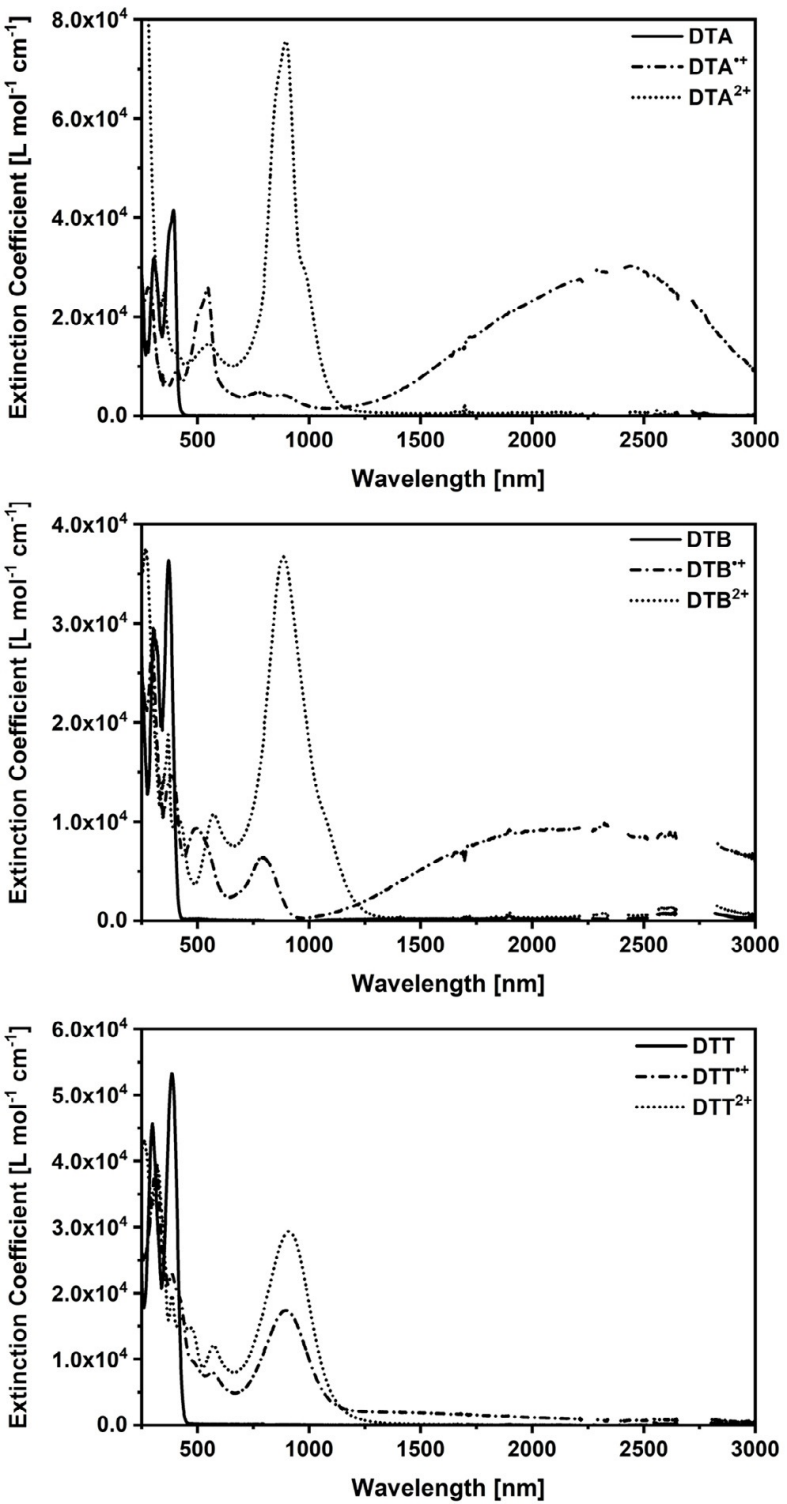
Absorption spectra of neutral (solid lines), cationic (dash‐dotted lines), and dicationic (dotted lines) DTA (top), DTB (middle), and DTT (bottom) in CH_2_Cl_2_. The charged species were generated in situ by addition of SbCl_5_.

An experimental evaluation of the electron‐transfer process is achieved by means of Mulliken–Hush analyses of the nIR absorption bands of the monocations. SbCl_5_ was used to oxidize DTA, DTB, and DTT in CH_2_Cl_2_ step by step to the corresponding mono‐ and dications and the process was monitored by absorption spectroscopy. The collected absorption spectra of the generated species are shown in Figure 5. A rather broad nIR absorption is common to the spectra of DTA^.+^, DTB^.+^, and DTT^.+^. All spectra are in sound agreement with our calculated spectra (SI Section 8.3). Thus, based on the NTO analyses, the lowest‐energy transition in the nIR is assigned to the IV‐CT and in the higher energy visible regime, local excitations take place on the oxidized and/or neutral *N*‐HTA.

The experimental nIR maxima determined from fits of the absorption spectra also provide the basis for the Mulliken–Hush analysis (SI Section 7.3). Asymmetric peak functions were, however, necessary to describe the nIR absorptions of DTA^.+^ and DTB^.+^ and afforded maxima at 2411 nm (4147 cm^−1^) and 2228 nm (4489 cm^−1^), respectively. Notably, the nIR absorption of DTT^.+^ is partially masked by local excitations. As such, we can fit a reasonable section of the absorption spectrum with Gaussian functions. In accordance with TDDFT calculations, the Gaussian function at the longest wavelength corresponds to the IV‐CT transition: 1399 nm (7148 cm^−1^).

**Figure 6:**
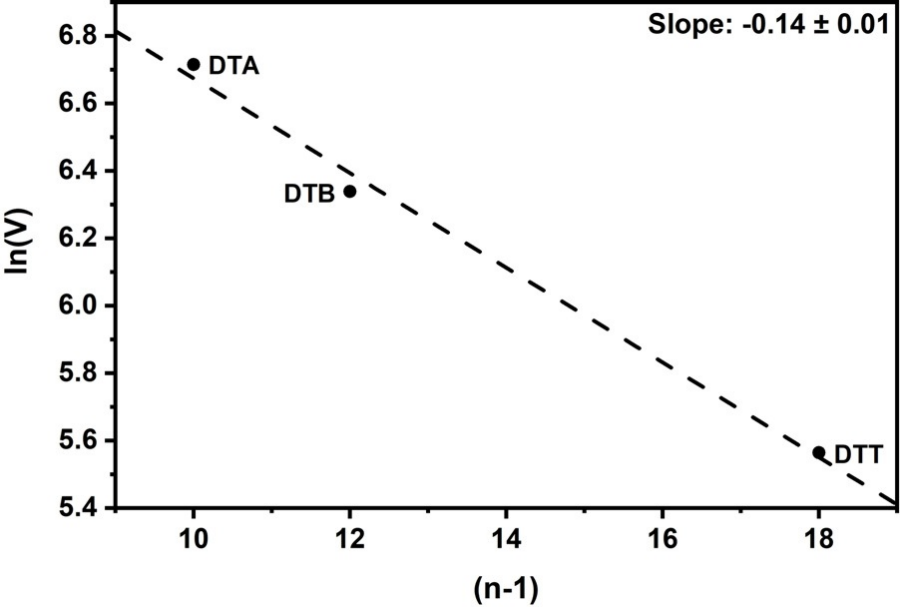
Linear correlation between ln(*V*) and the electron‐transfer distance represented by the parameter (*n*−1), whereby *n* is the number of bonds between the nitrogen atoms.

Using Equations (1) to (5), we calculated the reorganization energy and electronic coupling element for DTA^.+^, DTB^.+^, and DTT^.+^ (Table 2 and Table S5). The electronic coupling decreases from 825 cm^−1^ in DTA^.+^ to 566 cm^−1^ in DTB^.+^ and 261 cm^−1^ in DTT^.+^. It is highly relevant that we found a linear correlation between the electron‐transfer distance and ln(*V*) (Figure 6). This led us to propose a superexchange mechanism.^[34]^ The slope was calculated to be −0.14±0.01, fully consistent with structurally related systems containing arylamines (−0.16)^[34]^ and *N*,*N′*‐dihydrodimethylphenazine (DHP; −0.17)^[33]^.

**Table 2:** Characteristic electron‐transfer parameters obtained through Mulliken–Hush analysis of the nIR absorption bands and relevant EPR parameters obtained in CH_2_Cl_2_. For a comparison, experimental results for acetylene‐bridged triarylamine (R1) and *N*,*N′*‐dihydrodimethylphenazine (R2) from Lambert et al. (structures are depicted in the SI) are also included for comparison.^[33]^

	*R* [Å]	*V*_MH_ [cm^−1^]	*λ*_MH_ [cm^−1^]	Δ*G* ^ǂ^_MH_ [kJ mol^−1^]	*g‐*value at RT	Linewidth (FWHM) at RT [10^−4^ cm^−1^/GHz]	Hyperfine constant *A* at RT [10^−4^ cm^−1^]
DTA^.+^	12.5	825	4147	4.5	2.0015	0.385	3.73
DTB^.+^	14.3	566	4489	7.5	2.0044	0.400	3.99
DTT^.+^	21.2	261	7147	18.4	2.0015	0.550	4.10
R1^[33]^	12.5	980	6600	8.9	–	–	–
R2^[33]^	12.5	870	8500	10.9	–	–	–

The reorganization energy gives the opposite trend. Its lowest value (4147 cm^−1^) is found for DTA^.+^ and increases to 4489 and 7147 cm^−1^ for DTB^.+^ and DTT^.+^, respectively. One apparent rationale is the rotational flexibility, which increases as the molecular bridges become longer. Analyses of the Stokes shift of the neutral forms, which is a measure of the reorganization upon photoexcitation between the ground and excited state, further corroborates our interpretation (please compare SI Section 7.1).

Even though the electronic coupling in DTA^.+^ is slightly lower than in the acetylene‐bridged triarylamine and DHP dimers, the electron‐transfer reorganization energy is significantly reduced in direct comparison to these non‐planar electron donors. Notably, the DHPs feature the highest reorganization energy due to their butterfly‐shaped geometry.^[33]^ Overall, the pre‐planarization of our *N*‐HTAs reduces the reorganization energy by up to 35 % in comparison to triarylamines. The ratio between the electronic coupling and reorganization energy places DTA^.+^, DTB^.+^, and DTT^.+^ into Robin–Day Class II. An estimation of the electron‐transfer activation barrier is achieved by Equation 6.[^7^, ^8^, ^35^]

(6) $$\Delta G^{\neq }=\frac{{\left(\lambda -2V\right)}^{2}}{\begin{array}{c} 4\;\lambda \\ \; \end{array}}$$

Minimizing the reorganization energy for electron transfer goes hand in hand with electron‐transfer activation barriers as small as 4.5 kJ mol^−1^. Please note that the activation barrier for an arylamine derivative R1 investigated by Lambert and co‐workers is almost twice as large.^[33]^ This further underlines the beneficial effects of pre‐planarizing the electron‐transfer properties of *N*‐HTAs.

## Conclusion

In summary, three linear *N*‐HTA dimers, DTA, DTB, and DTT, with π‐conjugated molecular bridges of different lengths, were synthesized and their oxidations thoroughly investigated in the context of mixed‐valence charge transfer (MV‐CT). For their singly oxidized radical cations, localized structures were predicted by DFT using the BLYP35 functional, which indicated Robin–Day Class II behavior. Upon chemical oxidation, strong nIR absorptions were noted for DTA^.+^, DTB^.+^, and DTT^.+^. Band analyses within the framework of the Mulliken–Hush theory revealed that, on the one hand, the electronic coupling decreases with distance, while, on the other hand, the reorganization energy increases. All our systems belong to Class II of the Robin–Day classification. Considering the pre‐planarized structure of *N*‐HTA, the reorganization energy of the smallest dimer is significantly lower than for analogous systems featuring triarylamines or *N*,*N′*‐dihydrodimethylphenazines. At the end of the day, the activation barrier estimated through the Mulliken–Hush analyses for DTA^.+^ is approximately half of what is found for analogous triarylamine systems. These findings render planar *N*‐HTA a promising alternative to the propeller‐shaped triarylamines.

## Conflict of interest

The authors declare no conflict of interest.

## Supporting information

As a service to our authors and readers, this journal provides supporting information supplied by the authors. Such materials are peer reviewed and may be re‐organized for online delivery, but are not copy‐edited or typeset. Technical support issues arising from supporting information (other than missing files) should be addressed to the authors.

Supplementary

## References

[1] R. A. Marcus , Annu. Rev. Phys. Chem. 1964, 15, 155–196.

[2] R. A. Marcus , N. Sutin , Biochim. Biophys. Acta Rev. Bioenerg. 1985, 811, 265–322.

[3] A. Heckmann , C. Lambert , Angew. Chem. Int. Ed. 2012, 51, 326–392; 10.1002/anie.201100944 22095940

[4] J. Hankache , O. S. Wenger , Chem. Rev. 2011, 111, 5138–5178. 21574545 10.1021/cr100441k

[5] M. B. Robin , P. Day , Adv. Inorg. Chem. Radiochem. 1968, 10, 247–422.

[6] N. S. Hush , Electrochim. Acta 1968, 13, 1005–1023.

[7] B. S. Brunschwig , N. Sutin , Coord. Chem. Rev. 1999, 187, 233–254.

[8] S. V. Rosokha , D. L. Sun , J. K. Kochi , J. Phys. Chem. A 2002, 106, 2283–2292.

[9] C. Lambert , G. Nöll , J. Schelter , Nat. Mater. 2002, 1, 69–73. 12618853 10.1038/nmat706

[10] C. Lambert , C. Risko , V. Coropceanu , J. Schelter , S. Amthor , N. E. Gruhn , J. C. Durivage , J. L. Brédas , J. Am. Chem. Soc. 2005, 127, 8508–8516. 15941286 10.1021/ja0512172

[11] H. W. Jung , S. E. Yoon , P. J. Carroll , M. R. Gau , M. J. Therien , Y. K. Kang , J. Phys. Chem. B 2020, 124, 1033–1048. 31927963 10.1021/acs.jpcb.9b09578

[12] V. Coropceanu , M. Malagoli , J. M. André , J. L. Brédas , J. Am. Chem. Soc. 2002, 124, 10519–10530. 12197754 10.1021/ja026437j

[13] B. Mladenova , D. R. Kattnig , C. Kaiser , J. Schäfer , C. Lambert , G. Grampp , J. Phys. Chem. C 2015, 119, 8547–8553.

[14] D. R. Kattnig , B. Mladenova , G. Grampp , C. Kaiser , A. Heckmann , C. Lambert , J. Phys. Chem. C 2009, 113, 2983–2995.

[15] S. Amthor , C. Lambert , J. Phys. Chem. A 2006, 110, 1177–1189. 16420023 10.1021/jp0550309

[16] V. Lloveras , J. Vidal-Gancedo , T. M. Figueira-Duarte , J. F. Nierengarten , J. J. Novoa , F. Mota , N. Ventosa , C. Rovira , J. Veciana , J. Am. Chem. Soc. 2011, 133, 5818–5833. 21446654 10.1021/ja1083859

[17] S. Barlow , C. Risko , S. J. Chung , N. M. Tucker , V. Coropceanu , S. C. Jones , Z. Levi , J. L. Brédas , S. R. Marder , J. Am. Chem. Soc. 2005, 127, 16900–16911. 16316236 10.1021/ja054136e

[18] M. M. Hansmann , M. Melaimi , G. Bertrand , J. Am. Chem. Soc. 2018, 140, 2206–2213. 29342351 10.1021/jacs.7b11184

[19] A. Heckmann , S. Amthor , C. Lambert , Chem. Commun. 2006, 2959–2961. 10.1039/b604603g 16832503

[20] D. Hellwinkel , Chem. Ber. 1974, 107, 616–626.

[21] N. Hammer , T. A. Schaub , U. Meinhardt , M. Kivala , Chem. Rec. 2015, 15, 1119–1131. 26223442 10.1002/tcr.201500202

[22] T. A. Schaub , K. Padberg , M. Kivala , J. Phys. Org. Chem. 2020, 33, 1–27.

[23] M. Hirai , N. Tanaka , M. Sakai , S. Yamaguchi , Chem. Rev. 2019, 119, 8291–8331. 30860363 10.1021/acs.chemrev.8b00637

[24] Z. Fang , R. D. Webster , M. Samoc , Y. H. Lai , RSC Adv. 2013, 3, 17914–17917.

[25] X. Zheng , X. Wang , Y. Qiu , Y. Li , C. Zhou , Y. Sui , Y. Li , J. Ma , X. Wang , J. Am. Chem. Soc. 2013, 135, 14912–14915. 24053534 10.1021/ja407318h

[26] M. Renz , K. Theilacker , C. Lambert , M. Kaupp , J. Am. Chem. Soc. 2009, 131, 16292–16302. 19831383 10.1021/ja9070859

[27] M. Kaupp , M. Renz , M. Parthey , M. Stolte , F. Würthner , C. Lambert , Phys. Chem. Chem. Phys. 2011, 13, 16973–16986. 21881628 10.1039/c1cp21772k

[28] R. Thomas , S. Lakshmi , S. K. Pati , G. U. Kulkarni , J. Phys. Chem. B 2006, 110, 24674–24677. 17134230 10.1021/jp0655423

[29] M. Uebe , A. Ito , Chem. Asian J. 2019, 14, 1692–1696. 30698917 10.1002/asia.201900036

[30] P. Mayorga Burrezo , W. Zeng , M. Moos , M. Holzapfel , S. Canola , F. Negri , C. Rovira , J. Veciana , H. Phan , J. Wu , C. Lambert , J. Casado , Angew. Chem. Int. Ed. 2019, 58, 14467–14471; 10.1002/anie.201905657 31322792

[31] M. Uebe , K. Kaneda , S. Fukuzaki , A. Ito , Chem. Eur. J. 2019, 25, 15455–15462. 31647599 10.1002/chem.201903667

[32] J. E. Wertz , J. R. Bolton , Electron Spin Resonance: Elementary Theory and Practical Applications, Chapman and Hall, New York, 1986.

[33] M. Holzapfel , C. Lambert , D. Stalke , J. Chem. Soc. Perkin Trans. 2 2002, 1553–1561.

[34] C. Lambert , G. Nöll , J. Am. Chem. Soc. 1999, 121, 8434–8442.

[35] D. Sun , S. V. Rosokha , J. K. Kochi , Angew. Chem. Int. Ed. 2005, 44, 5133–5136; 10.1002/anie.200501005 16010702

[36] Deposition Numbers 2040608 (DTA), 2040609 (DTB), and 2040610 (DTT) contain the supplementary crystallographic data for this paper. These data are provided free of charge by the joint Cambridge Crystallographic Data Centre and Fachinformationszentrum Karlsruhe Access Structures service www.ccdc.cam.ac.uk/structures.

