# Supplementary material for: Pre‐Planarized Triphenylamine‐Based Linear Mixed‐Valence Charge‐Transfer Systems
Source: Angew Chem Int Ed Engl. 2021 Feb 3;60(12):6771–7. doi: 10.1002/anie.202014567 (PMC7986061; doi:10.1002/anie.202014567)
Supplement: Supplementary file 1 — Supplementary [file ANIE-60-6771-s001.pdf]

Angewandte Chemie
Eine Zeitschrift der Gesellschaft Deutscher Chemiker

Supporting Information

Pre-Planarized Triphenylamine-Based Linear Mixed-Valence Charge-Transfer Systems

Marcel Krug[+], Nina Fröhlich[+], Dominik Fehn, Alexander Vogel, Frank Rominger, Karsten Meyer, Timothy Clark, Milan Kivala, and Dirk M. Guldi**

anie_202014567_sm_miscellaneous_information.pdf

Supporting Information

Contents

1 Chemical structures 2
2 Methods and materials 4
3 Experimental procedures 6
4 Nuclear magnetic resonance spectra 13
5 X-Ray crystallographic analysis 25
6 Electrochemistry 35
6.1 Square wave voltammetry 35
6.2 Cyclic voltammetry 39
6.3 Spectroelectrochemistry 42
7 Steady-state absorption and fluorescence spectroscopy 48
7.1 Characterization of the neutral compounds 48
7.2 Titrations with $SbCl_5$ 50
7.3 Mulliken-Hush analysis 51
8 Computational studies 55
8.1 Computational methods 55
8.2 Ground state properties 56
8.2.1 Potential energy scans 56
8.2.2 Ground state geometries 61
8.2.3 Orbital correlation diagrams 70
8.2.4 Electrostatic potential maps 73
8.3 Time-dependent calculations 76
9 EPR-Spectroscopy 85
9.1 Overview 85
9.2 $DTA^{•+}$ 87
9.3 $DTB^{•+}$ 91
9.4 $DTT^{•+}$ 98
References 102

1 Chemical structures

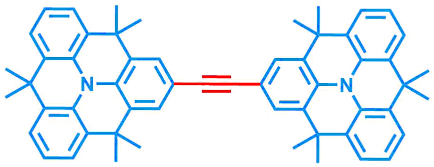

DTA

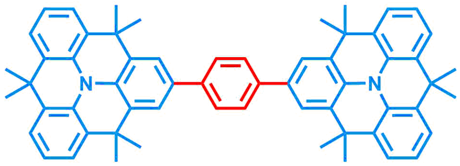

DTB

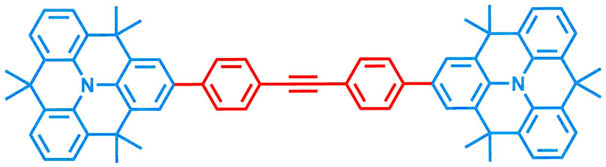

DTT

Figure S1: Chemical structures of *N*-heterotriangulene dimers DTA, DTB, and DTT. The parent *N*-heterotriangulenes are colored in light blue and the linkers in red.

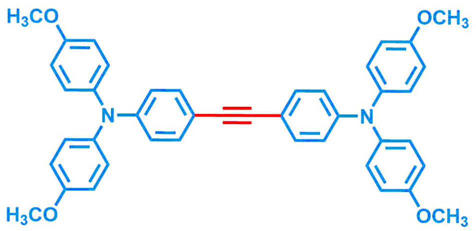

R1

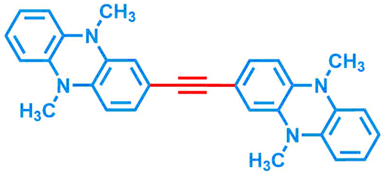

R2

Figure S2: Chemical structures of the references R1 and R2. The parent building blocks are colored in light blue and the linkers in red.[1]

2 Methods and materials

All reactions involving oxygen- or moisture-sensitive compounds were carried out in dry reaction vessels under an inert atmosphere of nitrogen or argon using anhydrous solvents and standard Schlenk techniques. All oxygen- and moisture-sensitive liquids and anhydrous solvents were transferred *via* a syringe or a stainless steel cannula. Analytical TLC analysis was performed on aluminum plates coated with 0.20 mm silica gel containing a fluorescent indicator (Macherey-Nagel, ALUGRAM®, SIL G/UV_{254}) or on aluminum plates coated with 0.20 mm aluminum oxide containing a fluorescent indicator (Macherey-Nagel, ALUGRAM®, ALOX N/UV_{254}). TLC plates were visualized by exposure to ultraviolet light (λ = 254 nm and 366 nm). Column chromatography was performed on silica gel (Macherey-Nagel, M-N Silica Gel 60A, 230-400 mesh).

Reagents were purchased at reagent grade from commercial suppliers and used without further purification. $MgSO_4$ was used as the drying agent after aqueous workup.

^{1}H- and ^{13}C-NMR spectra were recorded on a Bruker Avance 600 (Bruker, 600 MHz, for ^{1}H, 150 MHz for ^{13}C), a Bruker Avance 400 (Bruker, 400 MHz for ^{1}H, 100 MHz for ^{13}C) and a Bruker Avance 300 (Bruker, 300 MHz for ^{1}H, 75 MHz and for ^{13}C) spectrometer. Chemical shifts (δ) are reported in ppm and were referenced to the residual solvent signal as an internal reference (CD_2Cl_2: 5.32 ppm for ^{1}H, 53.84 for ^{13}C; $CDCl_3$ 7.26 for ^{1}H, 77.16 for ^{13}C. Coupling constants (J) are given in Hz as observed and the apparent resonance multiplicity is reported as s (singlet), d (doublet), t (triplet) or m (multiplet). All signals of solvents and impurities are assigned according to literature.[2] All spectra were recorded at ambient probe temperature if not otherwise stated.

Mass spectra were obtained from a MicroTOF II (Bruker, HR ESI and APPI) or a UltraflexTOF/TOF (Bruker, HR MALDI) mass spectrometer at the Institute of Organic Chemistry, University of Erlangen-Nürnberg.

IR spectra were recorded on a 660-IR (Varian, ATR mode) spectrometer and characteristic IR absorptions were reported in cm^{-1} and labelled as strong (s), medium (m), and weak (w).

Melting points (Mp) were determined on a Büchi M-560 melting point apparatus in open capillaries.

Steady state absorption spectra were obtained using a PerkinElmer Lambda 2 dual beam absorption spectrometer (250 – 1100 nm) or a Varian Cary 5000 (250 – 3000 nm). Steady state emission spectra were recorded using a Horiba Jobin Yvon FluoroMax-3 emission spectrometer with 1.5 nm spectral bandwidth and 0.25 s integration time. Samples were contained in 10 x 10 mm quartz cuvettes (Hellma Analytics, QS and QX).

Electrochemical experiments were performed in a three electrode setup with a platinum working electrode, a silver quasi-reference electrode and a platinum counter electrode. A FRA 2 µAutolab Type III potentiostat was used to apply the external potential. The potential scale was referenced versus the Fc/Fc^{+} redox couple. $TBAPF_6$ was used as the supporting electrolyte in 0.2 M concentration. Prior to the measurement, the solution was degassed with argon for at least 15 min to prevent any effects of oxygen. For the peak analysis a background correction was applied.

Spectroelectrochemical assays in the UV/vis/nIR region were performed in a 2 mm quartz cuvette with a platinum mesh as working electrode, a silver wire as quasi-reference electrode and a platinum wire as counter electrode. Absorption spectra were recorded on a Varian Cary 5000. For the IR region a

commercially available thin layer cell (OMNI-CELL SPECAC) with CaF_2 windows and platinum mesh as working electrode, silver wire as quasi-reference electrode and platinum counter electrode was used. The IR spectra were recorded on a Shimadzu IRPrestige-21 spectrometer in transmission mode and an average of 20 spectra. In both cases a FRA 2 µAutolab Type III potentiostat was used to apply the external potential.

EPR spectra were recorded on a JEOL continuous wave spectrometer JES-FA200, equipped with an X-band Gunn diode oscillator bridge, a cylindrical mode cavity, and an N_2 cryostat. The samples were measured in liquid and frozen solution, in a quartz glass capillary, placed inside a quartz glass EPR tube, at 293, 260, 250, 230, 200, 150, and 95 K, respectively. The spectra shown were measured using the following parameters: microwave frequency ν = 8.959 GHz, modulation width 1.0 to 0.01 mT, microwave power 1.0 mW, modulation frequency 100 kHz, time constant of 0.1 s. Simulation of the measured spectra was carried out with the SL.esimX software package written by Dr. Eckhard Bill (MPI Mülheim/Ruhr) on the basis of a spin-Hamiltonian description of the electronic ground state:

$$\hat{H} = D\left(\hat{S}_z^2 - \frac{1}{3}S(S+1) + \frac{E}{D}\left(\hat{S}_x^2 - \hat{S}_y^2\right)\right) + \mu_B \underline{g}\vec{S}\ .$$

Here, S represents the total spin quantum number of the coupled system, D and E/D are the axial and rhombic zero-field parameters, respectively, and $\underline{g}$ is the g-matrix. Calculations are based on the S = 5/2 routines developed by Gaffney and Silverstone.[3]

3 Experimental procedures

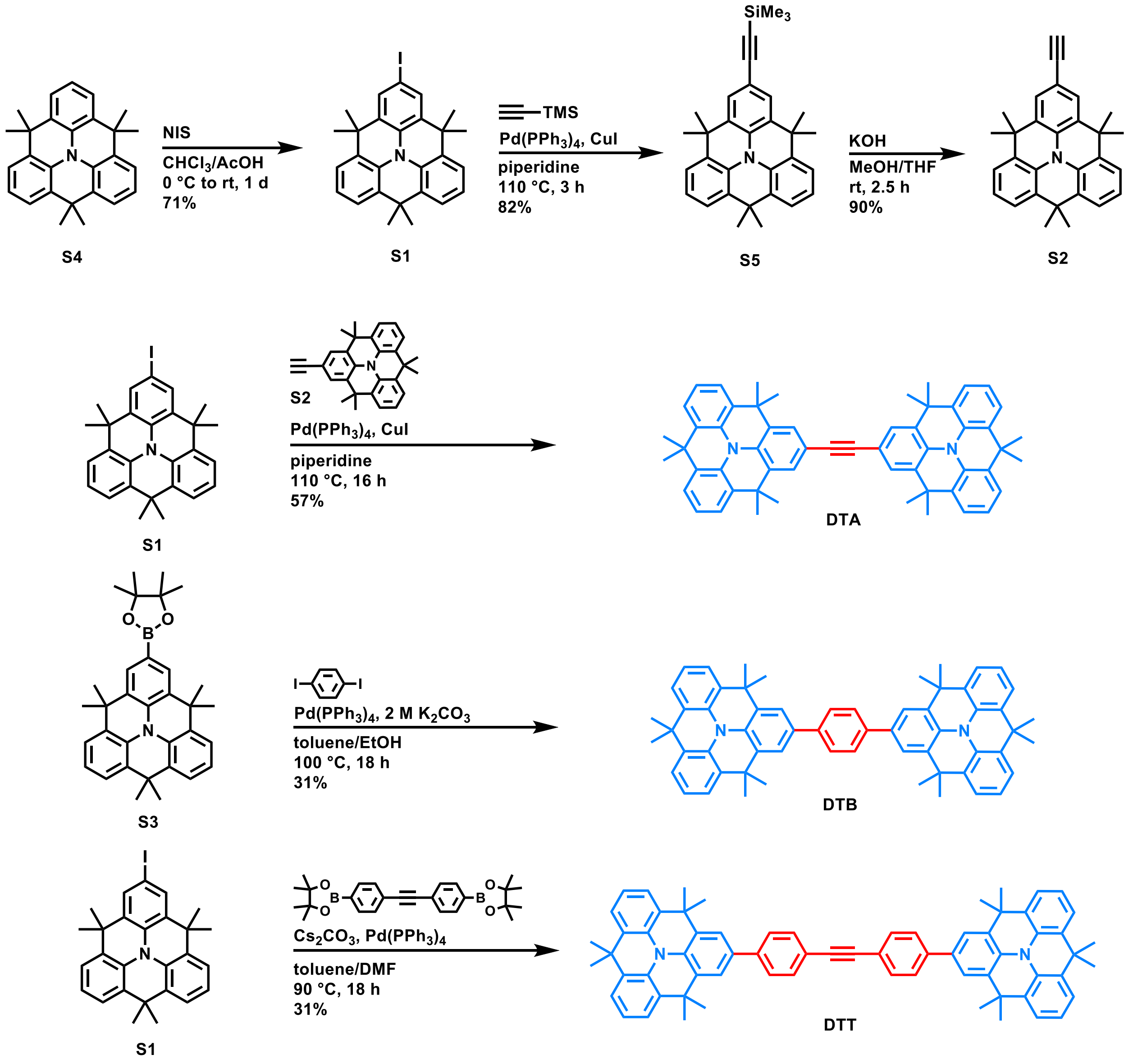

Scheme S1. Precursor synthesis and synthetic pathway towards DTA, DTB, and DTT.

N-HTA (**S4**) and *N*-HTA boronic acid pinacol ester (**S3**) were prepared according to literature known procedures.[4,5]

Compound S1

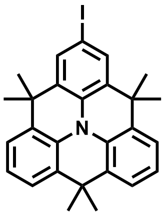

S1

To a solution of **S4** (211 mg, 577 μmol) in $CHCl_3$ (6 mL) was added AcOH (3 mL) and *N*-iodosuccinimide (136 mg, 180 μmol) in small portions at 0 °C. The reaction mixture was allowed to warm up to rt and was stirred for 24 h in the dark. Afterwards the reaction mixture was poured into ice water and was neutralized with sat. aq. $NaHCO_3$. The organic layer was washed with sat. aq. $Na_2S_2O_4$ (20mL) and sat. aq. NaCl (20 mL), dried ($MgSO_4$), filtered, and the solvents were removed under reduced pressure. Column chromatography (SiO_2, hexanes/CH_2Cl_2 8:1) provided **S1** as colorless solid (201 mg, 71%).

Mp 61–64°C.

R_f = 0.51 (SiO_2, hexanes/CH_2Cl_2 8:1).

^{1}H-NMR (300 MHz, CD_2Cl_2) δ 7.63 (s, 2H), 7.42–7.36 (m, 4H), 7.13 (t, J = 7.7 Hz, 2H), 1.61 (s, 6H), 1.58 (s, 12H) ppm.

^{13}C-NMR (75 MHz, CD_2Cl_2) δ 132.7, 132.5, 131.9, 130.4, 129.9, 124.2, 123.8, 123.6, 86.1, 35.8, 33.4, 32.9 ppm.

IR (ATR) $\tilde{\nu}$ 2961 (m), 2920 (w), 2858 (w), 1422 (s), 1283 (s), 1209 (m), 1124 (m), 1080 (w), 858 (w), 791 (w), 740 (s), 651 (w), 628 (w) cm^{-1}.

MALDI HRMS (dctb, positive mode) calcd. for $C_{27}H_{26}IN$ 491.1104 $[M]^+$, found 491.1091.

Compound S5

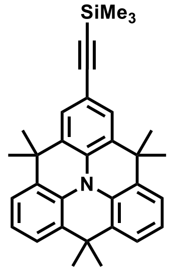

S5

To a deoxygenated mixture of **S1** (30 mg, 61 μmol), $Pd(PPh_3)_4$ (2 mg, 1.8 μmol) and CuI (2 mg, 9.8 μmol) in piperidine (2 mL), TMS-acetylene (6 mg, 9 μL, 64 μmol) was slowly added. The mixture was heated to 110 °C for 3 h. After cooling to rt, H_2O (5 mL) and CH_2Cl_2 (10 mL) were added to the reaction mixture. The organic layer was separated and washed with sat. aq. NH_4Cl (1 x 20 mL) and sat. aq. NaCl (1 x 20 mL), dried ($MgSO_4$), filtered, and the solvents were removed under reduced pressure. Column chromatography (SiO_2, hexanes/CH_2Cl_2 4:1) provided **S5** as a colorless solid (23 mg, 82%).

Mp 228–230 °C.

R_f = 0.60 (SiO_2, hexanes/CH_2Cl_2 4:1).

^{1}H-NMR (300 MHz, CD_2Cl_2) δ 7.47 (s, 2H), 7.42–7.37 (m, 4H), 7.15 (t, J = 7.7 Hz, 2H), 1.62 (s, 6H), 1.60 (s, 12H), 0.27 (s, 9H) ppm.

^{13}C-NMR (75 MHz, CD_2Cl_2) δ 131.8, 130.5, 130.23, 130.15, 127.4, 124.3, 123.8, 123.7,117.3, 106.2, 93.2, 35.8, 33.6, 32.9, 0.2 ppm (2 peaks coincident or not observed).

IR (ATR) $\tilde{\nu}$ 3081 (w), 2957 (m), 2139 (m), 1592 (w), 1420 (s), 1270 (m), 1124 (w), 965 (m), 840 (s), 745 (s), 694 (w) cm^{-1}.

MALDI HRMS (dctb, positive mode) calcd. for $C_{32}H_{35}NSi$ 461.2533 $[M]^+$, found 461.2533.

Compound S2

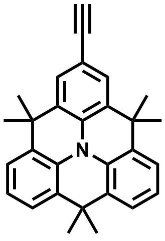

S2

To a solution of compound **S5** (60 mg, 130 μmol) in MeOH/THF (1:1, 6 mL) was added a solution of KOH (182 mg, 2.85 mmol) in water (0.5 mL). The reaction mixture was stirred at rt for 2.5 h and subsequently was extracted with CH_2Cl_2. (2 x 15 mL) The combined organic layers were washed with sat. aq. NaCl (1 x 20 mL), dried ($MgSO_4$), filtered, and the solvents were removed under reduced pressure to provide **S2** as a colorless solid (45 mg, 90%).

Mp 54–56 °C.

R_f = 0.56 (SiO_2, hexanes/CH_2Cl_2 4:1).

^{1}H-NMR (400 MHz, $CDCl_3$) δ 7.51 (s, 2H), 7.41–7.37 (m, 4H), 7.15 (t, J = 7.7 Hz, 2H), 3.11 (s, 1H), 1.64 (s, 6H), 1.61 (s, 12H)

^{13}C-NMR (100 MHz, $CDCl_3$) δ 132.8, 131.5, 130.2, 129.9, 127.4, 124.0, 123.5, 123.4, 119.9, 115.8, 76.3, 68.1, 35.6, 33.6, 32.9, 25.7 ppm.

IR (ATR) $\tilde{\nu}$ 3287 (m), 3077 (w), 2962 (m), 2922 (m), 2858 (w), 2099 (w), 1798 (m), 1591 (m), 1425 (s), 1289 (s), 1212 (m), 1128 (m), 878 (w), 793 (w), 750 (s) cm^{-1}.

APPI HRMS (CH_2Cl_2/ACN, positive mode) calcd. for $C_{29}H_{27}N$ 389.2138 $[M]^+$, found 389.2138.

Dimer DTA

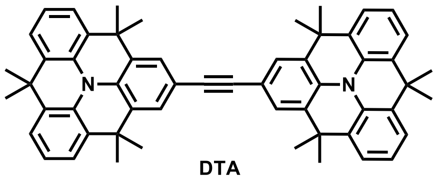

DTA

To a deoxygenated mixture of **S1** (41 mg, 83 μmol), $Pd(PPh_3)_4$ (3 mg, 2.5 μmol) and CuI (3 mg, 13 μmol) in piperidine (3 mL), **S2** (33 mg, 83 μmol) was slowly added. The mixture was heated to 110 °C for 16 h. After cooling to rt, H_2O (5 mL) and CH_2Cl_2 (10 mL) were added to the reaction mixture. The organic layer was separated and washed with sat. aq. NH_4Cl (25 mL) and sat. aq. NaCl (25 mL), dried ($MgSO_4$), filtered, and the solvents were removed under reduced pressure. Flash column chromatography (SiO_2, hexanes/CH_2Cl_2 3:1) provided DTA as a pale yellow solid (36 mg, 57%).

Mp 257–259 °C.

R_f = 0.56 (SiO_2, hexanes/CH_2Cl_2 3:1).

^{1}H-NMR (400 MHz, $CDCl_3$) δ 7.59 (s, 4H), 7.40 (d, J = 7.7 Hz, 8H), 7.15 (t, J = 7.7 Hz, 4H), 1.66 (br s, 36H) ppm.

^{13}C-NMR (100 MHz, $CDCl_3$) δ 132.2, 131.6, 130.2, 130.0, 129.9, 126.8, 123.9, 123.6, 123.3, 117.5, 89.3, 35.7, 35.6, 33.6, 33.0 ppm.

IR (ATR) $\tilde{\nu}$ 3078 (w), 2965 (m), 2925 (m), 2576 (w), 2362 (w), 2206 (w), 1591 (w), 1428 (s), 1349 (m), 1292 (s), 1208 (s), 1124 (w), 1080 (w), 984 (w), 886 (w), 793 (m), 743 (s), 663 (w) cm^{-1}.

MALDI HRMS (positive mode) calcd. for $C_{56}H_{52}N_2$ $[M]^+$ 752.4125, found 752.4144

Dimer DTB

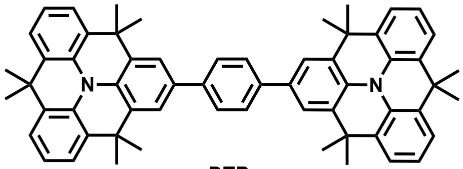

DTB

To a deoxygenated mixture of 1,4-iodobenzene (9.0 mg, 27 μmol) and **S3** (29 mg, 60 μmol) in toluene/EtOH (2:1, 3 mL) was added a degassed aq. solution of K_2CO_3 (2 M, 0.4 mL) and $Pd(PPh_3)_4$ (4.7 mg, 4.2 μmol). The reaction mixture was stirred at 100 °C for 18 h. The reaction was quenched with H_2O (3 mL) and extracted with CH_2Cl_2 (2 x 10 mL). The combined organic phases were dried ($MgSO_4$), filtered, and the solvents were removed under reduced pressure. Flash column chromatography (SiO_2, hexanes → CH_2Cl_2) provided DTB as a colorless solid (6.7 mg, 31%).

Mp 356–359 °C.

R_f = 0.27 (SiO_2, hexanes/CH_2Cl_2 4:1).

^{1}H-NMR (600 MHz, CD_2Cl_2) δ 7.77 (s, 4H), 7.70 (s, 4H), 7.43 (td, J = 7.4, 1.6 Hz, 8H), 7.15 (t, J = 7.7 Hz, 4H), 1.71 (s, 24H), 1.64 (s, 12H) ppm.

^{13}C-NMR (150 MHz, CD_2Cl_2) δ 139.8, 135.1, 132.1, 130.8, 130.3, 130.2, 127.2, 124.0, 123.4, 122.4, 36.1, 35.9, 33.4, 33.3 ppm (1 peak coincident or not observed).

IR (ATR) $\tilde{\nu}$ 2959 (w), 2920 (w), 2854 (w), 1645 (w), 1426 (s), 1293 (m), 1122 (m), 830 (w) cm^{-1}.

APPI HRMS (CH_2Cl_2, positive mode) calcd. for $C_{60}H_{57}N_2$ $[M+H]^+$ 805.4516, found 805.4525.

Dimer DTT

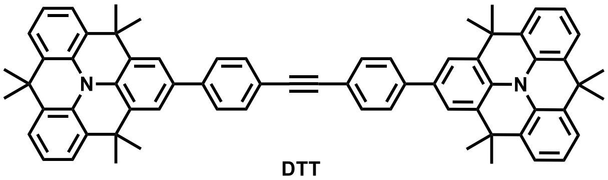

DTT

To a deoxygenated mixture of **S1** (61.2 mg, 124 μmol), 4,4'-(acetylene-1,2-diyl)bis(phenylboronic acid pinacol ester) (21.4 mg, 49.8 μmol) and Cs_2CO_3 (64.8 mg, 199 μmol) in toluene/DMF (2:1, 3 mL) was added $Pd(PPh_3)_4$ (5.7 mg, 5.0 μmol). The reaction mixture was stirred at 90 °C for 16 h. The solvents were removed under reduced pressure. Flash column chromatography (SiO_2, hexanes → CH_2Cl_2) provided DTT as a pale yellow solid (19.2 mg, 43%).

Mp 380–382 °C (decomposition).

R_f = 0.69 (SiO_2, hexanes/CH_2Cl_2 1:1).

^{1}H-NMR (400 MHz, $CDCl_3$) δ 7.66 (s, 8H), 7.63 (s, 4H), 7.43 (m, 8H), 7.15 (t, J = 7.7 Hz, 4H), 1.71 (s, 24H), 1.65 (s, 12H) ppm.

^{13}C-NMR (100 MHz, $CDCl_3$) δ 134.5, 132.2, 131.8, 130.5, 130.1, 129.9, 126.5, 123.8, 123.7, 123.2, 122.2, 121.8, 90,3, 35.9, 35.7, 33.4 ppm (1 peak coincident or not observed).

APPI HRMS (toluene, positive mode) calcd. for $C_{68}H_{60}N_2$ $[M]^+$ 904.4751, found 904.4752.

4 Nuclear magnetic resonance spectra

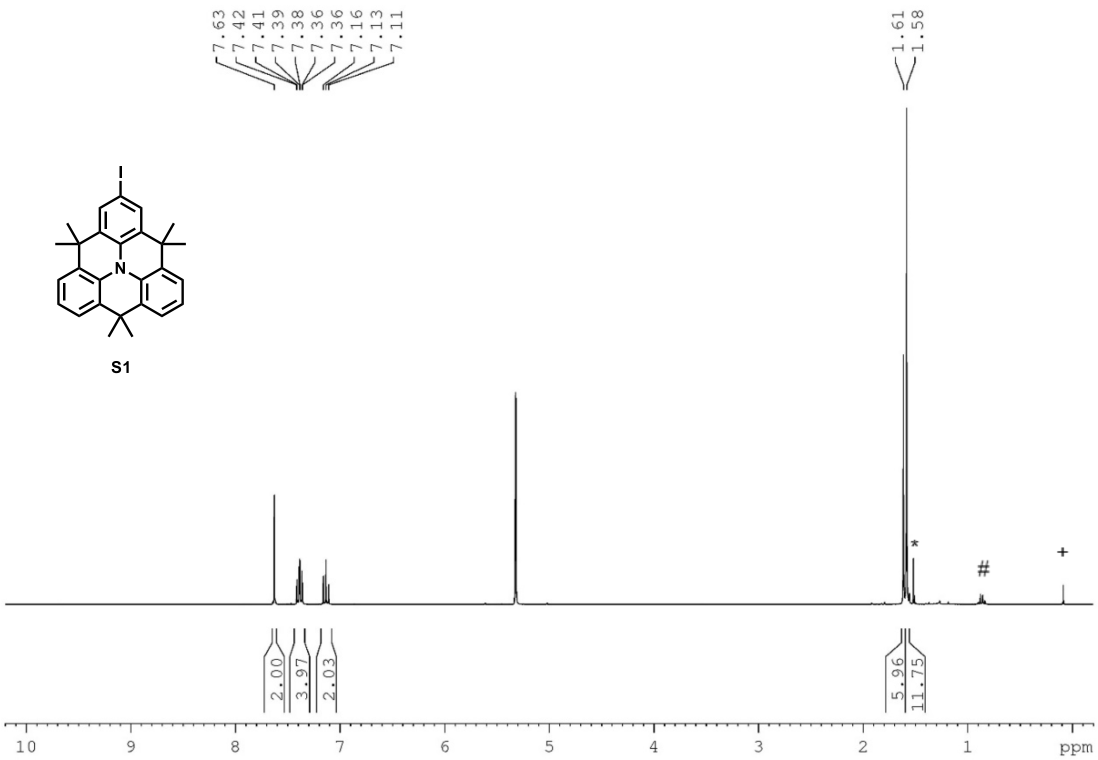

Figure S3: ^{1}H-NMR (300 MHz, CD_2Cl_2) spectrum of **S1**.$^{+}$grease, $^{\#}$hexanes,*H_2O.

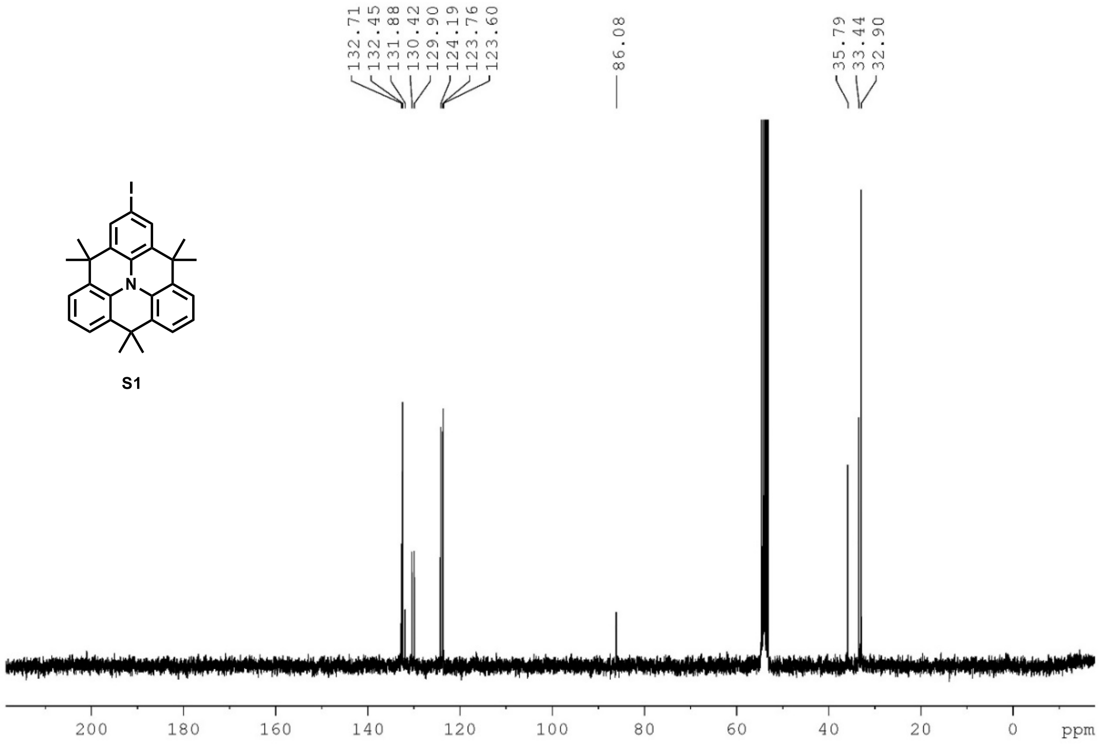

Figure S4: ^{13}C-NMR (75 MHz, CD_2Cl_2) spectrum of **S1**.

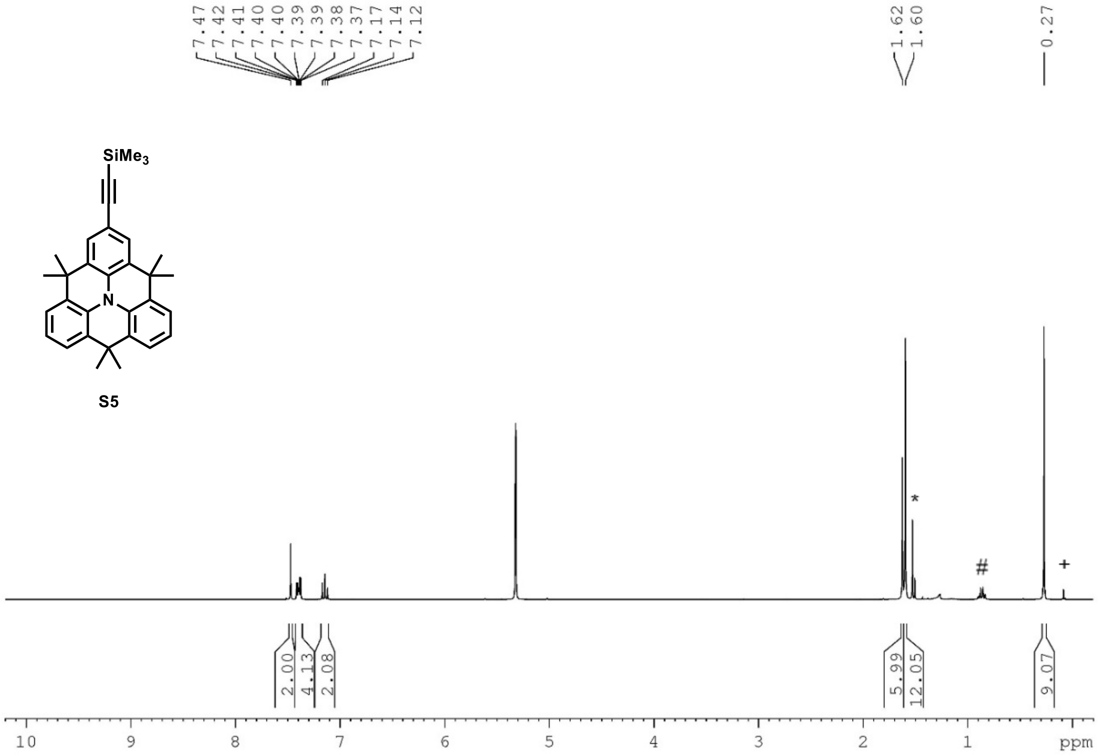

Figure S5: ^{1}H-NMR (300 MHz, CD_2Cl_2) spectrum of **S5**. $^+$grease, $^\#$hexanes, *H_2O.

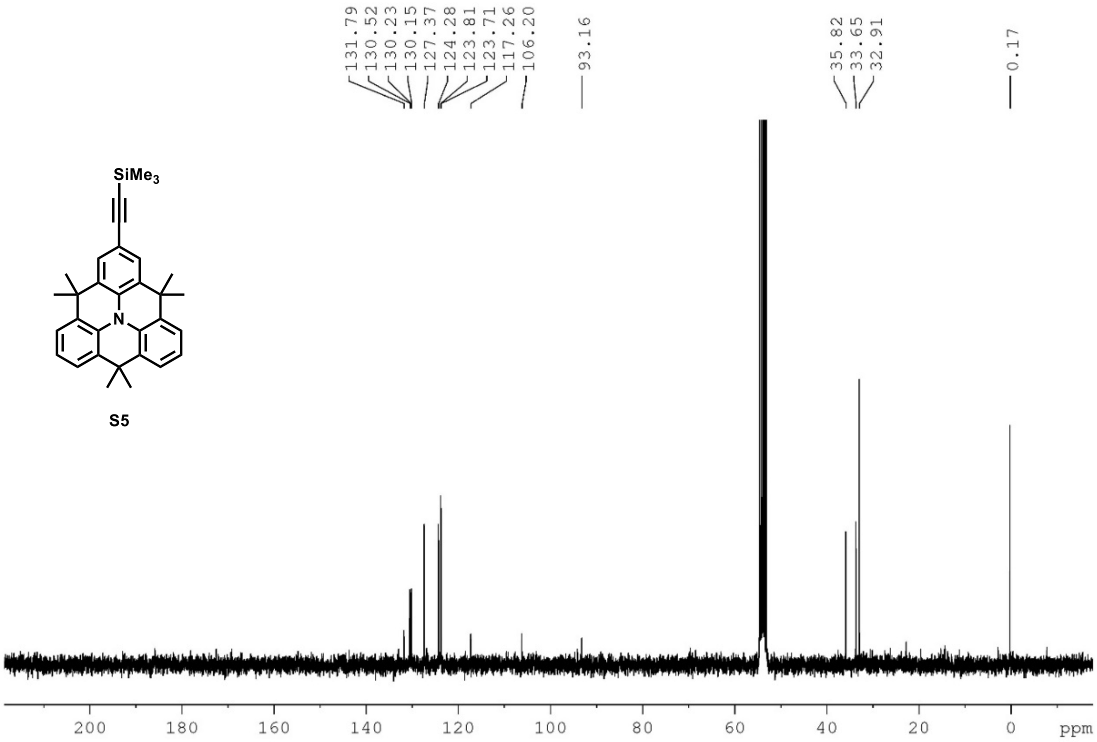

Figure S6: ^{13}C-NMR (75 MHz, CD_2Cl_2) spectrum of **S5**.

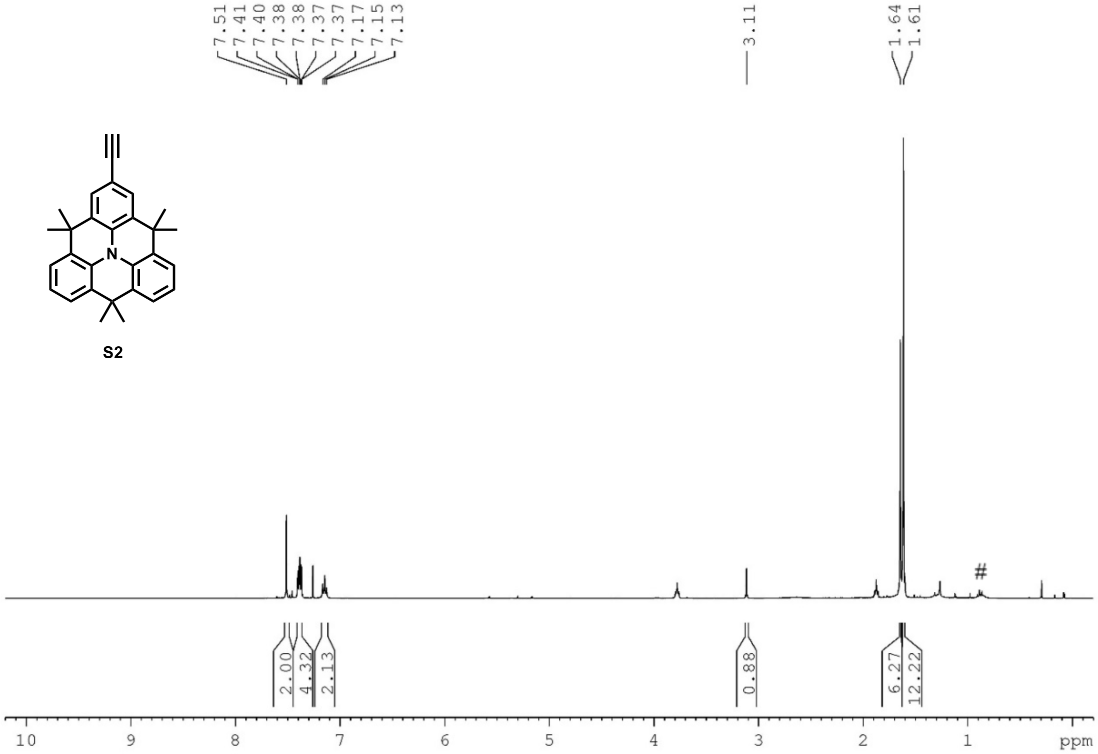

Figure S7: ^{1}H-NMR (400 MHz, $CDCl_3$) spectrum of **S2**. $^{\#}$hexanes.

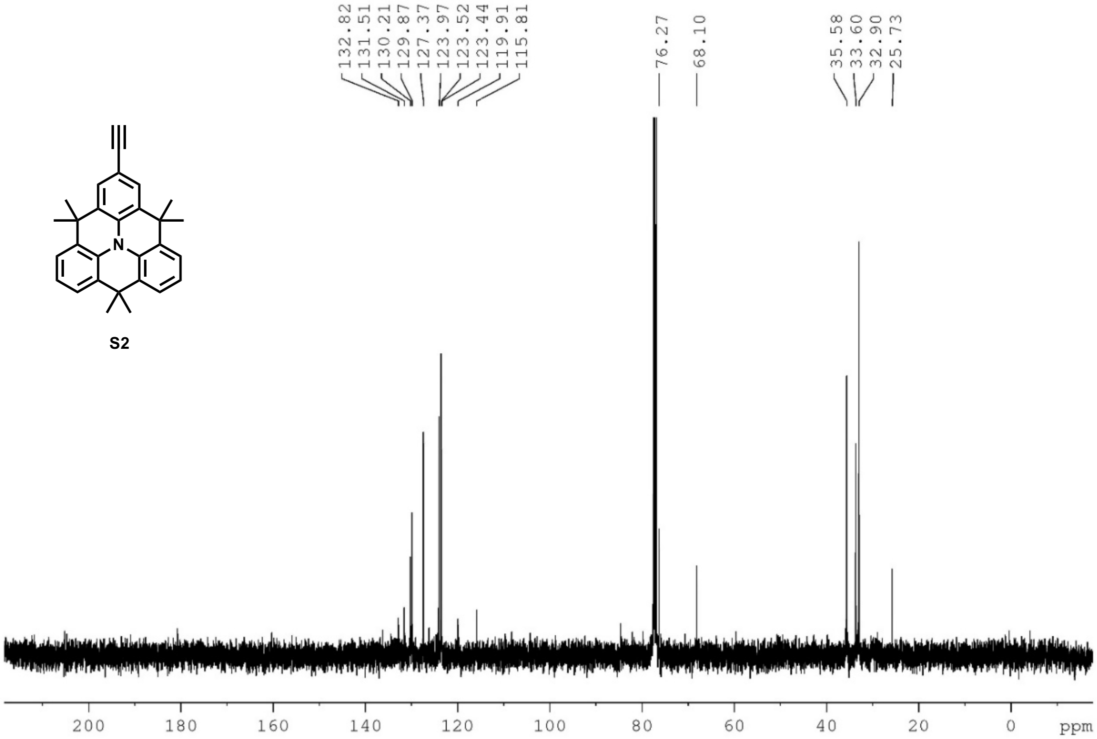

Figure S8: ^{13}C-NMR (100 MHz, CD_3Cl_3) spectrum of **S2**.

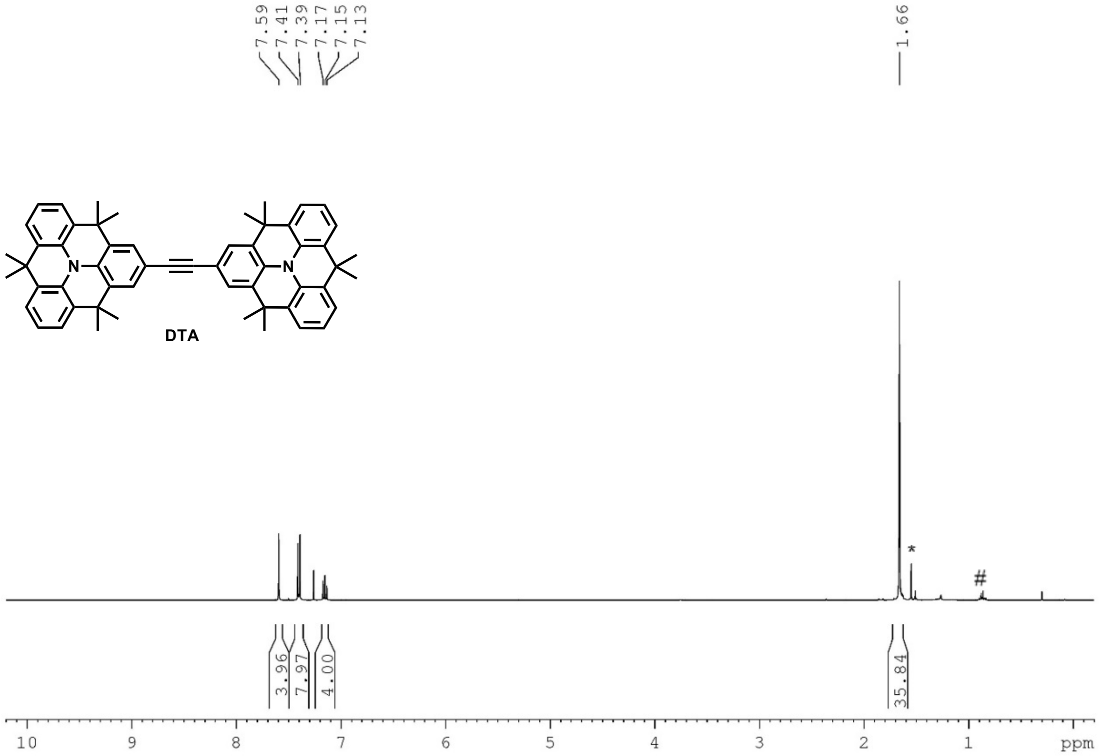

Figure S9: ^{1}H-NMR (400 MHz, $CDCl_3$) of DTA. #hexanes, *H_2O.

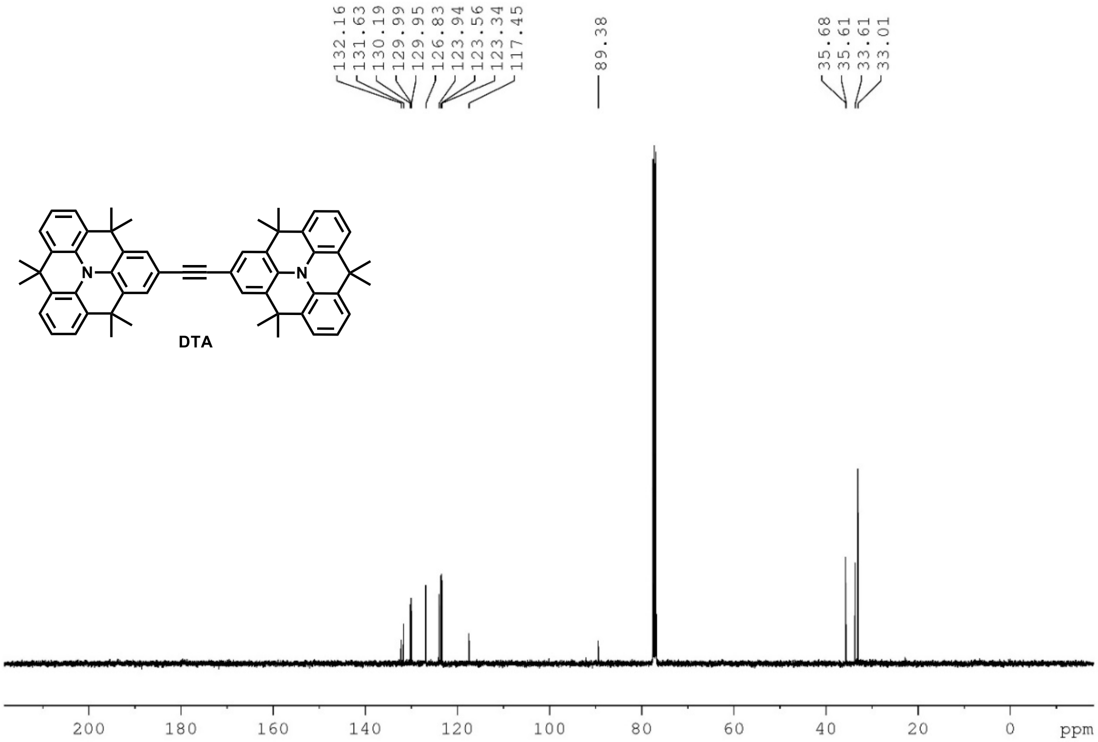

Figure S10: ^{13}C-NMR (100 MHz, $CDCl_3$) of DTA.

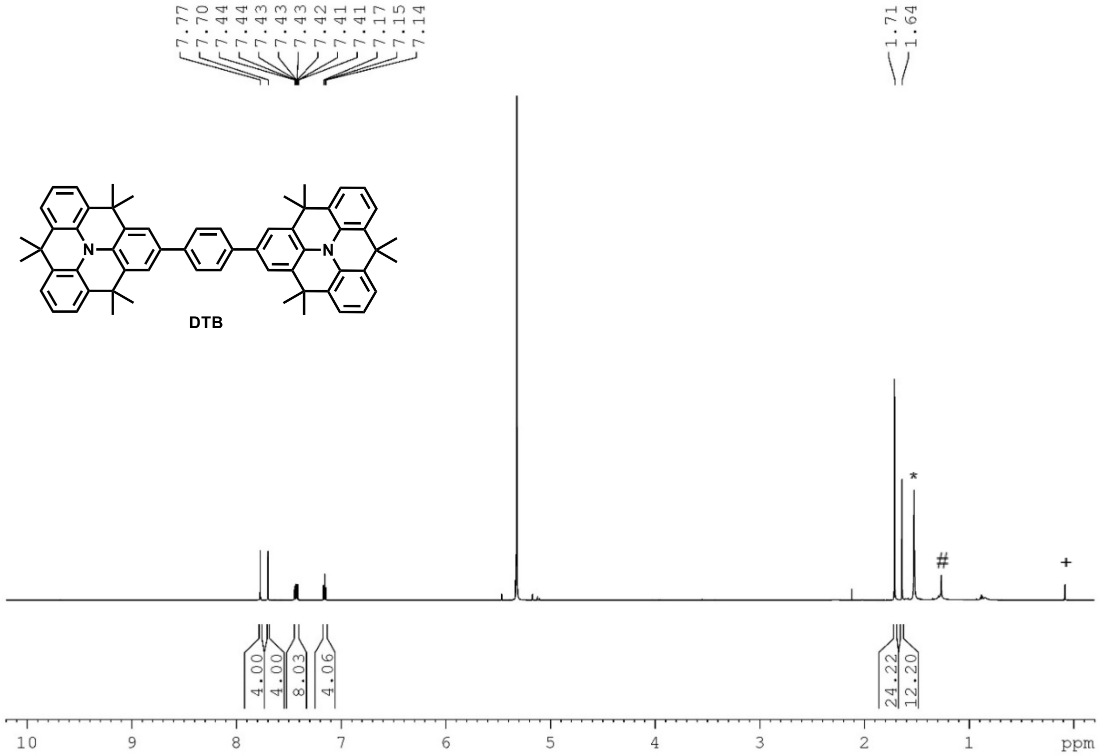

Figure S11: ^{1}H-NMR (600 MHz, CD_2Cl_2) of DTB. $^{+}$grease, $^{\#}$hexanes, *H_2O.

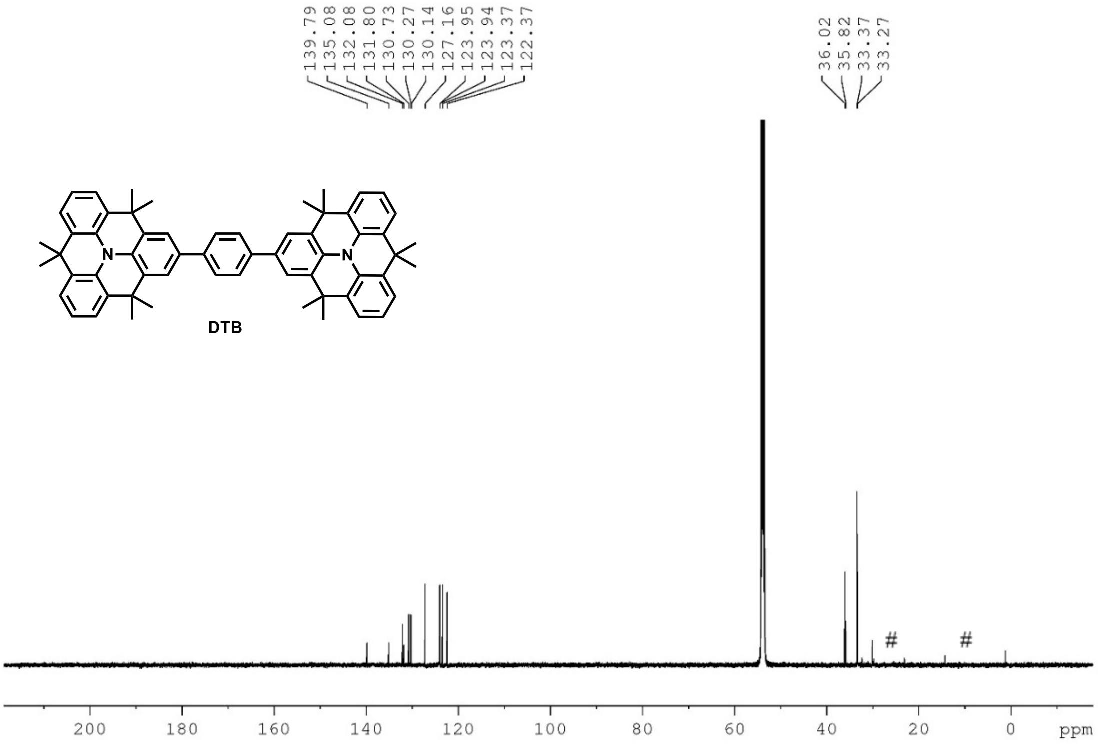

Figure S12: ^{13}C-NMR (150 MHz, CD_2Cl_2) of DTB. #hexanes.

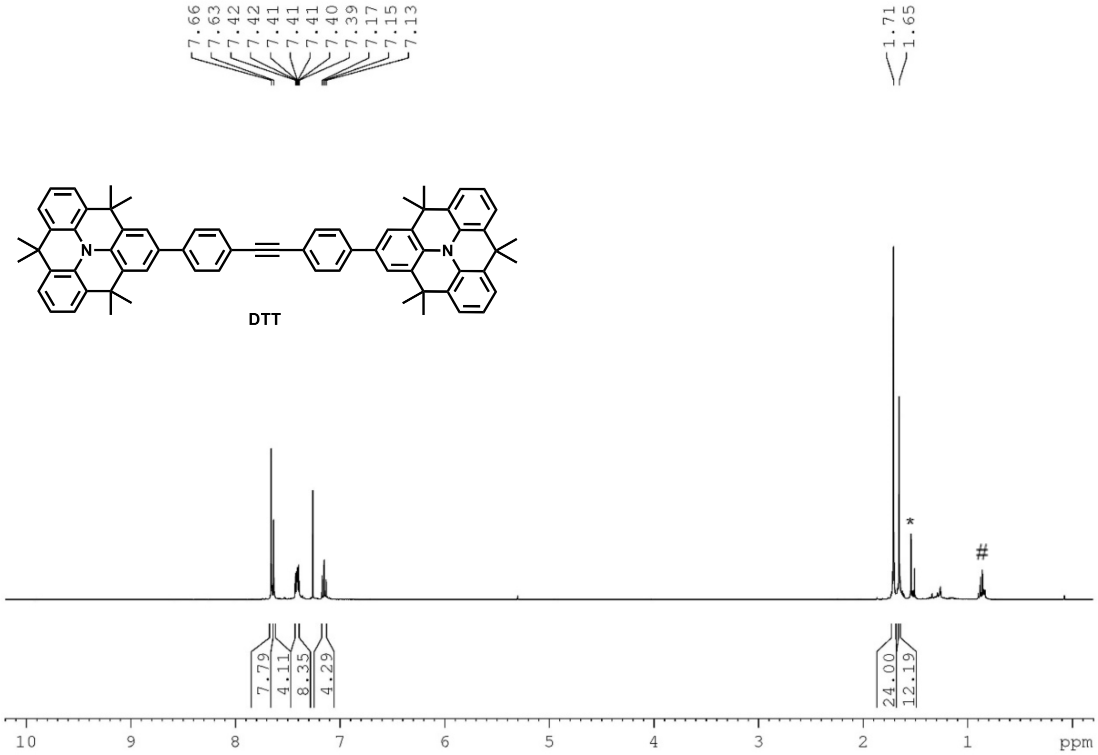

Figure S13: ^{1}H-NMR (400 MHz, $CDCl_3$) of DTT. #hexanes, *H_2O.

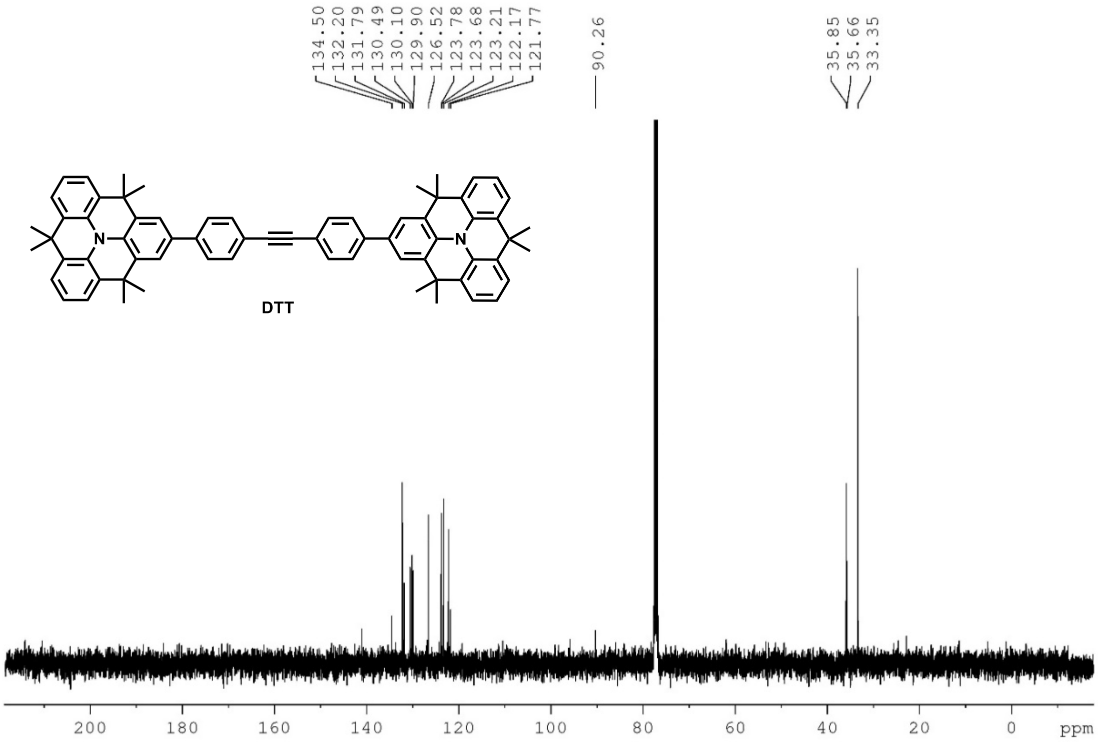

Figure S14: ^{13}C-NMR (100 MHz, $CDCl_3$) of DTT.

5 X-Ray crystallographic analysis

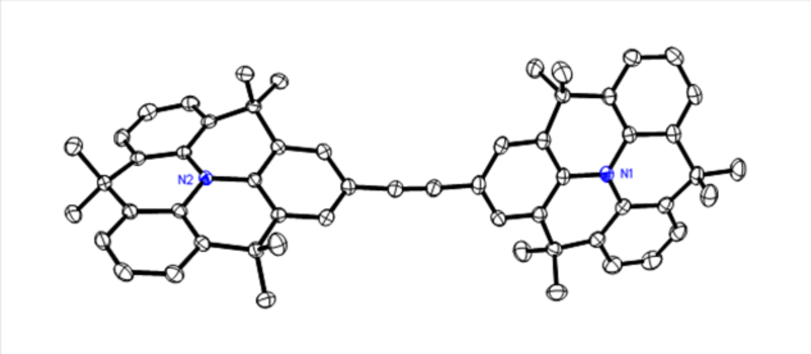

Figure S15: Molecular structure of **DTA** revealed by X-ray crystallographic analysis.

Dimer DTA: Single crystals of **DTA** were obtained by slow liquid diffusion of MeOH into the CH_2Cl_2 solution of the compounds at rt. CCDC 2040608 contains the supplementary crystallographic data for this paper. The data can be obtained free of charge from The Cambridge Crystallographic Data Centre via www.ccdc.cam.ac.uk/structures.

Table S1: Crystal data and structure refinement for **DTA**.

Empirical formula	$C_{56}H_{52}N_2$
Formula weight	752.99
Temperature	200(2) K
Wavelength	0.71073 Å
Crystal system	monoclinic
Space group	$P2_1/c$
Z	4
Unit cell dimensions	a = 21.1303(10) Å, α =90 deg.
	b = 10.6238(4) Å, β =95.2851(15) deg.
	c = 19.0321(8) Å, γ =90 deg.
Volume	4254.2(3) Å^3
Density (calculated)	1.18 g/cm^3
Absorption coefficient	0.07 mm^{-1}
Crystal shape	plate
Crystal size	0.260 x 0.123 x 0.045 mm^3
Crystal colour	yellow
Theta range for data collection	1.9 to 26.8 deg.
Index ranges	$-26 \leq h \leq 26$, $-13 \leq k \leq 13$, $-24 \leq l \leq 24$
Reflections collected	42126
Independent reflections	9103 (R(int) = 0.0769)
Observed reflections	6048 (I > 2□(I))
Absorption correction	Semi-empirical from equivalents
Max. and min. transmission	0.96 and 0.91
Refinement method	Full-matrix least-squares on F^2
Data/restraints/parameters	9103 / 0 / 536
Goodness-of-fit on F^2	1.02
Final R indices (I>2sigma(I))	R1 = 0.051, wR2 = 0.112
Largest diff. peak and hole	0.22 and -0.22 eÅ^{-3}

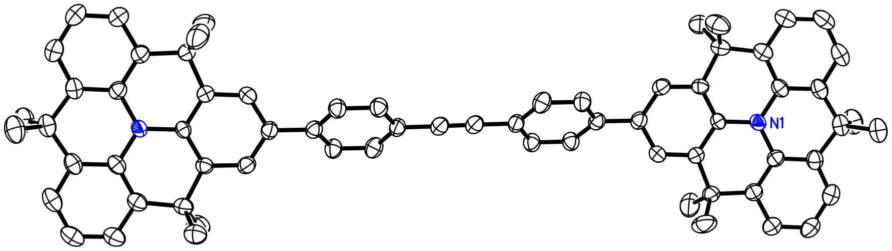

Figure S16: Molecular structure of DTA revealed by X-ray crystallographic analysis.

Dimer DTT: Single crystals of DTT were by obtained slow liquid diffusion of MeOH into the CH_2Cl_2 solution of the compounds at rt. CCDC 2040610 contains the supplementary crystallographic data for this paper. The data can be obtained free of charge from The Cambridge Crystallographic Data Centre via www.ccdc.cam.ac.uk/structures.

Table S2: Crystal data and structure refinement for DTT.

Empirical formula	$C_{68}H_{60}N_2$
Formula weight	905.18
Temperature	200(2) K
Wavelength	1.54178 Å
Crystal system	monoclinic
Space group	*C*2/*c*
Z	4
Unit cell dimensions	a = 41.479(3) Å α = 90 deg.
	b = 5.9016(4) Å β = 104.771(5) deg.
	c = 20.5119(12) Å γ = 90 deg.
Volume	4855.2(6) $Å^3$
Density (calculated)	1.24 g/cm^3
Absorption coefficient	0.54 mm^{-1}
Crystal shape	plate
Crystal size	0.115 x 0.083 x 0.020 mm^3
Crystal colour	yellow
Theta range for data collection	4.4 to 62.4 deg.
Index ranges	$-46 \leq h \leq 47$, $-6 \leq k \leq 6$, $-12 \leq l \leq 23$
Reflections collected	14286
Independent reflections	3830 (R(int) = 0.0930)
Observed reflections	1821 ($I > 2\sigma(I)$)
Absorption correction	Semi-empirical from equivalents
Max. and min. transmission	1.42 and 0.66
Refinement method	Full-matrix least-squares on F^2
Data/restraints/parameters	3830 / 0 / 322
Goodness-of-fit on F^2	0.90
Final R indices (I>2sigma(I))	R1 = 0.050, wR2 = 0.093
Largest diff. peak and hole	0.20 and -0.16 $eÅ^{-3}$

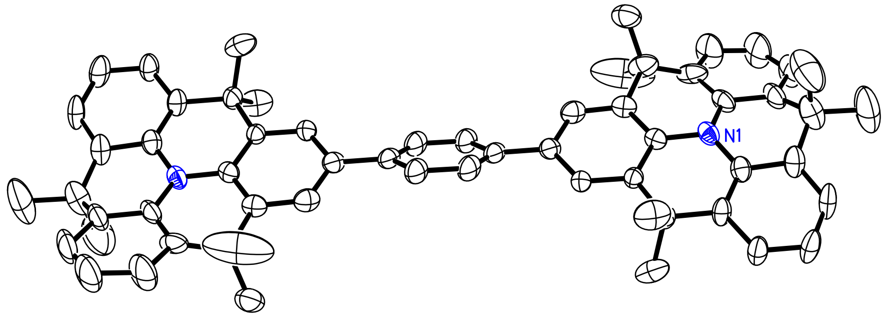

Figure S17: Molecular structure of DTB revealed by X-ray crystallographic analysis.

Dimer DTB: Single crystals of DTB were obtained by slow evaporation of CD_2Cl_2 at rt. CCDC 2040609 contains the supplementary crystallographic data for this paper. The data can be obtained free of charge from The Cambridge Crystallographic Data Centre via www.ccdc.cam.ac.uk/structures.

Table S3: Crystal data and structure refinement for DTB.

Empirical formula	$C_{60}H_{56}N_2$
Formula weight	805.06
Temperature	200(2) K
Wavelength	1.54178 Å
Crystal system	monoclinic
Space group	$P2_1/c$
Z	2
Unit cell dimensions	a = 5.8853(5) Å α = 90 deg.
	b = 18.6913(11) Å β = 95.816(7) deg.
	c = 19.5504(18) Å γ = 90 deg.
Volume	2139.5(3) Å^3
Density (calculated)	1.25 g/cm^3
Absorption coefficient	0.54 mm^{-1}
Crystal shape	pole
Crystal size	0.190 x 0.030 x 0.022 mm^3
Crystal colour	colorless
Theta range for data collection	3.3 to 67.1 deg.
Index ranges	$-5 \le h \le 7$, $-18 \le k \le 21$, $-22 \le l \le 23$
Reflections collected	19691
Independent reflections	3740 (R(int) = 0.1067)
Observed reflections	1645 ($I > 2\sigma(I)$)
Absorption correction	Semi-empirical from equivalents
Max. and min. transmission	1.82 and 0.60
Refinement method	Full-matrix least-squares on F^2
Data/restraints/parameters	3740 / 1161 / 420
Goodness-of-fit on F^2	0.99
Final R indices (I>2sigma(I))	R1 = 0.072, wR2 = 0.176
Largest diff. peak and hole	0.41 and -0.41 eÅ^{-3}

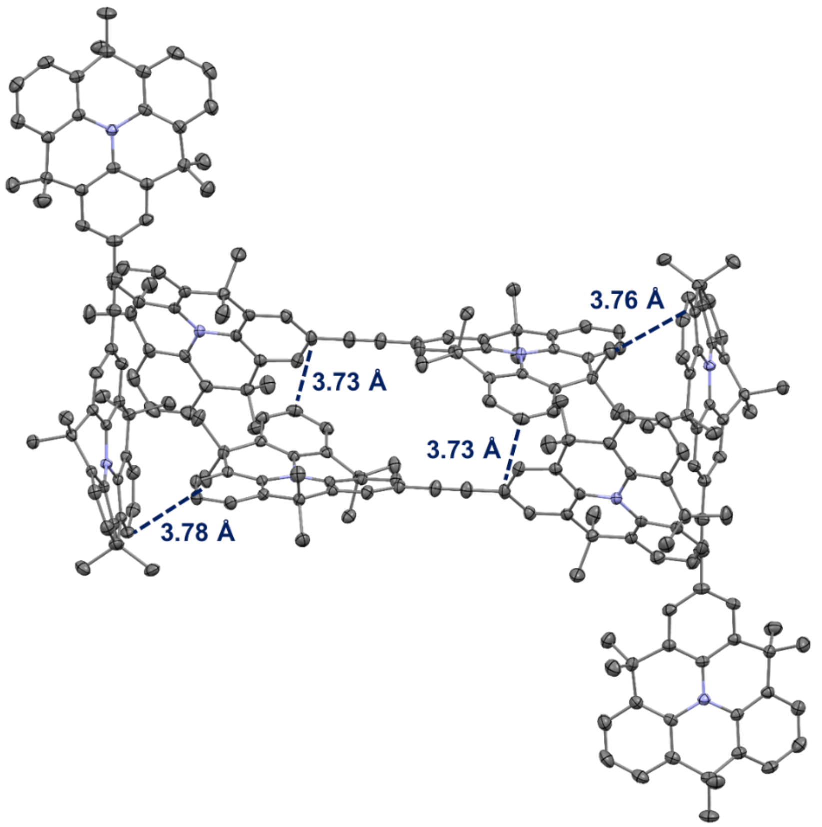

Figure S18: Crystal packing of DTA in the unit cell (thermal ellipsoids at 50% probability, color code: blue = nitrogen, grey = carbon, hydrogens are omitted for clarity).

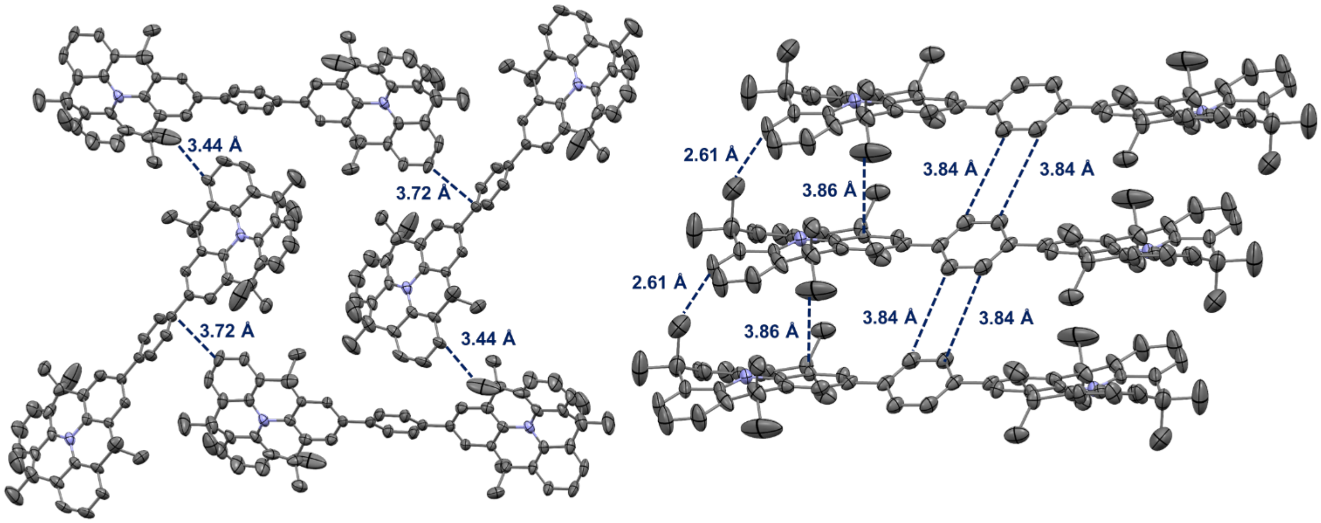

Figure S19: Crystal packing of DTB in the unit cell (left) and columnar stack observed for each DTB molecule in the unit cell (right) (thermal ellipsoids at 50% probability, color code: blue = nitrogen, grey = carbon, hydrogens are omitted for clarity).

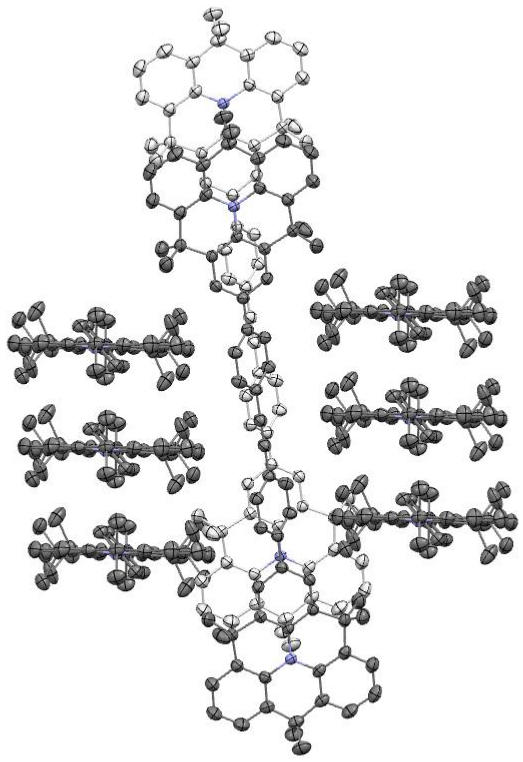

Figure S20: Crystal packing of DTT in columnar stacks (thermal ellipsoids at 50% probability, color code: blue = nitrogen, grey = carbon, hydrogens are omitted for clarity).

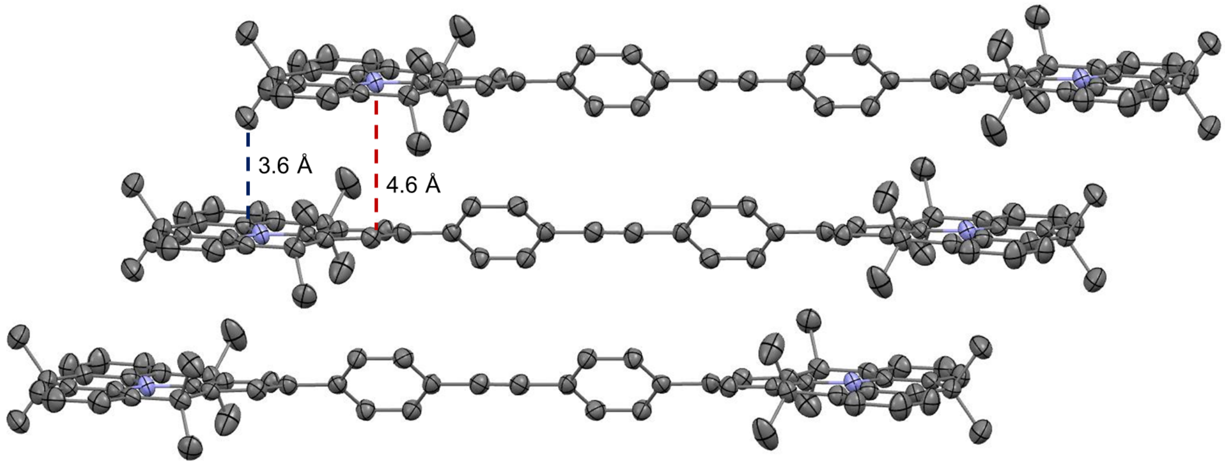

Figure S21: Crystal packing of DTT within the horizontal stacks (bottom) (thermal ellipsoids at 50% probability, color code: blue = nitrogen, grey = carbon, hydrogens are omitted for clarity).

In general, intensities for DTA were corrected for Lorentz and polarization effects, an empirical scaling and absorption correction was applied using SADABS[6] based on the Laue symmetry of the reciprocal space. For DTT intensities were corrected for Lorentz and polarization effects, an empirical scaling and absorption correction was applied using X-Area LANA 1.70.0.0 (STOE, 2017) based on the Laue symmetry of the reciprocal space. Structures solved with SHELXT-2018/2 (Sheldrick 2015)[7] and refined against F^2 with a Full-matrix least-squares algorithm using the SHELXL-2018/3 (Sheldrick, 2018) software.[8]

6 Electrochemistry

6.1 Square wave voltammetry

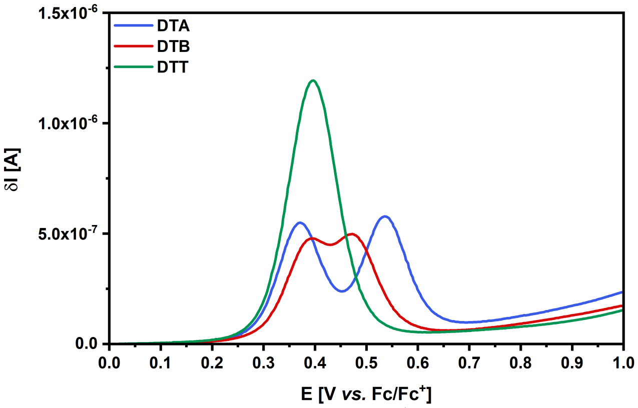

Figure S22: Square wave voltammograms of DTA, DTB, and DTT (2 x 10^{-4} M) in CH_2Cl_2 with 0.2 M $TBAPF_6$ at RT. WE: Pt; CE: Pt, Re: Ag.

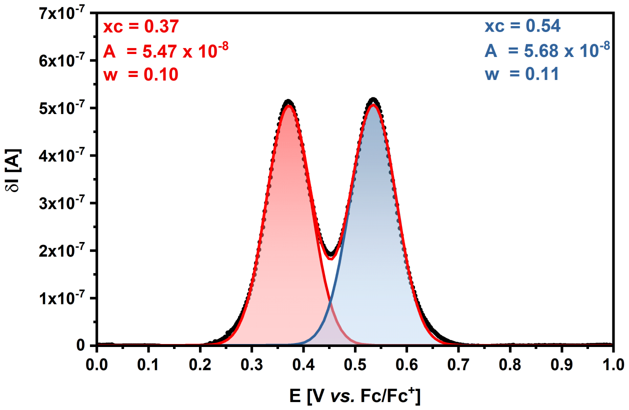

Figure S23: Peak analysis of the square wave voltammogram of DTA in CH_2Cl_2.

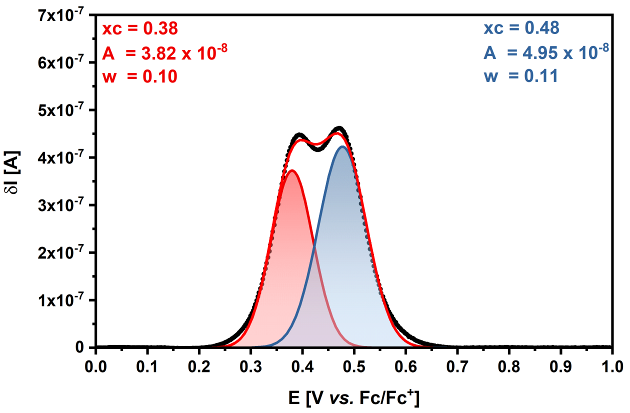

Figure S24: Peak analysis of the square wave voltammogram of DTB in CH_2Cl_2.

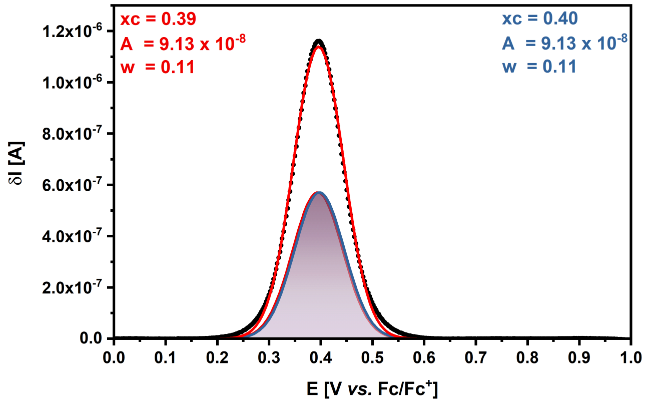

Figure S25: Peak analysis of the square wave voltammogram of DTT in CH_2Cl_2.

6.2 Cyclic voltammetry

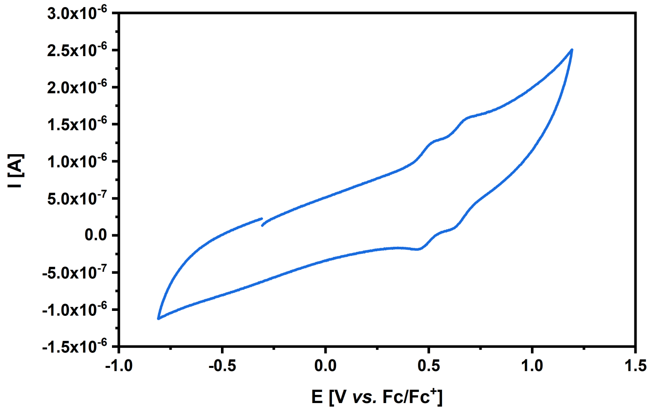

Figure S26: Cyclic voltammogram of DTA (2 x 10^{-4} M) in CH_2Cl_2 with 0.2 M $TBAPF_6$ at RT. WE: Pt; CE: Pt, Re: Ag, scan rate: 100 mV/s.

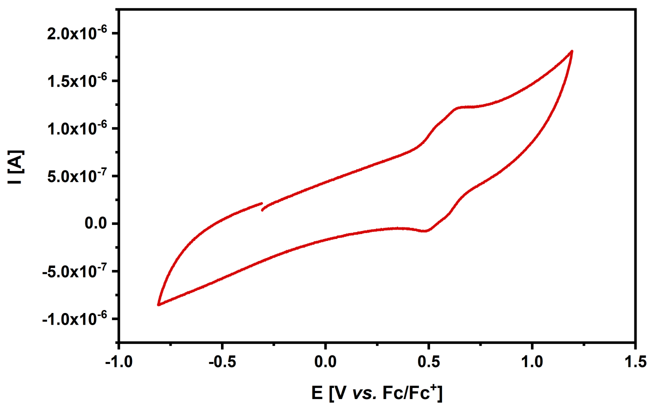

Figure S27: Cyclic voltammogram of DTB (2 x 10^{-4} M) in CH_2Cl_2 with 0.2 M $TBAPF_6$ at RT. WE: Pt; CE: Pt, Re: Ag, scan rate: 100 mV/s.

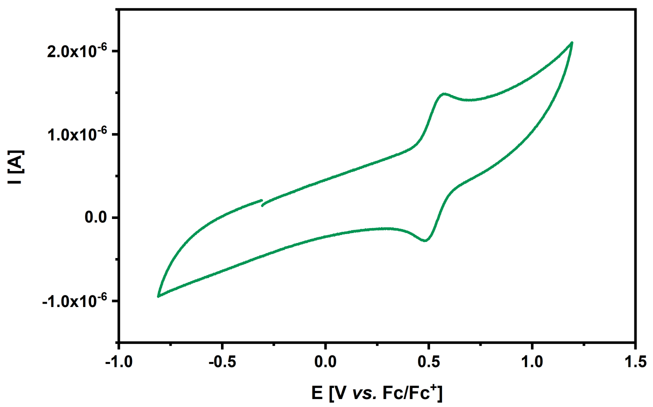

Figure S28: Cyclic voltammogram of DTT (2 x 10^{-4} M) in CH_2Cl_2 with 0.2 M $TBAPF_6$ at RT. WE: Pt; CE: Pt, Re: Ag, scan rate: 100 mV/s.

6.3 Spectroelectrochemistry

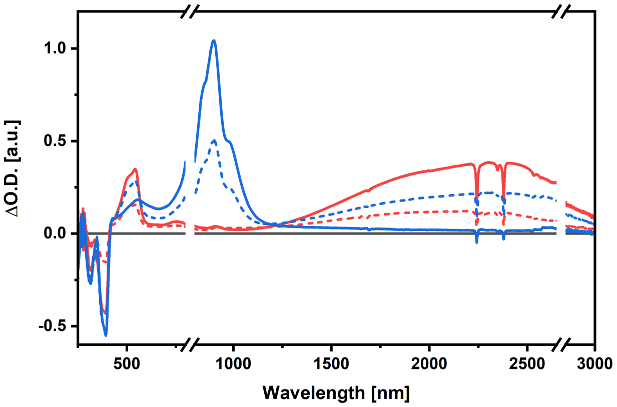

Figure S29: Absorption changes upon electrochemical oxidation of DTA in CH_2Cl_2 to its mono- (blue) and dication (red).

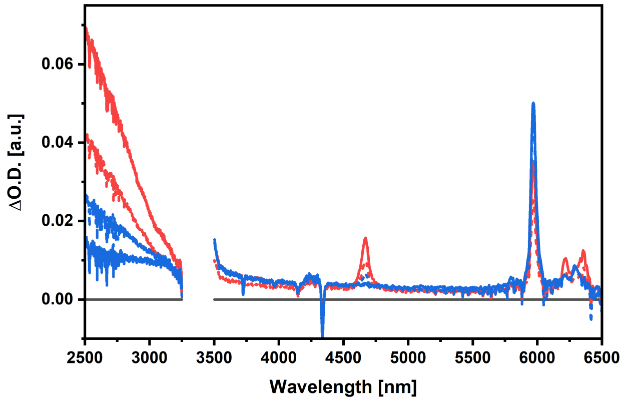

Figure S30: Absorption changes in the IR region upon electrochemical oxidation of DTA in CH_2Cl_2 to the corresponding mono- (blue) and dication (red). The tail of the IV-CT band is visible between 2500 and 3500 nm. The feature at 4671 nm in the spectrum of the monocation is due to the stretching vibration of the central C-C triple bond. The band at 5969 nm in the spectra of mono- and dication belongs to vibrations of the aromatic rings of the *N*-HTAs.

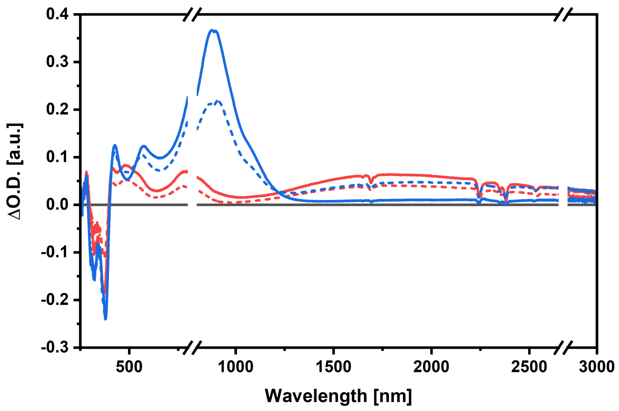

Figure S31: Absorption changes upon electrochemical oxidation of DTB in CH_2Cl_2 to its mono- (blue) and dication (red).

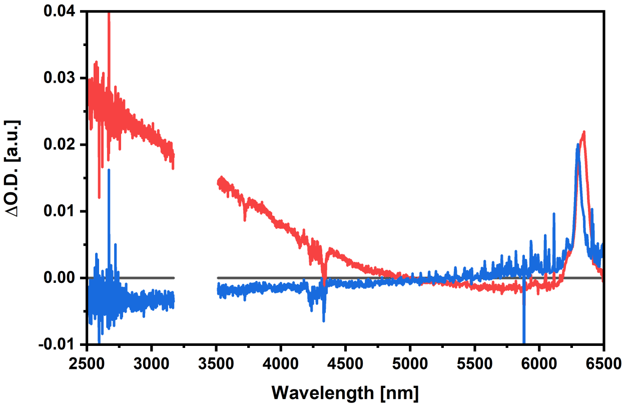

Figure S32: Absorption changes in the IR region upon electrochemical oxidation of DTB in CH_2Cl_2 to the corresponding mono- (blue) and dication (red). The tail of the IV-CT band is visible between 2500 and 5000 nm. The bands at 6356 and 6299 nm in the spectra of the mono- and dication, respectively, belong to vibrations of the aromatic rings of the *N*-HTAs.

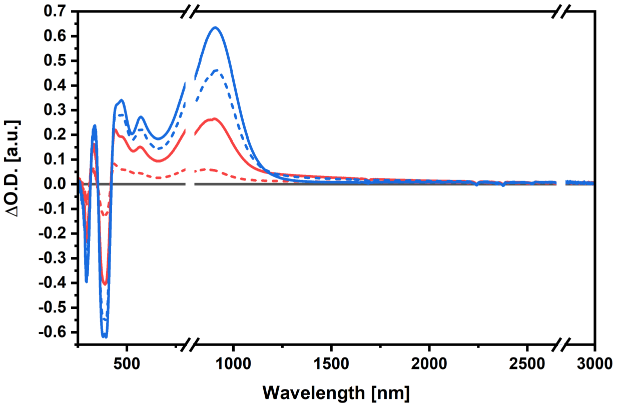

Figure S33: Absorption changes upon electrochemical oxidation of DTT in CH_2Cl_2 to its mono- (blue) and dication (red).

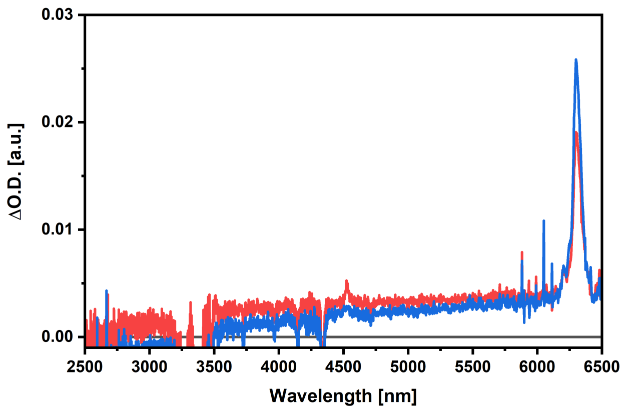

Figure S34: Absorption changes in the IR region upon electrochemical oxidation of DTT in CH_2Cl_2 to the corresponding mono- (blue) and dication (red). The feature at 4523 nm in the spectrum of the monocation is due to the stretching vibration of the central C-C triple bond. The band at 6299 nm in the spectra of the mono- and dication belongs to vibrations of the aromatic rings of the *N*-HTAs.

7 Steady-state absorption and fluorescence spectroscopy

7.1 Characterization of the neutral compounds

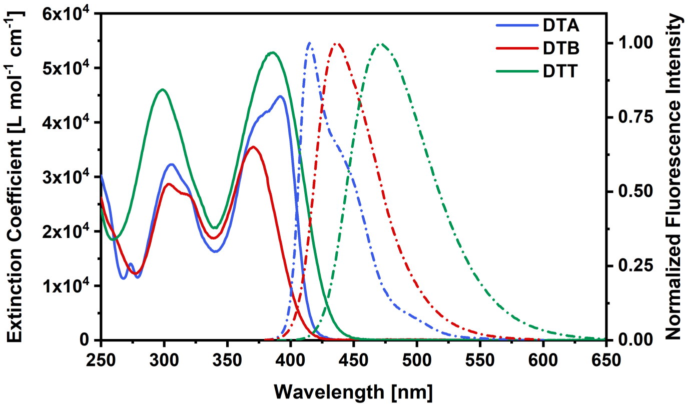

Figure S35: Steady-state absorption and fluorescence spectra of DTA (λ_{Ex} = 370 nm), DTB (λ_{Ex} = 370 nm), and DTT (λ_{Ex} = 380 nm) in CH_2Cl_2.

Table S4: Photo-physical characteristics of DTA, DTB, and DTT in CH_2Cl_2.

Compound	λ_{Abs} [nm]	ε [L mol^{-1} cm^{-1}]	λ_{Fl} [nm]	φ_{Fl}	$\tilde{\nu}_{Stokes}$ [cm^{-1}]
**DTA**	392	44 788	415	0.97[a]	1414
**DTB**	371	35 462	437	0.87[b]	4071
**DTT**	386	52 798	471	0.56[c]	4675

a) *vs.* 9,10-Diphenylanthracene in cyclohexane (0.95)[9]
b) *vs.* 2,2'-(1,4-phenylene)bis[5-phenyloxazole] (0.97)[9]
c) *vs.* Coumarin 102 in EtOH (0.58)[10]

7.2 Titrations with $SbCl_5$

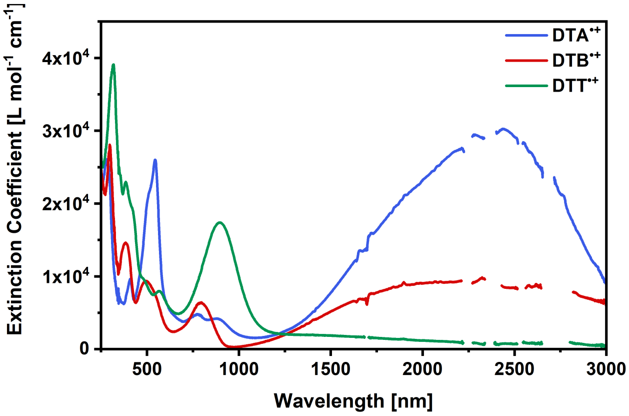

Figure S36: Absorption spectra of $DTA^{•+}$, $DTB^{•+}$, and $DTT^{•+}$ in CH_2Cl_2 generated *in-situ* through the addition of $SbCl_5$.

7.3 Mulliken-Hush analysis

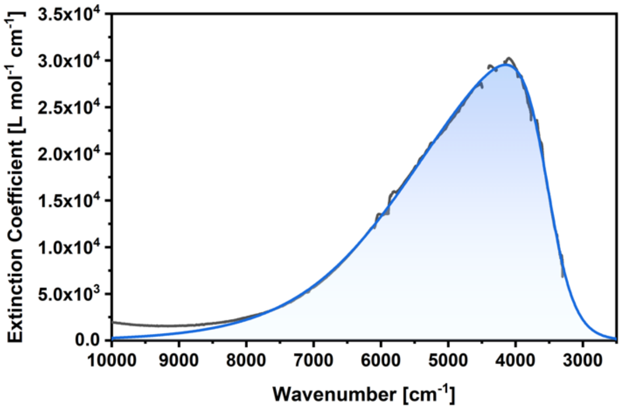

Figure S37: Band analysis for the IV-CT band of $DTA^{•+}$. An asymmetric peak function was used for the fit.

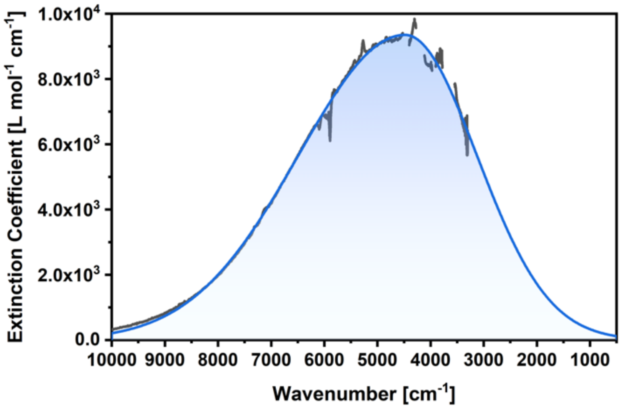

Figure S38: Band analysis for the IV-CT band of $DTB^{•+}$. An asymmetric peak function was used for the fit.

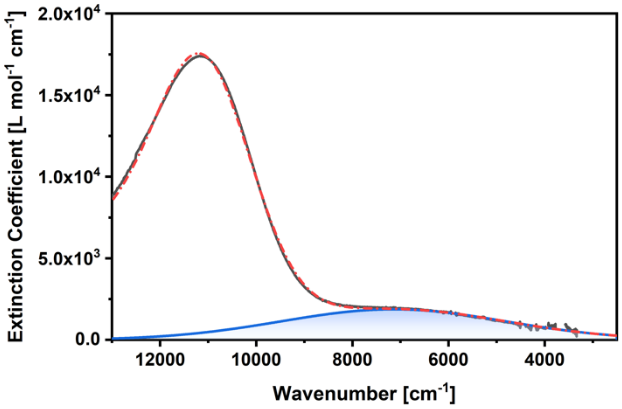

Figure S39: Band analysis for the IV-CT band of DTT$^{•+}$. The depicted part of the spectrum was fitted with Gaussian functions. The Gaussian function at the lowest transition energy was ascribed to the IV-CT.

Table S5: Characteristic electron transfer parameters obtained through Mulliken-Hush-analysis of the nIR absorption bands obtained in CH_2Cl_2.

	R [Å]	V_{MH} [cm^{-1}]	λ_{MH} [cm^{-1}]	$\Delta G^{‡}_{MH}$ [kJ mol^{-1}]	μ_{eg} [D]	$\Delta\mu_{12}$ [D]	$\Delta\mu_{eg}$ [D]
$DTA^{\bullet+}$	12.5	825	4147	4.5	13.0	65.5	60.1
$DTB^{\bullet+}$	14.3	566	4489	7.5	9.0	68.8	71.1
$DTT^{\bullet+}$	21.2	261	7147	18.4	3.7	102.2	101.9

8 Computational studies

8.1 Computational methods

All calculations were performed using the Gaussian 16[11] software package. Following the guidelines of Lambert and co-workers[12,13] we used a custom functional built from the gradient corrected BLYP[14,15] functional and HF. This exchange-correlation hybrid functional can be described by equation (S1)[12]

$$E_{xc} = (1-a)(E_x^{LSDA} + \Delta E_x^{B88}) + a\, E_x^{HF} + E_c^{LYP}. \quad \text{(S1)}$$

We used a = 0.35 in our calculations, since this particular value yielded reasonable results for organic mixed valence charge transfer systems, even if the system is close to the class II/III transition.[12] This functional is denominated as BLYP35. The basis set was def2-SVP.[16] Calculations were performed in the gas phase and in solution. Solvent effects (CH_2Cl_2, ε = 8.93) were incorporated using the C-PCM model.[17] Absorption spectra were calculated using time-dependent density functional theory (TDDFT) based on the same custom functional.

The search for the most stable conformer was achieved by performing relaxed potential energy scans along the dihedral angles. The obtained gas phase structures were proven to be an energetic minimum by performing frequency calculations (no imaginary frequencies were obtained). For DTB and DTT two conformers with equal energy were found. In accordance to the crystal structures, the results of the C_{2h}-conformer are presented herein.

The orbital interaction diagrams[18,19] were constructed from calculations on the whole molecule, the two redox sites, and the bridge, respectively. The components were generated from the frozen optimized geometry with dangling bonds saturated with hydrogens.

For the dications, only calculations of the triplet configuration are included. These exhibit lower total electronic energies in comparison to complementary calculations with the singlet open shell structure.[20]

Molecular orbitals and isosurface plots were visualized using the VESTA 3 program.[21]

8.2 Ground state properties

8.2.1 Potential energy scans

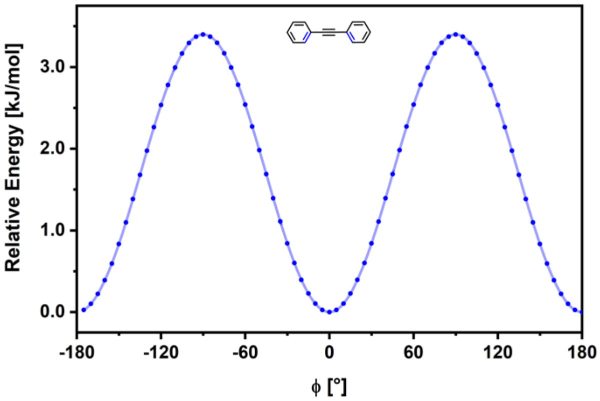

Figure S40: Potential energy scan for tolane. The scanned dihedral angle is indicated by colored bonds. Energies are relative to the most stable conformer.

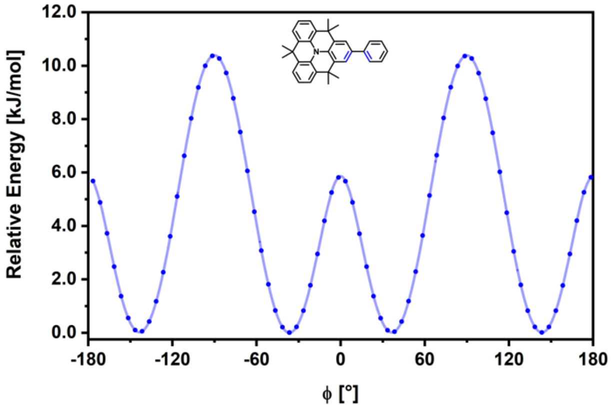

Figure S41: Potential energy scan for phenyl substituted *N*-heterotriangulene. The scanned dihedral angle is indicated by colored bonds. Energies are relative to the most stable conformer.

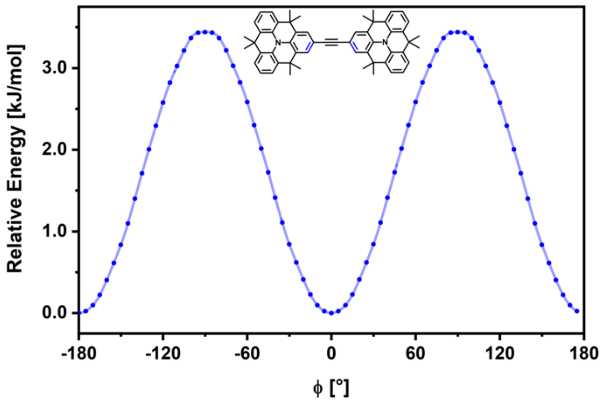

Figure S42: Potential energy scan for DTA. The scanned dihedral angle is indicated by colored bonds. Energies are relative to the most stable conformer.

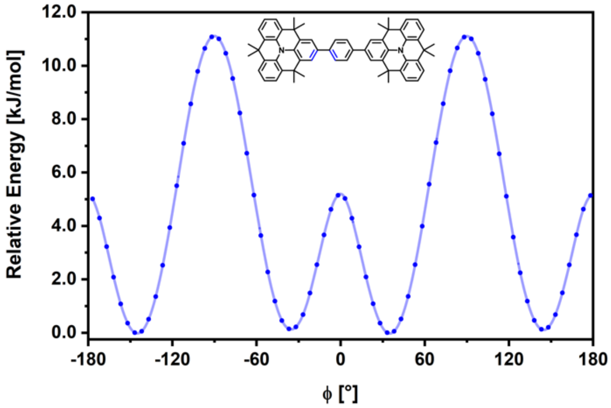

Figure S43: Potential energy scan for DTB. The scanned dihedral angle is indicated by colored bonds. Energies are relative to the most stable conformer.

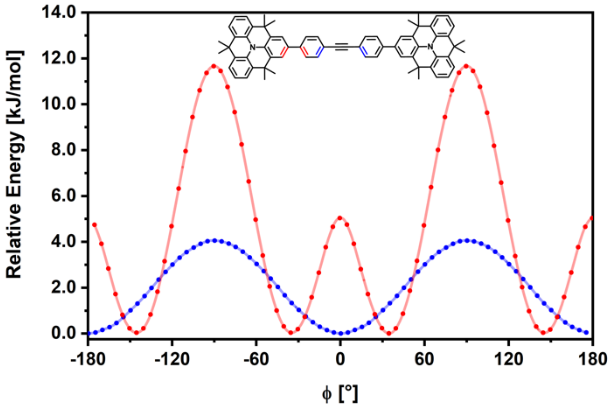

Figure S44: Potential energy scan for DTT. The scanned dihedral angles are indicated by colored bonds. Energies are relative to the most stable conformer.

8.2.2 Ground state geometries

Table S6: Coordinates of the optimized ground state geometries of DTA and its cations in CH_2Cl_2.

	DTA			$DTA^{\bullet+}$			DTA^{2+}		
	X	Y	Z	X	Y	Z	X	Y	Z
C	8.431882	-3.689061	0.004595	8.406418	-3.686310	-0.017940	8.401420	-3.674652	-0.042274
C	7.048383	-3.664222	0.005384	7.022953	-3.666198	-0.000923	7.016955	-3.659364	-0.003495
C	6.326454	-2.468649	0.005275	6.300177	-2.472267	0.004836	6.295399	-2.469692	0.008771
C	7.014411	-1.233470	0.004508	6.986777	-1.237183	-0.005335	6.991927	-1.231980	-0.014268
C	9.102033	-2.478566	0.003720	9.075243	-2.475829	-0.029306	9.080454	-2.468170	-0.068579
C	8.427910	-1.255142	0.003716	8.399334	-1.253433	-0.023011	8.411566	-1.247639	-0.054093
H	8.975807	-4.631326	0.004663	8.952187	-4.627326	-0.022549	8.944789	-4.616677	-0.052533
H	10.188434	-2.479852	0.002980	10.161169	-2.476214	-0.043672	10.165056	-2.477371	-0.100975
H	6.506271	-4.605697	0.006071	6.483686	-4.608919	0.007857	6.482753	-4.603683	0.016847
N	6.303263	-0.002160	0.004537	6.268522	-0.002144	0.002114	6.282887	-0.002129	0.002895
C	4.889619	-0.001933	0.004864	4.869935	-0.001918	0.015828	4.866889	-0.001952	0.032985
C	4.158680	-1.214474	0.005294	4.134964	-1.221133	0.022686	4.140511	-1.222834	0.046579
C	4.159084	1.210858	0.004782	4.135398	1.217572	0.023039	4.140858	1.219111	0.048896
C	2.767423	-1.189486	0.005308	2.750502	-1.195563	0.032486	2.753463	-1.196477	0.066095
C	2.035342	-0.001446	0.004938	2.018216	-0.001395	0.036564	2.031026	-0.001587	0.073863
C	2.767819	1.186352	0.004768	2.750924	1.192521	0.032894	2.753800	1.193112	0.068442
H	2.221811	-2.127863	0.005640	2.201704	-2.131659	0.037173	2.203062	-2.130644	0.075756
C	0.607513	-0.001221	0.004714	0.606466	-0.001168	0.042017	0.606806	-0.001410	0.083136
H	2.222533	2.124916	0.004629	2.202480	2.128816	0.037974	2.203664	2.127413	0.080041
C	7.014827	1.228900	0.004198	6.987218	1.232586	-0.005332	6.992272	1.227539	-0.012460
C	8.428335	1.250096	0.003558	8.399788	1.248335	-0.023222	8.411908	1.242857	-0.052593
C	6.327274	2.464314	0.004575	6.301053	2.467935	0.005018	6.296097	2.465412	0.012666
C	9.102857	2.473297	0.003421	9.076115	2.470490	-0.029787	9.081127	2.463224	-0.065715
C	7.049597	3.659646	0.004522	7.024253	3.661606	-0.000964	7.017982	3.654899	0.001829
C	8.433104	3.684012	0.003960	8.407719	3.681201	-0.018369	8.402433	3.669854	-0.037542
H	10.189259	2.474237	0.002806	10.162040	2.470510	-0.044370	10.165721	2.472172	-0.098428
H	6.507807	4.601306	0.004864	6.485344	4.604528	0.007992	6.484055	4.599333	0.023846
H	8.977357	4.626089	0.003919	8.953844	4.622013	-0.023179	8.946061	4.611741	-0.046713
C	4.811088	-2.588013	0.006019	4.787318	-2.592937	0.021858	4.790309	-2.586784	0.047474
C	4.364561	-3.359635	1.272801	4.354737	-3.357539	1.297892	4.376760	-3.337343	1.341248
H	3.275837	-3.480420	1.301772	3.266352	-3.477906	1.338113	3.289755	-3.457032	1.393837
H	4.806510	-4.361729	1.303117	4.796902	-4.359208	1.327146	4.821042	-4.337268	1.370597
H	4.671982	-2.824389	2.179641	4.672002	-2.818105	2.198616	4.706258	-2.788337	2.231126
C	4.363500	-3.361615	-1.259187	4.327499	-3.370410	-1.236784	4.313656	-3.375013	-1.201154
H	4.805356	-4.363777	-1.288299	4.768736	-4.372449	-1.265397	4.755456	-4.376091	-1.223044
H	3.274763	-3.482457	-1.287120	3.238633	-3.491462	-1.252761	3.225784	-3.496559	-1.197351
H	4.670210	-2.827804	-2.167113	4.625424	-2.840214	-2.149507	4.598587	-2.853104	-2.122117
C	4.811954	2.584176	0.004891	4.788255	2.589140	0.022707	4.791066	2.582867	0.052611
C	4.365351	3.356693	1.271105	4.356508	3.353131	1.299397	4.378713	3.330278	1.348602
H	3.276663	3.477854	1.299745	3.268180	3.473871	1.340149	3.291773	3.450119	1.402301
H	4.672378	2.821886	2.178337	4.673953	2.813030	2.199657	4.708723	2.778918	2.236836
H	4.807595	4.358668	1.300942	4.799104	4.354602	1.329048	4.823366	4.329976	1.380116
C	4.364958	3.357184	-1.260892	4.328169	3.367538	-1.235268	4.313744	3.374391	-1.193660
H	4.807215	4.359158	-1.290482	4.769713	4.369450	-1.263454	4.755665	4.375459	-1.213277
H	4.671693	2.822715	-2.168421	4.625536	2.837799	-2.148436	4.597974	2.854801	-2.116148
H	3.276269	3.478402	-1.289158	3.239347	3.489002	-1.250745	3.225909	3.496173	-1.188876
C	9.288811	-0.002665	0.002649	9.258619	-0.002699	-0.037738	9.264708	-0.002487	-0.085828
C	10.180471	-0.002876	-1.264269	10.128226	-0.002988	-1.320372	10.104028	-0.001852	-1.391509
H	10.828394	0.880258	-1.294088	10.775317	0.880028	-1.360203	10.748803	0.881439	-1.440434
H	10.828127	-0.886209	-1.294005	10.775006	-0.886238	-1.360045	10.748527	-0.885287	-1.441461

H	9.562999	-0.002827	-2.171012	9.495525	-0.002961	-2.216398	9.451811	-0.001237	-2.272759
C	10.183593	-0.002757	1.267350	10.172707	-0.002739	1.213567	10.204367	-0.003333	1.149370
H	10.831354	-0.886046	1.295610	10.820815	-0.885643	1.230869	10.851591	-0.885966	1.147882
H	10.831659	0.880312	1.295513	10.821118	0.879946	1.230674	10.851823	0.879131	1.148911
H	9.568370	-0.002603	2.175621	9.571889	-0.002537	2.131272	9.623614	-0.003797	2.079244
H	-4.668825	2.825538	-2.169777	-4.619375	2.848798	-2.139325	-4.596827	2.851064	-2.124425
H	-9.561746	0.000767	-2.176960	-9.471219	0.000045	-2.235492	-9.449973	-0.000951	-2.278840
H	-4.669542	-2.825107	-2.171714	-4.620217	-2.849443	-2.139782	-4.595819	-2.858387	-2.118105
C	-4.362677	3.359294	-1.261630	-4.324634	3.374064	-1.223119	-4.312505	3.372739	-1.203141
H	-3.273931	3.480201	-1.288922	-3.236335	3.494992	-1.232506	-3.224634	3.494268	-1.198584
C	-10.179640	0.000544	-1.270505	-10.112908	0.000105	-1.346256	-10.102796	-0.000422	-1.398040
H	-4.804579	4.361431	-1.290914	-4.766492	4.375429	-1.245267	-4.754253	4.373839	-1.225091
C	-4.363532	-3.359591	-1.263948	-4.325521	-3.374966	-1.223708	-4.312449	-3.377539	-1.195107
H	-10.827314	0.883859	-1.300299	-10.758583	0.883393	-1.390071	-10.747242	0.883033	-1.448334
H	-10.827530	-0.882593	-1.300880	-10.758782	-0.883037	-1.390097	-10.747559	-0.883693	-1.447508
H	-3.274817	-3.480770	-1.291330	-3.237251	-3.496151	-1.233212	-3.224625	-3.499348	-1.189273
H	-4.805705	-4.361587	-1.293944	-4.767629	-4.376219	-1.246020	-4.754330	-4.378622	-1.214669
C	-2.767222	1.187126	0.003686	-2.754776	1.196397	0.037266	-2.753218	1.193865	0.064526
C	-2.035088	-0.000883	0.003663	-2.022368	-0.000839	0.039698	-2.030820	-0.001044	0.072759
C	-4.158478	1.212043	0.002783	-4.135575	1.223557	0.027822	-4.140257	1.220256	0.044257
C	-2.767517	-1.188711	0.002883	-2.755049	-1.197908	0.037059	-2.753625	-1.195725	0.067045
C	-4.810951	2.585558	0.003249	-4.787760	2.589987	0.031863	-4.790022	2.584227	0.044968
C	-4.889379	-0.000529	0.001743	-4.871908	-0.000514	0.019264	-4.866654	-0.000605	0.030255
C	-4.158779	-1.213283	0.001966	-4.135854	-1.224754	0.027637	-4.140673	-1.221688	0.046693
C	-6.326313	2.466135	0.001630	-6.295878	2.470518	0.009088	-6.295089	2.467170	0.005221
H	-2.221660	2.125530	0.004475	-2.203487	2.130440	0.043532	-2.202799	2.128017	0.074469
C	-7.048290	3.661682	0.001524	-7.018870	3.661805	0.003568	-7.016609	3.656859	-0.007410
C	-0.607258	-0.001062	0.004338	-0.613955	-0.001004	0.043411	-0.606605	-0.001258	0.082800
C	-7.014233	1.230937	0.000260	-6.986410	1.234852	-0.006404	-6.991629	1.229472	-0.018413
C	-8.431790	3.686452	-0.000034	-8.402720	3.677030	-0.020029	-8.401047	3.672184	-0.047186
C	-8.427724	1.252531	-0.001358	-8.401262	1.247229	-0.030668	-8.411240	1.245172	-0.059286
C	-9.101896	2.475934	-0.001509	-9.075281	2.468316	-0.037969	-9.080089	2.465720	-0.074137
C	-9.288570	0.000017	-0.003181	-9.257214	-0.000013	-0.052832	-9.264391	0.000047	-0.091772
N	-6.303014	-0.000354	0.000509	-6.266425	-0.000355	0.002595	-6.282639	-0.000389	-0.000790
C	-7.014538	-1.231467	-0.000561	-6.986691	-1.235397	-0.006500	-6.992038	-1.230035	-0.016732
C	-4.811592	-2.586637	0.001484	-4.788347	-2.591037	0.031477	-4.790924	-2.585425	0.050334
C	-8.428034	-1.252710	-0.002184	-8.401547	-1.247450	-0.030717	-8.411642	-1.245320	-0.058036
C	-6.326924	-2.466837	-0.000025	-6.296440	-2.471221	0.008858	-6.295918	-2.467928	0.008968
C	-9.102510	-2.475946	-0.003142	-9.075844	-2.468382	-0.038049	-9.080881	-2.465667	-0.071800
H	-2.222187	-2.127250	0.003024	-2.203972	-2.132076	0.043151	-2.203516	-2.130037	0.079086
C	-7.049197	-3.662204	-0.000923	-7.019704	-3.662344	0.003294	-7.017826	-3.657393	-0.002467
C	-8.432704	-3.686630	-0.002481	-8.403558	-3.677252	-0.020216	-8.402244	-3.672316	-0.043065
H	-6.506232	4.603187	0.002676	-6.483173	4.605810	0.017385	-6.482401	4.601163	0.013432
H	-8.975765	4.628689	-0.000128	-8.948612	4.617759	-0.024930	-8.944386	4.614222	-0.057728
H	-10.188297	2.477194	-0.002841	-10.160521	2.471897	-0.058217	-10.164666	2.474947	-0.107338
H	-10.188911	-2.476935	-0.004467	-10.161086	-2.471715	-0.058250	-10.165448	-2.474580	-0.105437
H	-6.507373	-4.603844	-0.000409	-6.484221	-4.606472	0.016997	-6.483946	-4.601841	0.020076
H	-8.976912	-4.628733	-0.003199	-8.949666	-4.617854	-0.025144	-8.945885	-4.614190	-0.052730
C	-4.365192	3.357086	1.270363	-4.360738	3.345728	1.316761	-4.377344	3.334464	1.339207
C	-10.183935	-0.000287	1.261112	-10.181588	0.000073	1.192316	-10.204922	0.000766	1.142761
H	-3.276492	3.477940	1.299970	-3.272952	3.465576	1.358727	-3.290370	3.454108	1.392566
C	-4.366000	-3.359170	1.268045	-4.361367	-3.347134	1.316179	-4.379843	-3.332248	1.347074
H	-4.807222	4.359147	1.300531	-4.804396	4.346137	1.348708	-4.821631	4.334390	1.368488
H	-10.831738	0.882974	1.289312	-10.829369	0.882662	1.201627	-10.852136	0.883405	1.140913
H	-10.831965	-0.883402	1.288727	-10.829576	-0.882364	1.201596	-10.852383	-0.881694	1.141739
H	-3.277329	-3.480299	1.297558	-3.273605	-3.467236	1.358017	-3.292953	-3.452083	1.401863
H	-4.808260	-4.361151	1.297504	-4.805238	-4.347452	1.347970	-4.824585	-4.331911	1.378609

H	-4.673093	2.821724	2.176971	-4.680626	2.799977	2.212364	-4.707464	2.785249	2.228726
H	-9.569124	-0.000667	2.169661	-9.588938	-0.000012	2.114947	-9.624832	0.001119	2.073049
H	-4.673769	-2.824382	2.175036	-4.681046	-2.801493	2.211924	-4.710687	-2.780461	2.234732

Table S7: Coordinates of the optimized ground state geometries of DTB and its cations in CH_2Cl_2.

	**DTB**			**DTB$^{•+}$**			**DTB^{2+}**		
	X	Y	Z	X	Y	Z	X	Y	Z
C	1.011574	-0.641440	0.695740	1.057520	-0.565133	0.686888	1.011164	-0.676869	0.685919
C	1.011543	-0.641555	-0.694335	1.060473	-0.561552	-0.699807	1.011074	-0.677006	-0.684543
C	0.000162	0.000724	1.427520	0.000530	0.003549	1.419828	-0.011502	-0.055034	1.406433
C	-1.011294	0.642747	0.695679	-1.056441	0.572367	0.686957	-1.035701	0.564126	0.685917
C	-1.011329	0.642629	-0.694397	-1.059452	0.568852	-0.699743	-1.035792	0.563987	-0.684521
C	0.000095	0.000486	-1.426175	0.000473	0.003621	-1.431615	-0.011687	-0.055317	-1.405047
C	3.686870	0.004236	-9.314445	3.673758	-0.061083	-9.292800	3.678114	0.067232	-9.253362
C	3.661085	0.008691	-7.930876	3.658271	-0.063908	-7.908318	3.659348	0.041227	-7.868643
C	2.465254	0.007763	-7.209180	2.467635	-0.046153	-7.186232	2.467489	0.023000	-7.150058
C	1.230311	0.002480	-7.898281	1.232062	-0.023235	-7.881835	1.232437	0.028232	-7.850357
C	2.476646	0.000483	-9.985459	2.466874	-0.040885	-9.970228	2.473133	0.076464	-9.935649
C	1.252952	0.000373	-9.312055	1.245960	-0.022293	-9.299409	1.250766	0.057395	-9.269410
H	4.629502	0.004612	-9.857786	4.615349	-0.075370	-9.836961	4.621468	0.081505	-9.794396
H	2.478615	-0.001746	-11.071920	2.474229	-0.039973	-11.055479	2.484653	0.099856	-11.020514
H	4.602379	0.013803	-7.388383	4.602179	-0.081105	-7.372954	4.602302	0.036203	-7.331538
N	0.000033	0.000248	-7.188896	-0.000001	0.000313	-7.167484	0.000540	0.004940	-7.142998
C	0.000048	0.000278	-5.772394	0.000122	0.001685	-5.763022	-0.002572	-0.017991	-5.730491
C	1.209750	0.001934	-5.041083	1.219174	-0.032353	-5.028818	1.213949	-0.020759	-4.997860
C	-1.209638	-0.001319	-5.041057	-1.218795	0.037087	-5.028660	-1.222356	-0.037645	-5.003416
C	1.180594	0.000547	-3.647544	1.186801	-0.036573	-3.643994	1.178938	-0.038370	-3.611655
C	0.000078	0.000396	-2.908814	0.000355	0.003326	-2.904499	-0.008858	-0.048359	-2.882278
C	-1.180453	0.000185	-3.647519	-1.186198	0.042821	-3.643841	-1.193473	-0.048787	-3.617000
H	2.125937	0.024014	-3.114369	2.130284	-0.042520	-3.110540	2.119629	-0.017165	-3.072843
H	-2.125785	-0.023196	-3.114321	-2.129595	0.049662	-3.110244	-2.136317	-0.085685	-3.082752
C	-1.230259	-0.001991	-7.898256	-1.232188	0.022466	-7.881663	-1.228233	0.006023	-7.856096
C	-1.252929	0.000056	-9.312029	-1.246356	0.017730	-9.299227	-1.240510	0.038842	-9.275137
C	-2.465188	-0.007178	-7.209128	-2.467615	0.047866	-7.185888	-2.466162	-0.026924	-7.161639
C	-2.476638	-0.000011	-9.985407	-2.467385	0.035352	-9.969864	-2.459946	0.039829	-9.946991
C	-3.661034	-0.008060	-7.930798	-3.658368	0.064946	-7.907798	-3.654769	-0.029068	-7.885789
C	-3.686848	-0.003661	-9.314367	-3.674120	0.058505	-9.292265	-3.667664	0.005145	-9.270402
H	-2.478629	0.002174	-11.071868	-2.474949	0.031304	-11.055106	-2.466876	0.067429	-11.031797
H	-4.602317	-0.013080	-7.388286	-4.602159	0.084377	-7.372305	-4.599851	-0.057697	-7.353243
H	-4.629491	-0.003995	-9.857688	-4.615801	0.072173	-9.836285	-4.608621	0.004712	-9.815779
C	2.583394	0.017575	-5.693218	2.584009	-0.054343	-5.679127	2.579715	0.003041	-5.643602
C	3.338602	1.297041	-5.254286	3.377822	1.200419	-5.229227	3.335505	1.276730	-5.180402
H	3.460650	1.333188	-4.165857	3.498918	1.219000	-4.141314	3.455275	1.287832	-4.092288
H	4.339358	1.338796	-5.698655	4.378844	1.209853	-5.672160	4.335914	1.319494	-5.622468
H	2.790507	2.194963	-5.565355	2.859120	2.117340	-5.532874	2.790369	2.180846	-5.475876
C	3.374612	-1.234058	-5.238146	3.330372	-1.341182	-5.239178	3.361794	-1.265755	-5.212445
H	4.378101	-1.251711	-5.677625	4.330658	-1.384336	-5.681717	4.364607	-1.275165	-5.651069
H	3.494092	-1.254670	-4.149161	3.449460	-1.372293	-4.151280	3.477875	-1.303211	-4.124506
H	2.854234	-2.151101	-5.540885	2.778169	-2.235807	-5.550257	2.837335	-2.173183	-5.534006
C	-2.583296	-0.016904	-5.693163	-2.583713	0.059497	-5.678782	-2.584825	-0.065571	-5.655979
C	-3.374404	1.234814	-5.238136	-3.328047	1.348425	-5.241509	-3.399148	1.166934	-5.181855
H	-3.493845	1.255493	-4.149148	-3.446863	1.382041	-4.153649	-3.520979	1.162272	-4.094054
H	-2.853965	2.151801	-5.540941	-2.774526	2.241533	-5.554605	-2.896009	2.098041	-5.467693
H	-4.377905	1.252519	-5.677588	-4.328328	1.392125	-5.684028	-4.400560	1.168257	-5.623504
C	-3.338597	-1.296289	-5.254152	-3.379352	-1.193080	-5.226032	-3.311369	-1.373626	-5.244095
H	-4.339360	-1.337995	-5.698510	-4.380508	-1.201916	-5.668654	-4.308923	-1.423137	-5.692160
H	-2.790571	-2.194270	-5.565175	-2.862143	-2.111435	-5.527875	-2.743689	-2.252727	-5.571036
H	-3.460644	-1.332368	-4.165721	-3.500259	-1.209222	-4.138066	-3.433264	-1.427876	-4.157376
C	0.000002	0.000165	-10.172896	-0.000291	-0.004159	-10.154026	0.006888	0.075034	-10.125808
C	-0.000249	-1.265486	-11.066282	-0.020109	-1.276891	-11.040928	0.017829	-1.165865	-11.057447

H	-0.883459	-1.294221	-11.714209	-0.903001	-1.290062	-11.688084	-0.863220	-1.175638	-11.706846
H	0.883056	-1.294720	-11.714061	0.862747	-1.318088	-11.686975	0.901847	-1.163112	-11.702860
H	-0.000565	-2.173083	-10.450039	-0.034855	-2.181284	-10.421187	0.023071	-2.091976	-10.470663
C	0.000236	1.265710	-11.066430	0.019330	1.264380	-11.046895	-0.000401	1.375046	-10.973407
H	0.883431	1.294371	-11.714380	0.901993	1.274523	-11.694398	0.884081	1.427020	-11.616384
H	-0.883080	1.294869	-11.714198	-0.863625	1.302606	-11.692969	-0.882568	1.414532	-11.620434
H	0.000564	2.173380	-10.450295	0.034256	2.171676	-10.431422	-0.008148	2.260373	-10.326715
C	-3.686457	-0.001022	9.315900	-3.685016	-0.089190	9.301279	-3.666439	0.007095	9.272259
C	-2.476216	0.003139	9.986887	-2.475195	-0.058067	9.971905	-2.458630	0.041857	9.948682
C	-1.252539	0.003021	9.313468	-1.252025	-0.028703	9.298027	-1.239283	0.040714	9.276667
C	-1.229927	0.000245	7.899700	-1.230858	-0.028922	7.884669	-1.227195	0.007639	7.857631
C	-3.660703	-0.006121	7.932335	-3.660359	-0.091017	7.917718	-3.653728	-0.027363	7.887651
C	-2.464879	-0.005420	7.210614	-2.465144	-0.061356	7.195974	-2.465216	-0.025393	7.163343
H	-4.629076	-0.001176	9.859263	-4.627019	-0.112197	9.845121	-4.607324	0.006791	9.817760
H	-4.602003	-0.011573	7.389859	-4.601658	-0.116815	7.375943	-4.598881	-0.056050	7.355234
H	-2.478158	0.005898	11.073346	-2.476968	-0.057120	11.058263	-2.465416	0.069645	11.033485
N	0.000336	0.002217	7.190241	0.000321	0.001588	7.173412	0.001484	0.006387	7.144371
C	1.230633	0.004681	7.899612	1.231446	0.031768	7.884787	1.233475	0.029798	7.851562
C	1.253354	0.003353	9.313386	1.252502	0.030437	9.298144	1.251992	0.059233	9.270607
C	2.465560	0.009400	7.210465	2.465770	0.064959	7.196200	2.468435	0.024414	7.151102
C	2.477068	0.003689	9.986730	2.475619	0.059512	9.972133	2.474447	0.078426	9.936681
C	3.687276	0.006884	9.315673	3.685479	0.091421	9.301616	3.679338	0.069059	9.254237
C	3.661425	0.010530	7.932103	3.660927	0.094323	7.918056	3.660389	0.042778	7.869526
H	2.479066	0.002095	11.073191	2.477318	0.057649	11.058490	2.486111	0.102022	11.021541
H	4.629926	0.007467	9.858978	4.627440	0.114141	9.845544	4.622764	0.083435	9.795143
H	4.602686	0.015134	7.389549	4.602264	0.120703	7.376374	4.603272	0.037638	7.332297
C	0.000297	0.001640	5.773744	0.000368	0.002214	5.761509	-0.001814	-0.016825	5.731869
C	-1.209429	-0.000183	5.042460	-1.210317	-0.023968	5.028886	-1.221695	-0.036585	5.004959
C	1.209976	0.002938	5.042385	1.211106	0.029024	5.028996	1.214610	-0.019778	4.999078
C	-1.180296	0.000924	3.648912	-1.181074	-0.018694	3.637054	-1.192995	-0.048013	3.618542
C	1.180756	0.001149	3.648842	1.181966	0.024780	3.637159	1.179415	-0.037676	3.612882
C	0.000209	0.000921	2.910169	0.000475	0.003269	2.898102	-0.008477	-0.047777	2.883662
H	-2.125641	-0.022675	3.115751	-2.126507	-0.060821	3.105876	-2.135910	-0.084980	3.084427
H	2.126077	0.024278	3.115618	2.127438	0.067284	3.106080	2.120037	-0.016609	3.073943
C	0.000435	0.003783	10.174278	0.000212	0.000275	10.158599	0.008228	0.077013	10.127168
C	0.000228	-1.261195	11.068621	0.029771	-1.265965	11.050765	0.019277	-1.163759	11.058976
H	0.883556	-1.289944	11.716388	0.913339	-1.274664	11.698647	0.903384	-1.160929	11.704267
H	-0.882944	-1.289451	11.716618	-0.852511	-1.315945	11.698537	-0.861685	-1.173428	11.708495
H	-0.000113	-2.169257	10.453062	0.050992	-2.172783	10.433786	0.024425	-2.089952	10.472320
C	0.000698	1.269996	11.066860	-0.029398	1.265206	11.052608	0.001067	1.377139	10.974590
H	-0.882584	1.299624	11.714655	-0.912980	1.272973	11.700473	-0.881012	1.416724	11.621730
H	0.883937	1.299160	11.714731	0.852823	1.314253	11.700525	0.885634	1.429191	11.617444
H	0.000972	2.177205	10.450045	-0.050583	2.172925	10.436955	-0.006756	2.262379	10.327780
C	2.583643	0.018295	5.694479	2.584047	0.074908	5.680697	2.580461	0.004123	5.644635
C	3.374167	-1.234041	5.240108	3.401370	-1.160729	5.228081	3.362443	-1.264795	5.213660
H	4.377632	-1.252019	5.679644	4.404854	-1.155780	5.667645	4.365318	-1.274130	5.652144
H	2.853260	-2.150621	5.543338	2.900694	-2.087940	5.532843	2.838004	-2.172133	5.535506
H	3.493633	-1.255297	4.151132	3.521673	-1.181146	4.139106	3.478367	-1.302505	4.125712
C	3.339561	1.297078	5.254800	3.311040	1.369862	5.239627	3.336230	1.277685	5.181050
H	3.461679	1.332528	4.166362	3.432788	1.406754	4.151136	3.455869	1.288532	4.092920
H	2.791957	2.195488	5.565326	2.743563	2.256148	5.549124	2.791157	2.181884	5.476383
H	4.340338	1.338561	5.699143	4.310461	1.433928	5.683979	4.336696	1.320522	5.622983
C	-2.583046	-0.015878	5.694645	-2.583304	-0.070375	5.680455	-2.584080	-0.064306	5.657705
C	-3.374641	1.235331	5.239084	-3.400728	1.165427	5.228488	-3.398380	1.168170	5.183466
H	-3.494178	1.255470	4.150100	-3.520919	1.186489	4.139515	-3.520362	1.163310	4.095682
H	-4.378127	1.252880	5.678572	-4.404269	1.160097	5.667914	-4.399729	1.169649	5.625257
H	-2.854525	2.152653	5.541428	-2.900204	2.092517	5.533864	-2.895133	2.099295	5.469057

C	-3.337916	-1.295742	5.256263	-3.310121	-1.365160	5.238580	-3.310770	-1.372384	5.246154
H	-4.338640	-1.337594	5.700705	-4.309550	-1.429603	5.682866	-4.308267	-1.421747	5.694362
H	-3.459985	-1.332355	4.167851	-3.431804	-1.401416	4.150059	-3.432818	-1.426822	4.159462
H	-2.789553	-2.193380	5.567675	-2.742550	-2.251560	5.547577	-2.743106	-2.251465	5.573176
H	-1.799314	1.179720	-1.218600	-1.885883	1.048793	-1.218464	-1.833506	1.085766	-1.207213
H	-1.799253	1.179933	1.219824	-1.879550	1.053947	1.209459	-1.833346	1.086012	1.208609
H	1.799505	-1.178730	-1.218486	1.886857	-1.041514	-1.218582	1.809052	-1.198353	-1.207296
H	1.799564	-1.178534	1.219932	1.880632	-1.046774	1.209328	1.809211	-1.198113	1.208669

Table S8: Coordinates of the optimized ground state geometries of DTT and its cations in CH_2Cl_2.

	DTT			$DTT^{\bullet+}$			DTT^{2+}		
	X	Y	Z	X	Y	Z	X	Y	Z
C	-12.761161	3.682275	-0.065620	-12.734918	3.694635	-0.149348	-12.707137	3.686640	-0.124836
C	-11.377599	3.656595	-0.069189	-11.351552	3.665325	-0.131329	-11.322978	3.665119	-0.087777
C	-10.655991	2.460943	-0.048059	-10.633872	2.467958	-0.087505	-10.607067	2.471702	-0.053901
C	-11.345038	1.226370	-0.022530	-11.326745	1.235701	-0.059843	-11.309578	1.238501	-0.054757
C	-13.432135	2.472321	-0.041764	-13.409618	2.486828	-0.124280	-13.391412	2.483131	-0.129382
C	-12.758683	1.248788	-0.021026	-12.740062	1.261728	-0.080817	-12.727732	1.259406	-0.095159
H	-13.304550	4.624727	-0.081931	-13.275352	4.638296	-0.183304	-13.246123	4.630917	-0.151104
H	-14.518577	2.474314	-0.039823	-14.495912	2.491797	-0.139808	-14.476070	2.496396	-0.161160
H	-10.835166	4.597698	-0.089747	-10.806306	4.604773	-0.152805	-10.783707	4.606872	-0.086177
N	-10.635281	-0.004090	0.000050	-10.620648	0.003260	-0.013093	-10.604052	0.004500	-0.016034
C	-9.219883	-0.004200	0.000039	-9.206477	-0.000871	0.007030	-9.194598	-0.001732	0.018395
C	-8.488295	1.205581	-0.020673	-8.470748	1.206749	-0.017284	-8.457600	1.213893	0.018401
C	-8.488482	-1.214094	0.020741	-8.478792	-1.212804	0.051813	-8.469174	-1.223898	0.052773
C	-7.095152	1.176452	-0.017730	-7.078158	1.173600	0.003788	-7.071486	1.175882	0.048235
C	-6.356910	-0.004421	0.000027	-6.343805	-0.009378	0.043216	-6.341957	-0.014507	0.072383
C	-7.095335	-1.185180	0.017787	-7.085945	-1.187946	0.065697	-7.082642	-1.198314	0.074610
H	-6.562507	2.121507	-0.055881	-6.542730	2.116839	-0.038283	-6.531604	2.115632	0.023745
H	-6.562836	-2.130318	0.055934	-6.557299	-2.134279	0.121929	-6.552324	-2.142595	0.123329
C	-11.345228	-1.234441	0.022640	-11.334988	-1.225057	0.012629	-11.321455	-1.223244	-0.013781
C	-12.758877	-1.256639	0.021168	-12.748372	-1.242961	-0.011413	-12.739622	-1.231909	-0.057769
C	-10.656372	-2.469121	0.048150	-10.650437	-2.461190	0.063922	-10.630953	-2.462295	0.033952
C	-13.432517	-2.480068	0.041919	-13.426031	-2.464131	0.013009	-13.414943	-2.449735	-0.055199
C	-11.378164	-3.664661	0.069291	-11.376064	-3.654309	0.088447	-11.358319	-3.649219	0.039013
C	-12.761730	-3.690127	0.065753	-12.759453	-3.675672	0.062742	-12.742372	-3.658836	-0.006320
H	-14.518960	-2.481892	0.040006	-14.512265	-2.462895	-0.006952	-14.499542	-2.453703	-0.091213
H	-10.835877	-4.605848	0.089833	-10.837177	-4.596761	0.129530	-10.828313	-4.595334	0.079075
H	-13.305265	-4.632494	0.082074	-13.306139	-4.616147	0.081812	-13.290354	-4.598284	-0.003377
C	-9.140115	2.578897	-0.058902	-9.117727	2.581547	-0.077410	-9.100404	2.581048	-0.022167
C	-8.685850	3.390159	1.180115	-8.678941	3.402330	1.160902	-8.678444	3.372811	1.243614
H	-7.596860	3.509894	1.199816	-7.589937	3.518942	1.195420	-7.590564	3.486220	1.290012
H	-9.125101	4.393882	1.180929	-9.115037	4.407304	1.146380	-9.114271	4.376918	1.240541
H	-8.989524	2.884862	2.105224	-8.997677	2.906217	2.085901	-9.009561	2.856985	2.152568
C	-8.700280	3.312913	-1.350314	-8.657153	3.302655	-1.368853	-8.624790	3.325177	-1.298060
H	-9.144558	4.312833	-1.408696	-9.097680	4.303164	-1.442606	-9.064464	4.326055	-1.353182
H	-7.611829	3.434446	-1.387773	-7.567942	3.421120	-1.391704	-7.536370	3.442723	-1.300759
H	-9.010782	2.750178	-2.239314	-8.956396	2.732815	-2.257176	-8.913448	2.772934	-2.200086
C	-9.140514	-2.587309	0.058971	-9.135364	-2.583518	0.099984	-9.125812	-2.584671	0.086364
C	-8.700775	-3.321402	1.350372	-8.717856	-3.299282	1.408913	-8.727218	-3.301319	1.403904
H	-7.612342	-3.443107	1.387814	-7.630419	-3.423175	1.465125	-7.641306	-3.425366	1.468761
H	-9.011174	-2.758622	2.239379	-9.040526	-2.722489	2.284459	-9.060317	-2.725478	2.275376
H	-9.145210	-4.321251	1.408755	-9.166098	-4.296936	1.475109	-9.178256	-4.297340	1.457818
C	-8.686390	-3.398633	-1.180058	-8.664712	-3.414454	-1.119715	-8.643364	-3.410510	-1.135077
H	-9.125794	-4.402289	-1.180872	-9.107000	-4.416752	-1.112263	-9.087342	-4.410907	-1.131683
H	-8.989998	-2.893284	-2.105159	-8.952683	-2.922169	-2.056770	-8.920132	-2.914555	-2.072768
H	-7.597418	-3.518535	-1.199774	-7.575965	-3.537974	-1.121085	-7.555929	-3.534828	-1.120378
C	-13.619566	-0.003859	0.000088	-13.604793	0.011790	-0.062121	-13.587209	0.017330	-0.108862
C	-14.512974	-0.024677	-1.265386	-14.474086	-0.020341	-1.344050	-14.426981	0.002211	-1.413695
H	-15.160947	-0.908187	-1.279741	-15.124130	-0.902278	-1.361077	-15.076150	-0.878518	-1.450532
H	-15.160712	0.858055	-1.308908	-15.118260	0.863664	-1.409240	-15.067420	0.887854	-1.476559
H	-13.896762	-0.039725	-2.172870	-13.840860	-0.046853	-2.239480	-13.774970	-0.014016	-2.295015
C	-14.512918	0.017097	1.265599	-14.521779	0.048915	1.186000	-14.526254	0.039893	1.126151
H	-15.160751	0.900710	1.279982	-15.167412	0.934165	1.178505	-15.169563	0.925398	1.112896
H	-15.160792	-0.865534	1.309146	-15.172944	-0.831361	1.226864	-15.177752	-0.839623	1.138882

H	-13.896667	0.032046	2.173058	-13.922787	0.072170	2.104769	-13.945017	0.050881	2.055728
C	-4.875221	-0.004538	0.000031	-4.862915	-0.013639	0.058433	-4.864961	-0.020869	0.091975
C	-4.146321	1.023425	0.622209	-4.137075	1.026518	0.664914	-4.142964	1.026054	0.691420
C	-4.146480	-1.032619	-0.622138	-4.131500	-1.057429	-0.534960	-4.138023	-1.072901	-0.492366
C	-2.758482	1.027947	0.624174	-2.749817	1.028748	0.677910	-2.756563	1.024182	0.709071
C	-2.758642	-1.037371	-0.624078	-2.744281	-1.065063	-0.525610	-2.751595	-1.078368	-0.485459
C	-2.035517	-0.004773	0.000056	-2.025163	-0.019188	0.081286	-2.034360	-0.028321	0.116954
C	-0.607232	-0.004899	0.000082	-0.598259	-0.020473	0.088729	-0.607696	-0.029576	0.123247
C	12.761865	3.679801	-0.065687	12.725064	3.675970	-0.074265	12.707942	3.679003	-0.129027
C	11.378298	3.654389	-0.069209	11.340946	3.656848	-0.039181	11.323792	3.658300	-0.091169
C	10.656460	2.458877	-0.048070	10.622491	2.464380	-0.019866	10.607186	2.465303	-0.057252
C	11.345269	1.224170	-0.022577	11.322035	1.230141	-0.033713	11.308963	1.231685	-0.058874
C	13.432606	2.469717	-0.041861	13.406384	2.471025	-0.090833	13.391498	2.475092	-0.134337
C	12.758918	1.246314	-0.021107	12.739785	1.248267	-0.070793	12.727103	1.251753	-0.100092
H	13.305437	4.622148	-0.082008	13.266169	4.619294	-0.089321	13.247470	4.622970	-0.155324
H	14.519048	2.471498	-0.039953	14.491156	2.481958	-0.120517	14.476144	2.487713	-0.166746
H	10.836047	4.595597	-0.089733	10.803354	4.599514	-0.027286	10.785088	4.600375	-0.088961
N	10.635273	-0.006153	0.000000	10.612765	-0.003388	-0.011497	10.602720	-0.001915	-0.020146
C	9.219875	-0.005989	0.000002	9.205683	-0.007184	0.017618	9.193276	-0.007316	0.014975
C	8.488521	1.203934	-0.020667	8.469911	1.210144	0.031744	8.457008	1.208752	0.015667
C	8.488241	-1.215742	0.020676	8.476495	-1.228533	0.032656	8.467138	-1.229054	0.049326
C	7.095373	1.175074	-0.017712	7.084456	1.174365	0.056802	7.070886	1.171556	0.046039
C	6.356902	-0.005657	0.000008	6.350834	-0.014870	0.060564	6.340647	-0.018401	0.070123
C	7.095099	-1.186559	0.017725	7.090757	-1.200215	0.048477	7.080628	-1.202650	0.071738
H	6.562911	2.120233	-0.055822	6.547102	2.115680	0.044261	6.531568	2.111641	0.022067
H	6.562418	-2.131595	0.055842	6.558963	-2.144209	0.081938	6.549777	-2.146632	0.120421
C	11.344983	-1.236641	0.022581	11.328923	-1.233082	-0.020383	11.319380	-1.230075	-0.018673
C	12.758627	-1.259113	0.021105	12.746729	-1.243690	-0.058183	12.737523	-1.239577	-0.063503
C	10.655888	-2.471188	0.048085	10.636351	-2.470954	0.010164	10.628170	-2.468737	0.029081
C	13.432031	-2.482672	0.041868	13.420164	-2.462844	-0.064773	13.412114	-2.457803	-0.061731
C	11.377449	-3.666867	0.069237	11.361542	-3.659474	0.006316	11.354835	-3.656090	0.033386
C	12.761010	-3.692601	0.065712	12.745669	-3.671277	-0.031692	12.738855	-3.666519	-0.012792
H	14.518474	-2.484706	0.039954	14.504938	-2.468042	-0.095894	14.496689	-2.462405	-0.098414
H	10.834980	-4.607950	0.089774	10.829344	-4.604869	0.032977	10.824295	-4.601903	0.073510
H	13.304363	-4.635073	0.082042	13.292060	-4.611661	-0.035810	13.286278	-4.606294	-0.010459
C	9.140606	2.577125	-0.058847	9.115841	2.576563	0.012658	9.100611	2.575539	-0.024589
C	8.700859	3.311303	-1.350197	8.642262	3.342136	-1.251113	8.624681	3.320682	-1.299762
H	7.612430	3.433054	-1.387602	7.554040	3.461329	-1.252944	7.536325	3.438824	-1.301781
H	9.145329	4.311139	-1.408541	9.083114	4.343290	-1.289765	9.064850	4.321363	-1.354527
H	9.011211	2.748559	-2.239242	8.930777	2.804228	-2.161805	8.912532	2.768832	-2.202285
C	8.686550	3.388402	1.180237	8.695194	3.348859	1.290786	8.679870	3.366846	1.241896
H	9.126001	4.392038	1.181093	9.134177	4.351540	1.304454	9.116382	4.370656	1.239171
H	7.597584	3.508349	1.199987	7.607537	3.464462	1.338038	7.592087	3.480939	1.288929
H	8.990161	2.882990	2.105304	9.023968	2.817289	2.191517	9.011135	2.850263	2.150366
C	9.140007	-2.589084	0.058864	9.130485	-2.591125	0.051320	9.122986	-2.590224	0.082319
C	8.685761	-3.400265	-1.180213	8.654593	-3.398419	-1.184932	8.639343	-3.415603	-1.138951
H	7.596767	-3.519955	-1.199964	7.566815	-3.520752	-1.178988	7.551849	-3.539316	-1.123628
H	8.989493	-2.894932	-2.105284	8.938234	-2.889494	-2.113602	8.915834	-2.919674	-2.076738
H	9.124976	-4.404005	-1.181061	9.096888	-4.399585	-1.193187	9.082738	-4.416259	-1.135980
C	8.700091	-3.323150	1.350220	8.721682	-3.325794	1.355610	8.724727	-3.306830	1.399996
H	9.144329	-4.323088	1.408572	9.170328	-4.323432	1.398585	9.175198	-4.303131	1.453483
H	9.010573	-2.760470	2.239261	9.049872	-2.763062	2.237460	9.058694	-2.731323	2.271357
H	7.611634	-3.444648	1.387627	7.635143	-3.448661	1.411411	7.638776	-3.430211	1.465467
C	13.619559	-0.006500	-0.000013	13.597102	0.004513	-0.094645	13.585824	0.009171	-0.114649
C	14.512960	0.014296	1.265466	14.533067	0.013286	1.142711	14.525508	0.030763	1.119907
H	15.160664	-0.868460	1.309001	15.183153	-0.867374	1.148650	15.176461	-0.849163	1.132021
H	15.160966	0.897781	1.279817	15.177932	0.897790	1.140030	15.169350	0.915880	1.106611

H	13.896743	0.029376	2.172946	13.949438	0.016096	2.070874	13.944741	0.041805	2.049777
C	14.512917	-0.027503	-1.265518	14.440106	0.000357	-1.397311	14.424935	-0.006029	-1.419901
H	15.160825	0.855103	-1.309071	15.082716	0.885131	-1.450086	15.065905	0.879228	-1.482776
H	15.160717	-0.911140	-1.279888	15.087367	-0.881497	-1.441391	15.073531	-0.887154	-1.457356
H	13.896671	-0.042439	-2.172980	13.790206	-0.005708	-2.280345	13.772478	-0.021541	-2.300904
C	4.875213	-0.005486	0.000021	4.875681	-0.018604	0.074383	4.863659	-0.023875	0.090267
C	4.146518	1.022601	0.622232	4.151445	1.034427	0.661993	4.142516	1.023369	0.690182
C	4.146268	-1.033408	-0.622170	4.147638	-1.074338	-0.503595	4.135858	-1.075343	-0.494015
C	2.758680	1.027395	0.624207	2.765501	1.034368	0.675057	2.756121	1.022351	0.708330
C	2.758428	-1.037890	-0.624101	2.761688	-1.077379	-0.502103	2.749430	-1.079958	-0.486610
C	2.035509	-0.005167	0.000066	2.042023	-0.021716	0.089172	2.033053	-0.029585	0.116259
C	0.607224	-0.005015	0.000102	0.616409	-0.021311	0.091447	0.606394	-0.029955	0.123039
H	4.673302	-1.835194	-1.134785	4.669977	-1.895008	-0.989362	4.658134	-1.890935	-0.988376
H	2.220336	-1.842702	-1.120445	2.221273	-1.898267	-0.967649	2.207637	-1.897409	-0.956545
H	4.673749	1.824266	1.134834	4.677001	1.856569	1.141793	4.670487	1.841429	1.174338
H	2.220784	1.832326	1.120572	2.228178	1.854312	1.145825	2.219763	1.837959	1.187619
H	-2.220710	-1.842303	-1.120404	-2.203676	-1.881283	-0.999981	-2.210471	-1.896243	-0.955428
H	-4.673675	-1.834315	-1.134728	-4.656269	-1.868659	-1.034503	-4.660995	-1.888262	-0.986372
H	-2.220426	1.832786	1.120514	-2.213741	1.843098	1.160570	-2.219524	1.839551	1.188004
H	-4.673392	1.825207	1.134792	-4.666660	1.839982	1.155676	-4.670268	1.844530	1.175600

8.2.3 Orbital correlation diagrams

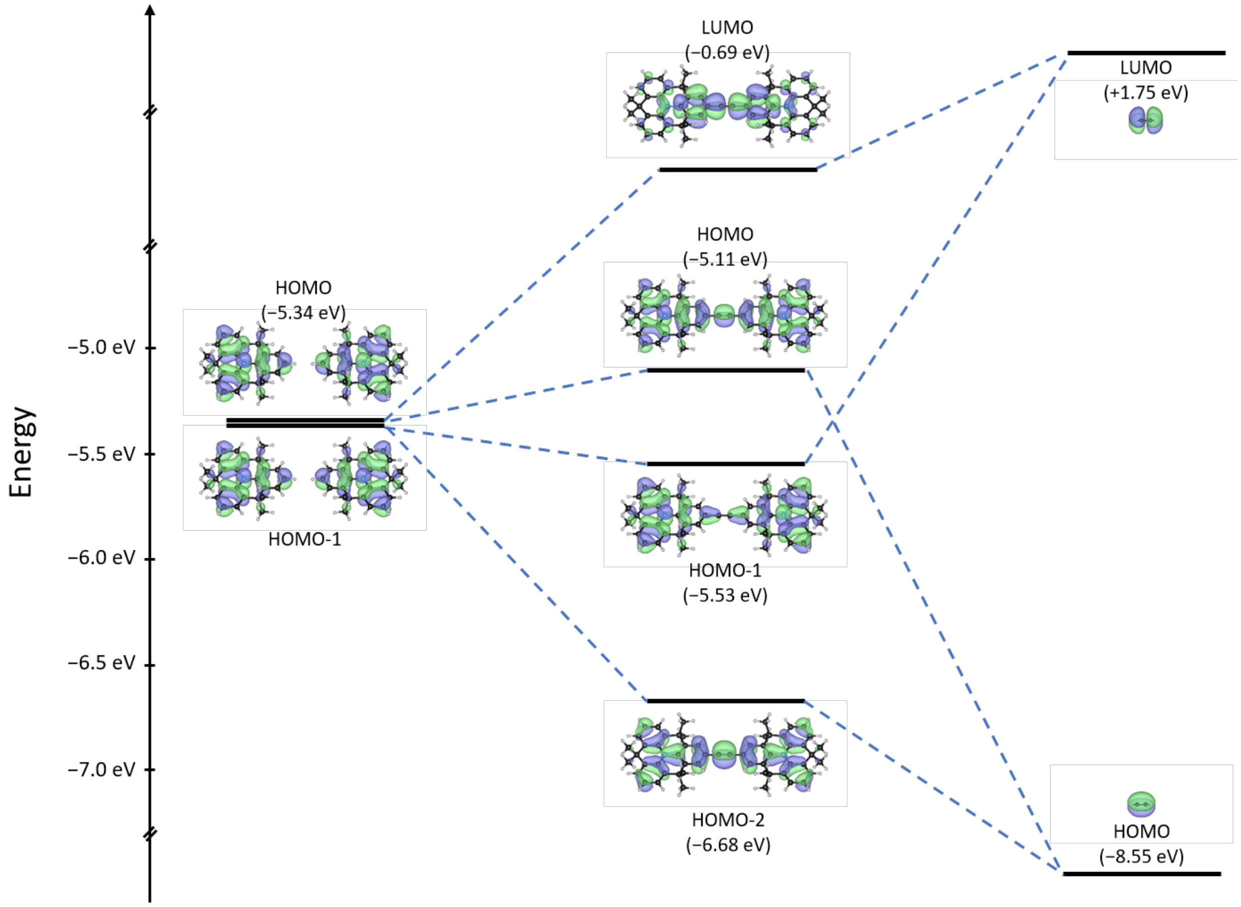

Figure S45: Simplified orbital interaction diagram for the frontier orbitals of DTA. Orbitals were calculated on the TD-BLYP35/def2SVP level in the gas phase (isosurface: ±0.02 a.u.).

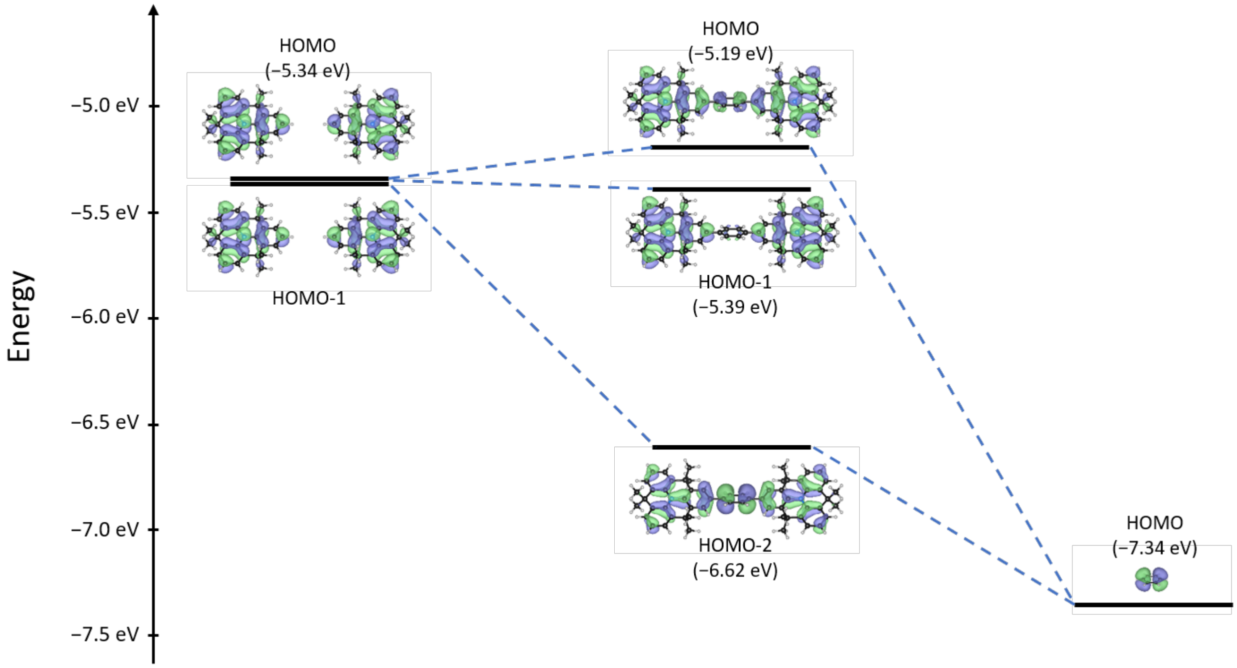

Figure S46: Simplified orbital interaction diagram for the frontier orbitals of DTB. Orbitals were calculated on the TD-BLYP35/def2SVP level in the gas phase (isosurface: ±0.02 a.u.).

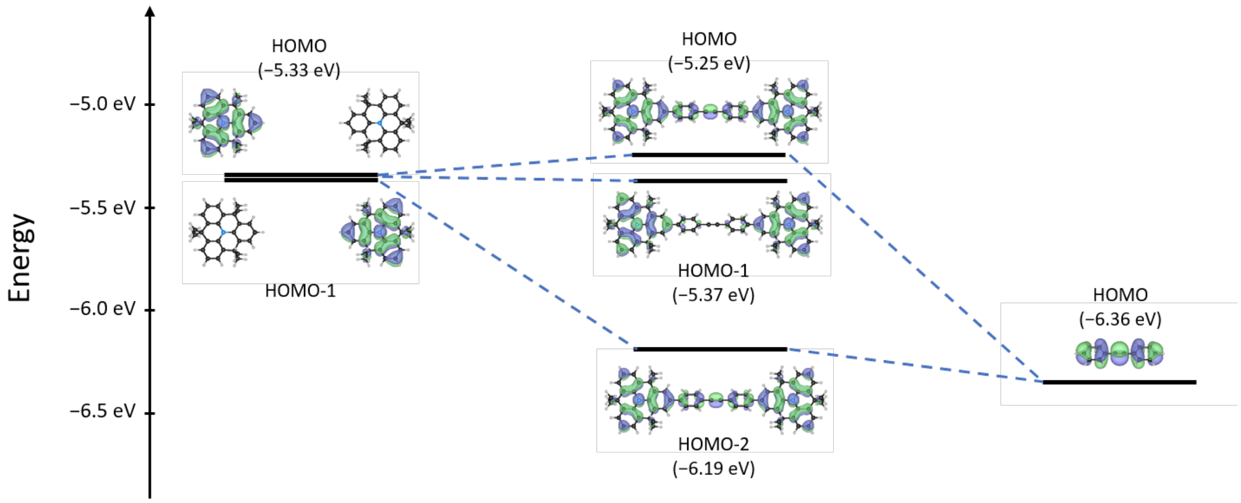

Figure S47: Simplified orbital interaction diagram for the frontier orbitals of DTT. Orbitals were calculated on the TD-BLYP35/def2SVP level in the gas phase (isosurface: ±0.02 a.u.).

8.2.4 Electrostatic potential maps

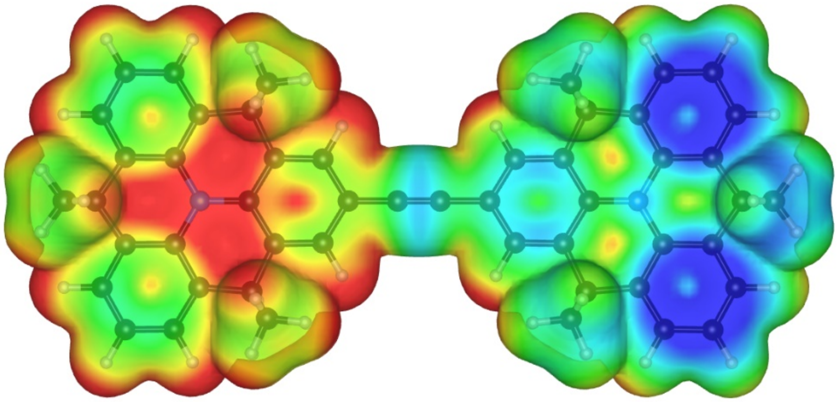

Figure S48: Electrostatic potential (10-70% of max value; blue: relatively low; red: relatively large) of $DTA^{•+}$ mapped on the electron density isosurface (0.005 a.u.). Calculation performed with BLYP35 in CH_2Cl_2.

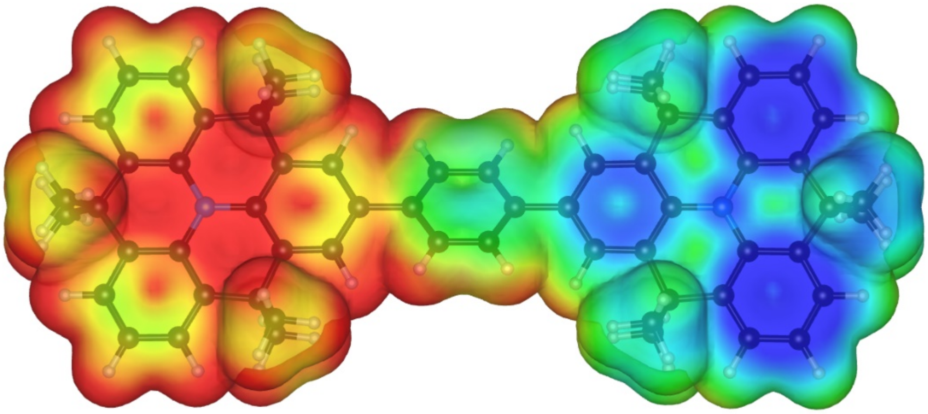

Figure S49: Electrostatic potential (10-70% of max value; blue: relatively low; red: relatively large) of $DTB^{•+}$ mapped on the electron density isosurface (0.005 a.u.). Calculation performed with BLYP35 in CH_2Cl_2.

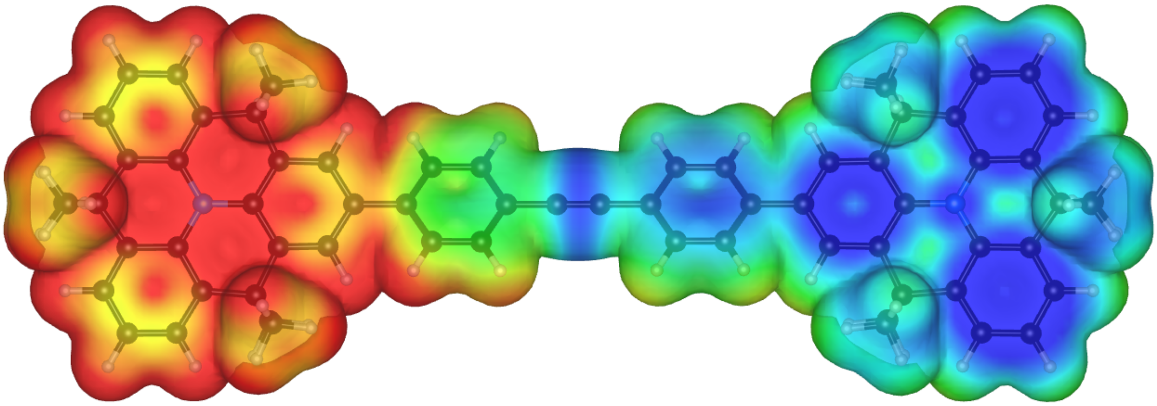

Figure S50: Electrostatic potential (10-70% of max value; blue: relatively low; red: relatively large) of DTT$^{•+}$ mapped on the electron density isosurface (0.005 a.u.). Calculation performed with BLYP35 in CH_2Cl_2.

8.3 Time-dependent calculations

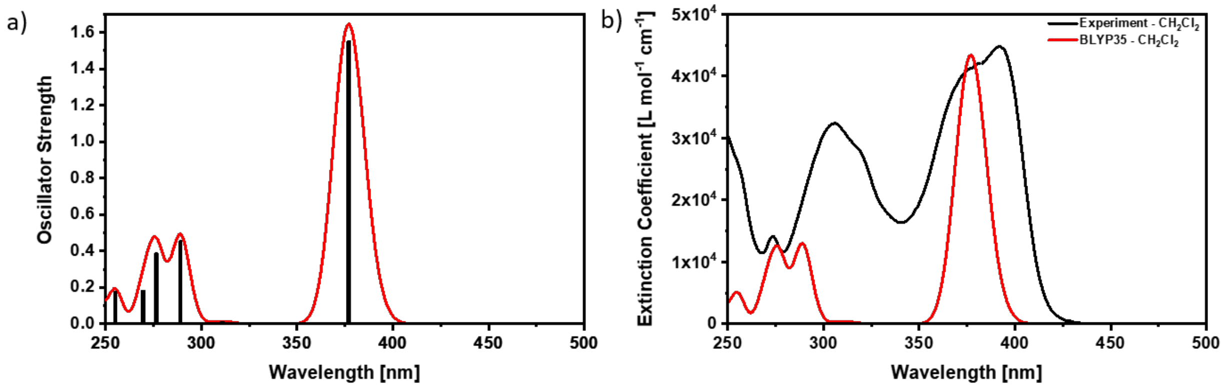

Figure S51: Calculated absorption spectrum (absorption lines were superimposed with Gaussian functions of 0.2 eV width) of DTA in CH_2Cl_2 and b) comparison with the experimental spectrum in CH_2Cl_2.

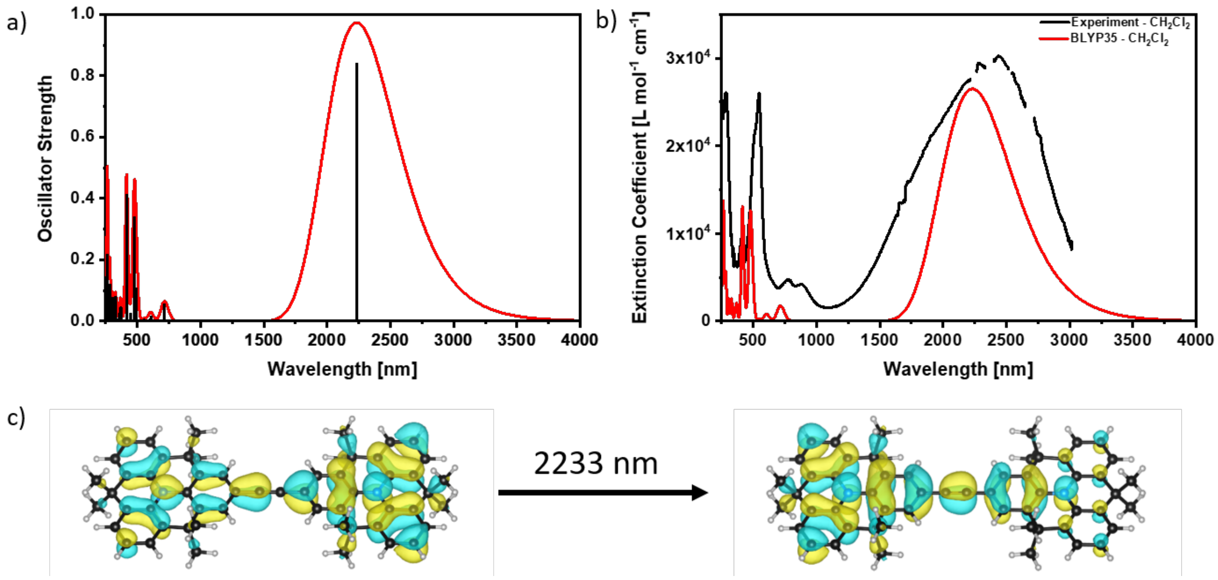

Figure S52: Calculated absorption spectrum (absorption lines were superimposed with Gaussian functions of 0.2 eV width) of $DTA^{\bullet +}$ in CH_2Cl_2 and b) comparison with the experimental spectrum in CH_2Cl_2. c) Natural transition orbitals of the lowest energetic transition.

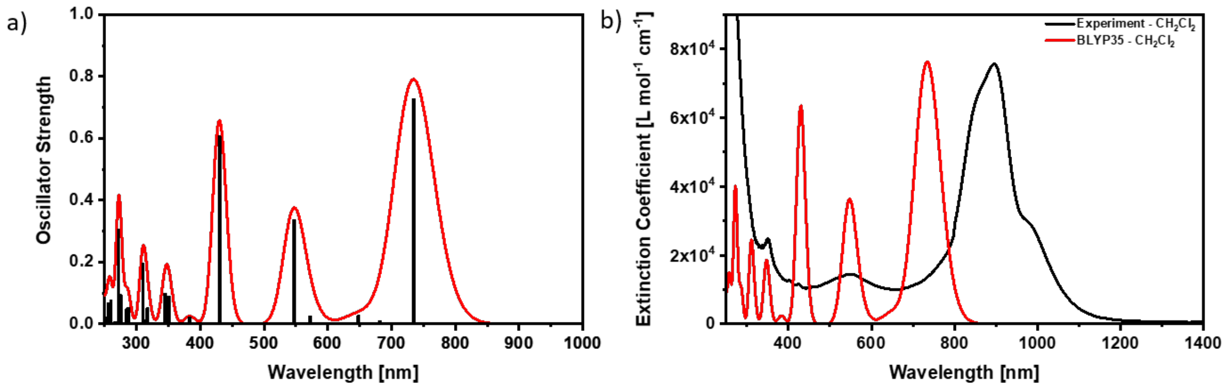

Figure S53: Calculated absorption spectrum (absorption lines were superimposed with Gaussian functions of 0.2 eV width) of DTA^{2+} in CH_2Cl_2 and b) comparison with the experimental spectrum in CH_2Cl_2.

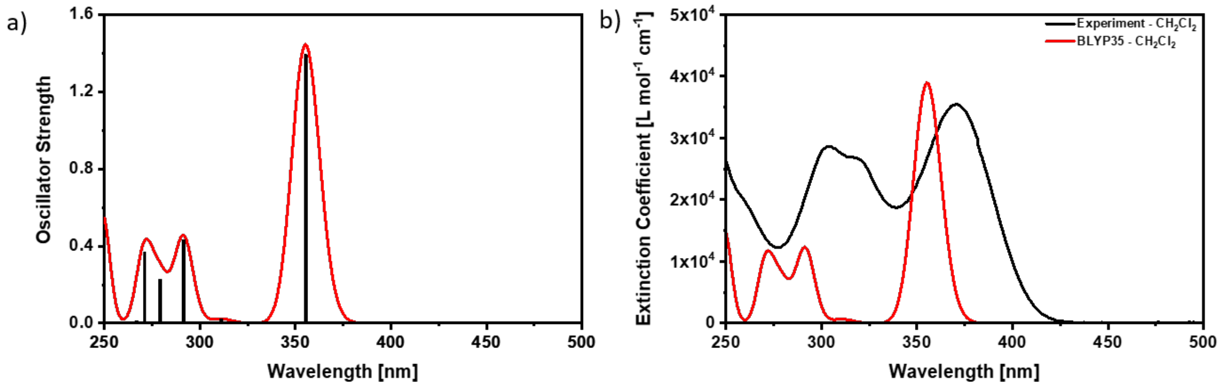

Figure S54: Calculated absorption spectrum (absorption lines were superimposed with Gaussian functions of 0.2 eV width) of DTB in CH_2Cl_2 and b) comparison with the experimental spectrum in CH_2Cl_2.

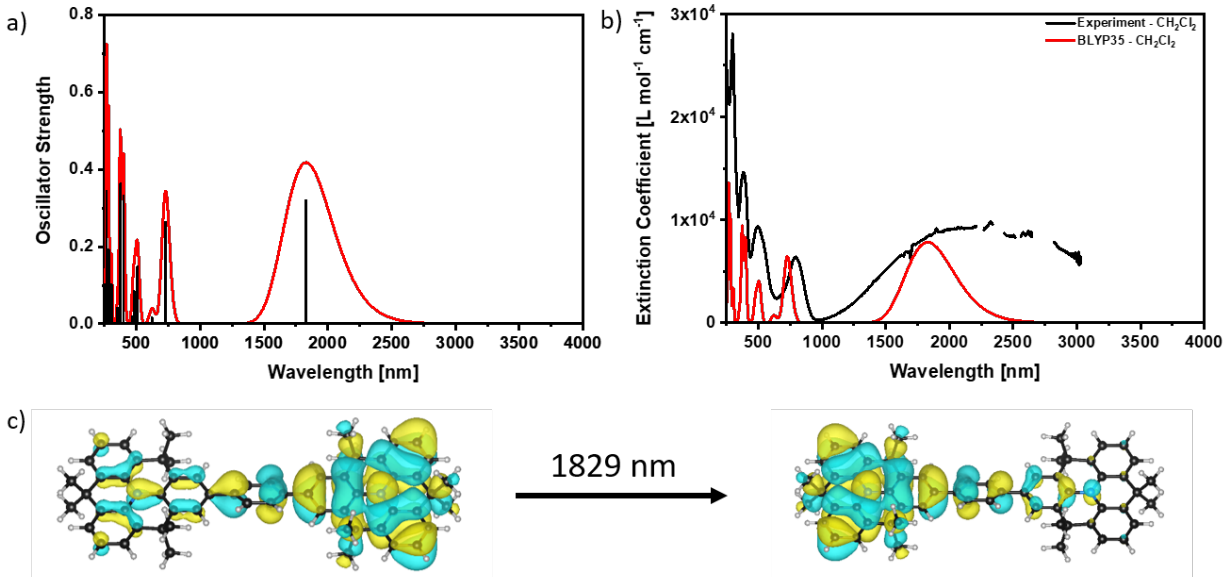

Figure S55: Calculated absorption spectrum (absorption lines were superimposed with Gaussian functions of 0.2 eV width) of DTB$^{\bullet+}$ in CH_2Cl_2 and b) comparison with the experimental spectrum in CH_2Cl_2. c) Natural transition orbitals of the lowest energetic transition.

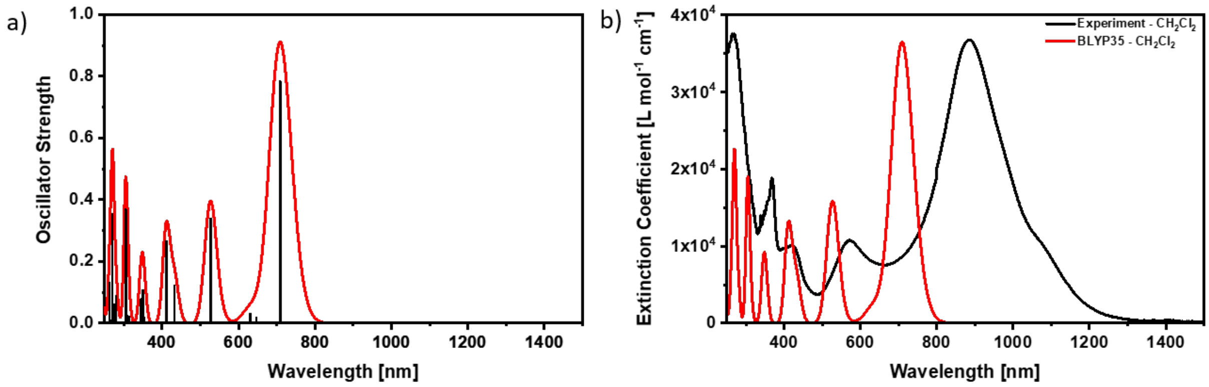

Figure 56: Calculated absorption spectrum (absorption lines were superimposed with Gaussian functions of 0.2 eV width) of DTB^{2+} in CH_2Cl_2 and b) comparison with the experimental spectrum in CH_2Cl_2.

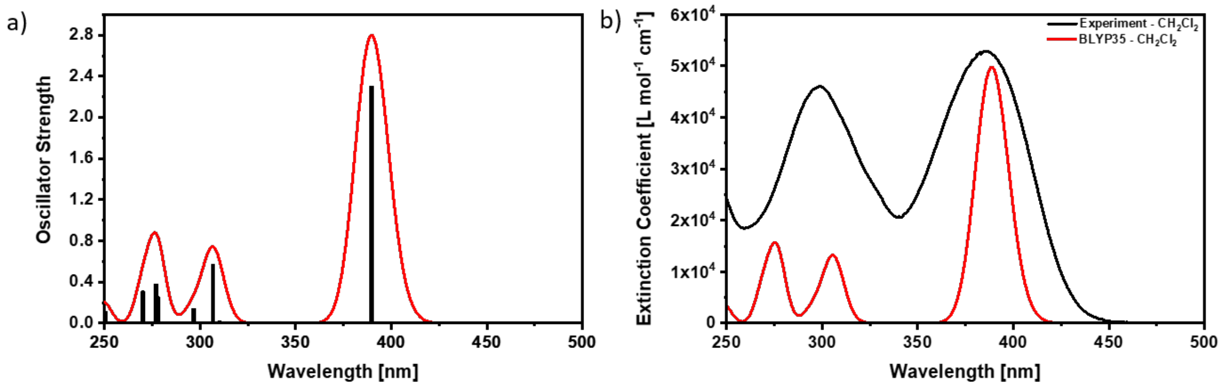

Figure S57: Calculated absorption spectrum (absorption lines were superimposed with Gaussian functions of 0.2 eV width) of DTT in CH_2Cl_2 and b) comparison with the experimental spectrum in CH_2Cl_2.

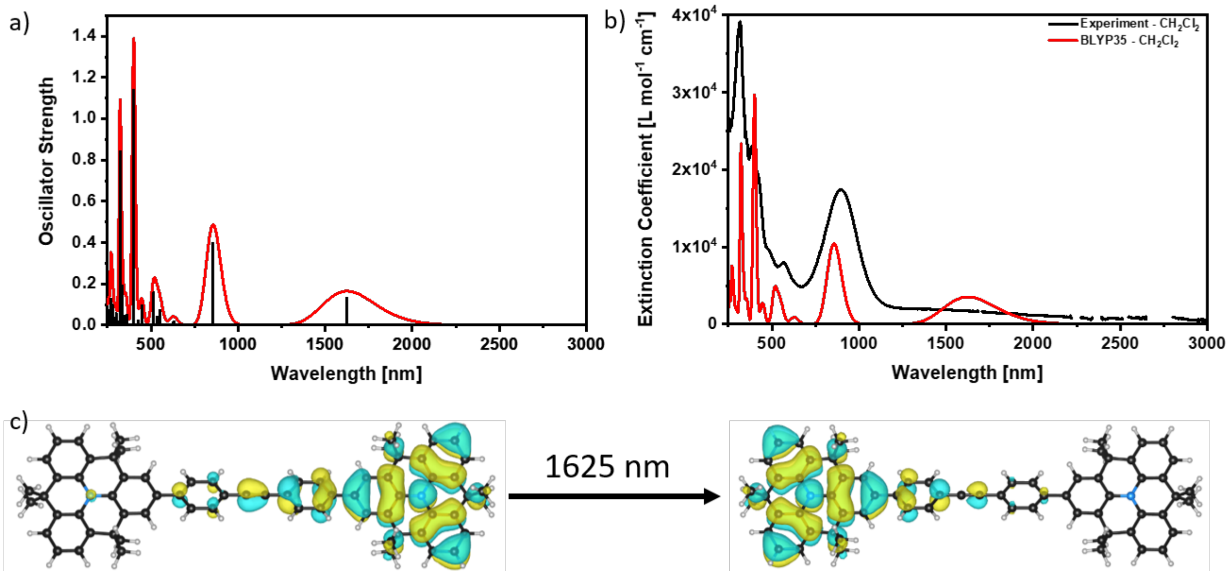

Figure S58: a) Calculated absorption spectrum (absorption lines were superimposed with Gaussian functions of 0.2 eV width) of DTT$^{•+}$ in CH_2Cl_2 and b) comparison with the experimental spectrum in CH_2Cl_2. c) Natural transition orbitals of the lowest energetic transition.

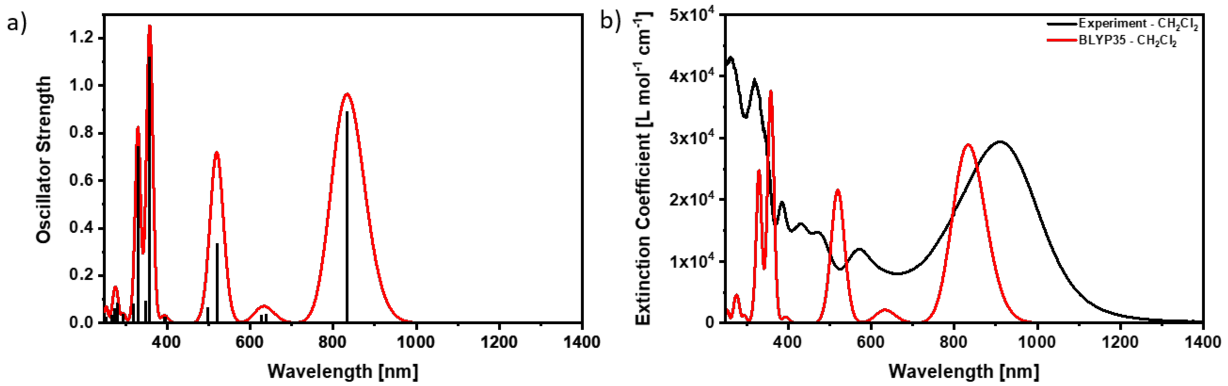

Figure S59: Calculated absorption spectrum (absorption lines were superimposed with Gaussian functions of 0.2 eV width) of DTT^{2+} in CH_2Cl_2 and b) comparison with the experimental spectrum in CH_2Cl_2.

9 EPR-Spectroscopy

9.1 Overview

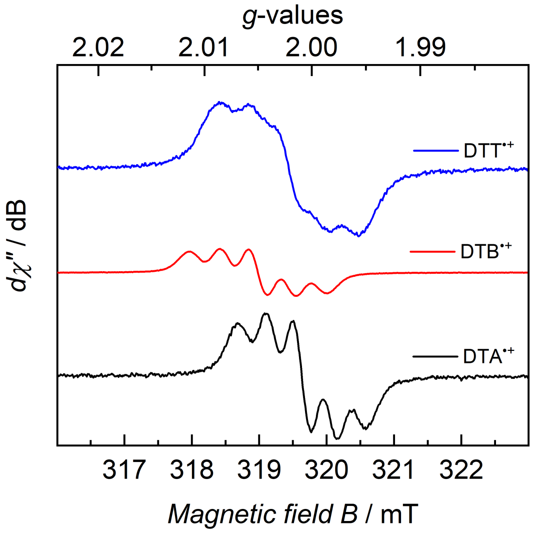

Figure S60: CW X-band EPR spectra of DTA•+ (black trace), DTB•+ (red trace), and DTT•+ (blue trace) recorded as 1 mM solutions in DCM at 293 K. Experimental conditions: microwave frequency ν = 8.959 GHz, modulation width = 0.1; 0.01 mT, microwave power = 1.00 mW, modulation frequency = 100 kHz, time constant = 0.1 s.

Table S9: Characteristic parameters obtained from simulations of the EPR spectra of the radical cations in CH_2Cl_2 at three temperatures

	**DTA**$^{•+}$			**DTB**$^{•+}$			**DTT**$^{•+}$		
	95 K	250K	293K	95K	260K	290K	95K	250K	293K
g-value	2.0016	2.0015	2.0015	2.0043	2.0044	2.0044	2.0015	2.0015	2.0015
Linewidth [10^{-4} cm^{-1} / GHz]	0.720	0.382	0.385	0.774	0.390	0.400	0.290	0.500	0.550
A-tensor [10^{-4} cm^{-1}]	-	3.74	3.73	-	4.00	3.99	-	4.30	4.10

9.2 DTA$^{\bullet+}$

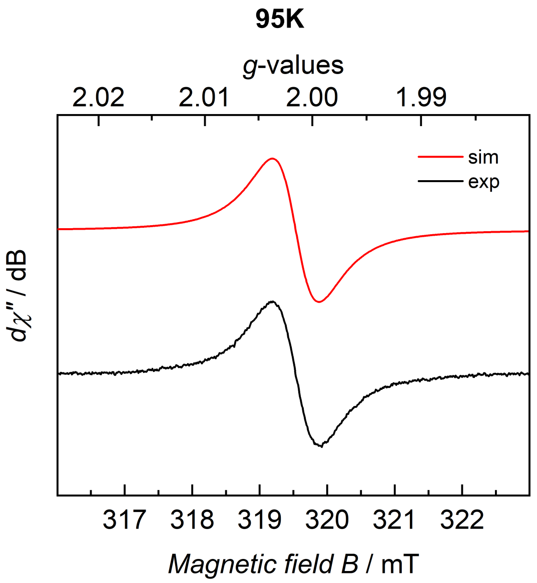

Figure S61: CW X-band EPR spectrum of **DTA$^{\bullet+}$**, recorded as a 1 mM solution in DCM at 95 K (black trace) and its simulation (red trace). Experimental conditions: microwave frequency v = 8.959 GHz, modulation width = 0.01 mT, microwave power = 1.00 mW, modulation frequency = 100 kHz, time constant = 0.1 s. Simulation parameters: effective spin S = ½, effective g-values g_{iso} = 2.0016, linewidths $W_{FWHM,iso}$ = 0.720 × 10^{-4} cm^{-1} / GHz, Voigt ratios (Lorentz = 0, Gauss = 1) V = 0.00.

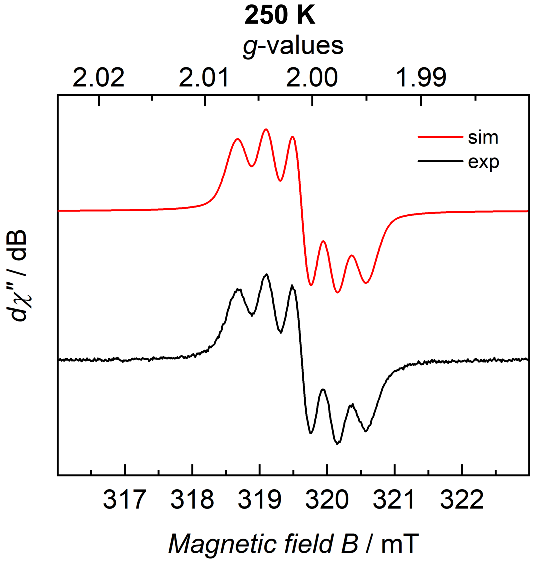

Figure S62: CW X-band EPR spectrum of DTA$^{\bullet+}$, recorded as a 1 mM solution in DCM at 250 K (black trace) and its simulation (red trace). Experimental conditions: microwave frequency v = 8.959 GHz, modulation width = 0.01 mT, microwave power = 1.00 mW, modulation frequency = 100 kHz, time constant = 0.1 s. Simulation parameters: effective spin S = ½, effective g-values g_{iso} = 2.0015, linewidths $W_{FWHM,iso}$ = 0.382 × 10^{-4} cm^{-1} / GHz, Voigt ratios (Lorentz = 0, Gauss = 1) V = 0.51. Super-Hyperfine coupling to two equivalent ^{14}N (I = 1, 99.6% nat. abundance) nuclei was determined as A_{iso} = 3.74 × 10^{-4} cm^{-1}.

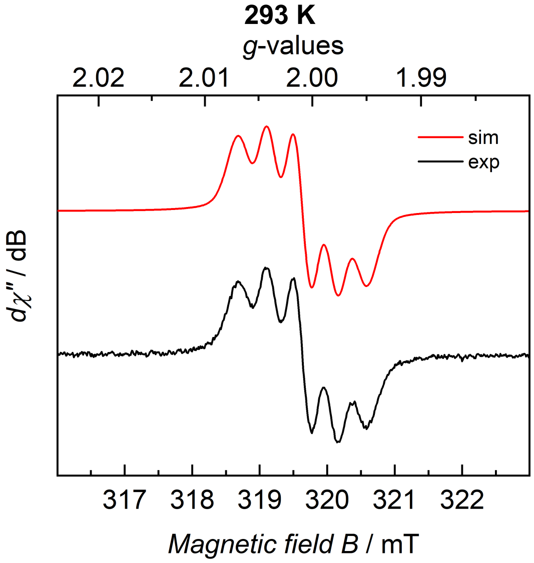

Figure S63: CW X-band EPR spectrum of $DTA^{\bullet+}$, recorded as a 1 mM solution in DCM at 293 K (black trace) and its simulation (red trace). Experimental conditions: microwave frequency v = 8.959 GHz, modulation width = 0.01 mT, microwave power = 1.00 mW, modulation frequency = 100 kHz, time constant = 0.1 s. Simulation parameters: effective spin S = ½, effective g-values g_{iso} = 2.0015, linewidths $W_{FWHM,iso}$ = 0.385 × 10^{-4} cm^{-1} / GHz, Voigt ratios (Lorentz = 0, Gauss = 1) V = 0.55. Super-Hyperfine coupling to two equivalent ^{14}N (I = 1, 99.6% nat. abundance) nuclei was determined as A_{iso} = 3.73 × 10^{-4} cm^{-1}.

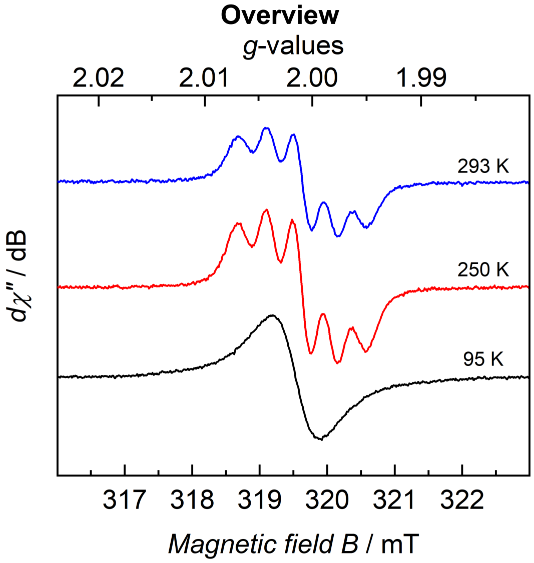

Figure S64: CW X-band EPR spectra of DTA$^{•+}$, recorded as 1 mM solutions in DCM at 95 K (black trace), 250 K (red trace), and 293 K (blue trace). Experimental conditions: microwave frequency v = 8.959 GHz, modulation width = 0.01 mT, microwave power = 1.00 mW, modulation frequency = 100 kHz, time constant = 0.1 s.

9.3 DTB$^{•+}$

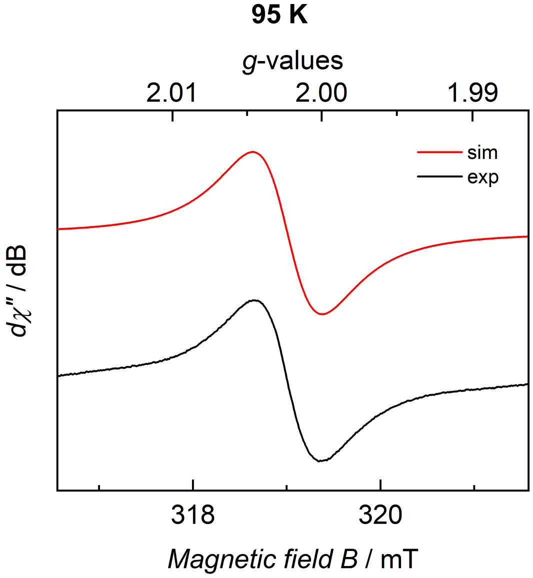

Figure S65: CW X-band EPR spectrum of DTB$^{•+}$, recorded as a 1 mM solution in DCM at 95 K (black trace) and its simulation (red trace). Experimental conditions: microwave frequency v = 8.959 GHz, modulation width = 0.1 mT, microwave power = 1.00 mW, modulation frequency = 100 kHz, time constant = 0.1 s. Simulation parameters: effective spin S = ½, effective g-values g_{iso} = 2.0043, linewidths $W_{FWHM,iso}$ = 0.774 × 10^{-4} cm^{-1} / GHz, Voigt ratios (Lorentz = 0, Gauss = 1) V = 0.00.

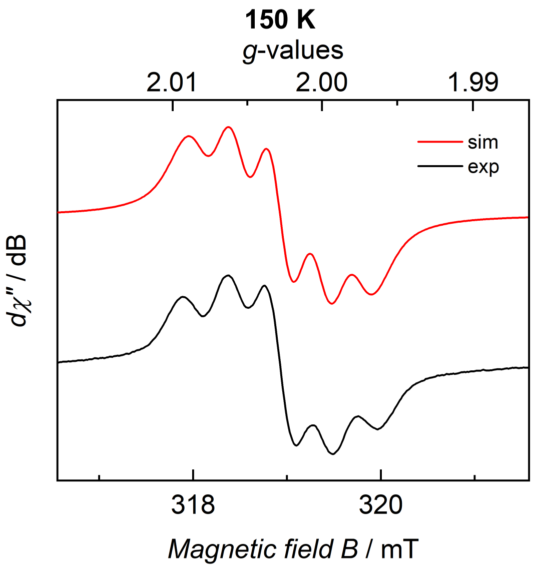

Figure S66: CW X-band EPR spectrum of DTB$^{•+}$, recorded as a 1 mM solution in DCM at 150 K (black trace) and its simulation (red trace). Experimental conditions: microwave frequency v = 8.959 GHz, modulation width = 0.1 mT, microwave power = 1.00 mW, modulation frequency = 100 kHz, time constant = 0.1 s. Simulation parameters: effective spin S = ½, effective g-values g_{iso} = 2.0043, linewidths $W_{FWHM,iso}$ = 0.398 × 10^{-4} cm^{-1} / GHz, Voigt ratios (Lorentz = 0, Gauss = 1) V = 0.15. Super-Hyperfine coupling to two equivalent ^{14}N (I = 1, 99.6% nat. abundance) nuclei was determined as A_{iso} = 3.90 × 10^{-4} cm^{-1}.

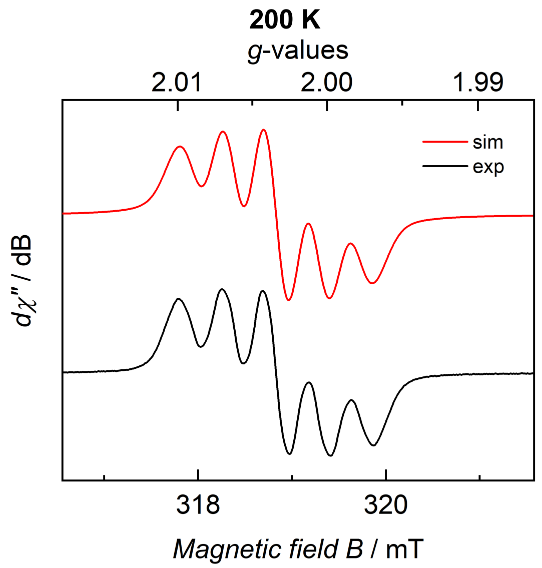

Figure S67: CW X-band EPR spectrum of $DTB^{\bullet+}$, recorded as a 1 mM solution in DCM at 200 K (black trace) and its simulation (red trace). Experimental conditions: microwave frequency v = 8.959 GHz, modulation width = 0.1 mT, microwave power = 1.00 mW, modulation frequency = 100 kHz, time constant = 0.1 s. Simulation parameters: effective spin S = ½, effective g-values g_{iso} = 2.0044, linewidths $W_{FWHM,iso}$ = 0.377 × 10^{-4} cm^{-1} / GHz, Voigt ratios (Lorentz = 0, Gauss = 1) V = 0.45. Super-Hyperfine coupling to two equivalent ^{14}N (I = 1, 99.6% nat. abundance) nuclei was determined as A_{iso} = 4.05 × 10^{-4} cm^{-1}.

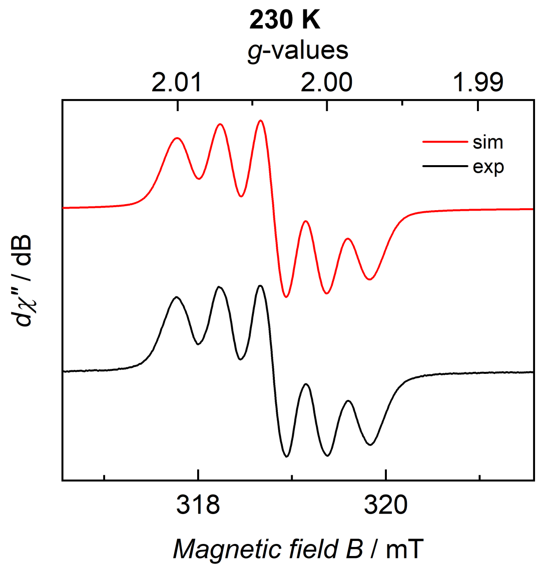

Figure S68: CW X-band EPR spectrum of $DTB^{•+}$, recorded as a 1 mM solution in DCM at 230 K (black trace) and its simulation (red trace). Experimental conditions: microwave frequency v = 8.959 GHz, modulation width = 0.1 mT, microwave power = 1.00 mW, modulation frequency = 100 kHz, time constant = 0.1 s. Simulation parameters: effective spin S = ½, effective g-values g_{iso} = 2.0044, linewidths $W_{FWHM,iso}$ = 0.384 × 10^{-4} cm^{-1} / GHz, Voigt ratios (Lorentz = 0, Gauss = 1) V = 0.63. Super-Hyperfine coupling to two equivalent ^{14}N (I = 1, 99.6% nat. abundance) nuclei was determined as A_{iso} = 4.03 × 10^{-4} cm^{-1}.

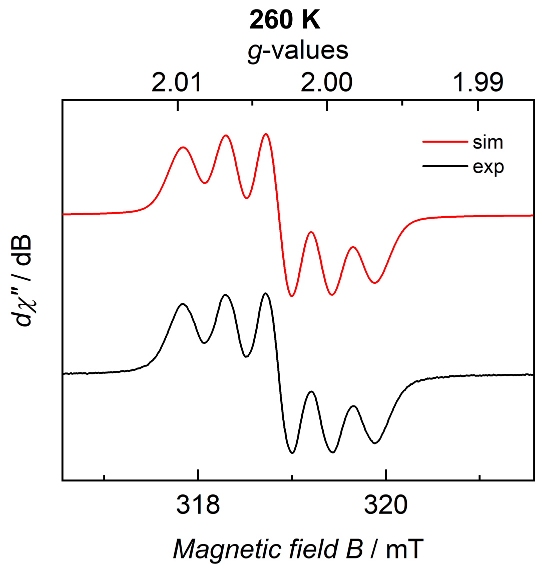

Figure S69: CW X-band EPR spectrum of $DTB^{•+}$, recorded as a 1 mM solution in DCM at 260 K (black trace) and its simulation (red trace). Experimental conditions: microwave frequency v = 8.959 GHz, modulation width = 0.1 mT, microwave power = 1.00 mW, modulation frequency = 100 kHz, time constant = 0.1 s. Simulation parameters: effective spin S = ½, effective g-values g_{iso} = 2.0044, linewidths $W_{FWHM,iso}$ = 0.390 × 10^{-4} cm^{-1} / GHz, Voigt ratios (Lorentz = 0, Gauss = 1) V = 0.66. Super-Hyperfine coupling to two equivalent ^{14}N (I = 1, 99.6% nat. abundance) nuclei was determined as A_{iso} = 4.00 × 10^{-4} cm^{-1}.

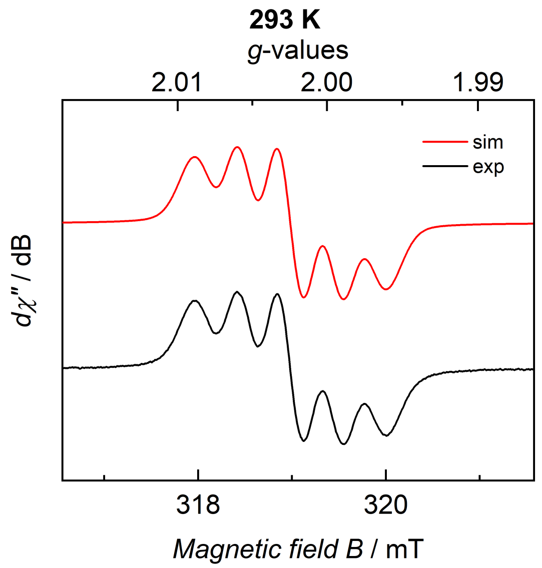

Figure S70: CW X-band EPR spectrum of $DTB^{\bullet+}$, recorded as a 1 mM solution in DCM at 293 K (black trace) and its simulation (red trace). Experimental conditions: microwave frequency v = 8.959 GHz, modulation width = 0.1 mT, microwave power = 1.00 mW, modulation frequency = 100 kHz, time constant = 0.1 s. Simulation parameters: effective spin S = ½, effective g-values g_{iso} = 2.0044, linewidths $W_{FWHM,iso}$ = 0.400 × 10^{-4} cm^{-1} / GHz, Voigt ratios (Lorentz = 0, Gauss = 1) V = 0.66. Super-Hyperfine coupling to two equivalent ^{14}N (I = 1, 99.6% nat. abundance) nuclei was determined as A_{iso} = 3.99 × 10^{-4} cm^{-1}.

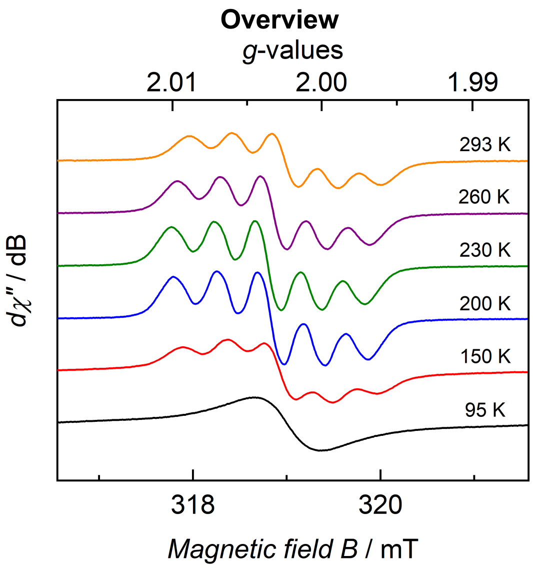

Figure S71: CW X-band EPR spectra of DTB$^{•+}$, recorded as 1 mM solutions in DCM at 95 K (black trace), 150 K (red trace), 200 K (blue trace), 230 K (green trace), 260 K (purple trace), and 293 K (orange trace). Experimental conditions: microwave frequency v = 8.959 GHz, modulation width = 0.1 mT, microwave power = 1.00 mW, modulation frequency = 100 kHz, time constant = 0.1 s.

9.4 DTT$^{•+}$

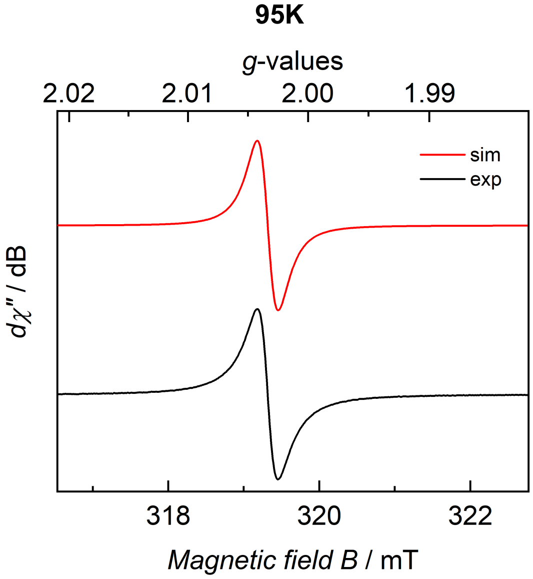

Figure S72: CW X-band EPR spectrum of DTT$^{•+}$, recorded as a 1 mM solution in DCM at 95 K (black trace) and its simulation (red trace). Experimental conditions: microwave frequency v = 8.959 GHz, modulation width = 0.01 mT, microwave power = 1.00 mW, modulation frequency = 100 kHz, time constant = 0.1 s. Simulation parameters: effective spin S = ½, effective g-values g_{iso} = 2.0015, linewidths $W_{FWHM,iso}$ = 0.290 × 10^{-4} cm^{-1} / GHz, Voigt ratios (Lorentz = 0, Gauss = 1) V = 0.00.

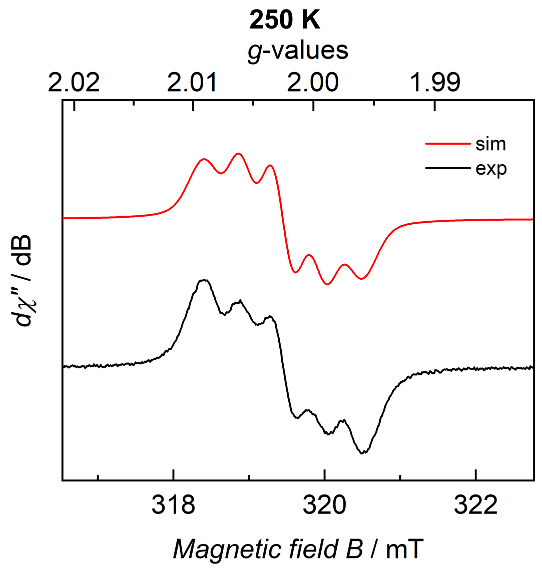

Figure S73: CW X-band EPR spectrum of $DTT^{\bullet+}$, recorded as a 1 mM solution in DCM at 250 K (black trace) and its simulation (red trace). Experimental conditions: microwave frequency v = 8.959 GHz, modulation width = 0.01 mT, microwave power = 1.00 mW, modulation frequency = 100 kHz, time constant = 0.1 s. Simulation parameters: effective spin S = ½, effective g-values g_{iso} = 2.0015, linewidths $W_{FWHM,iso}$ = 0.500 × 10^{-4} cm^{-1} / GHz, Voigt ratios (Lorentz = 0, Gauss = 1) V = 0.45. Super-Hyperfine coupling to two equivalent ^{14}N (I = 1, 99.6% nat. abundance) nuclei was determined as A_{iso} = 4.30 × 10^{-4} cm^{-1}.

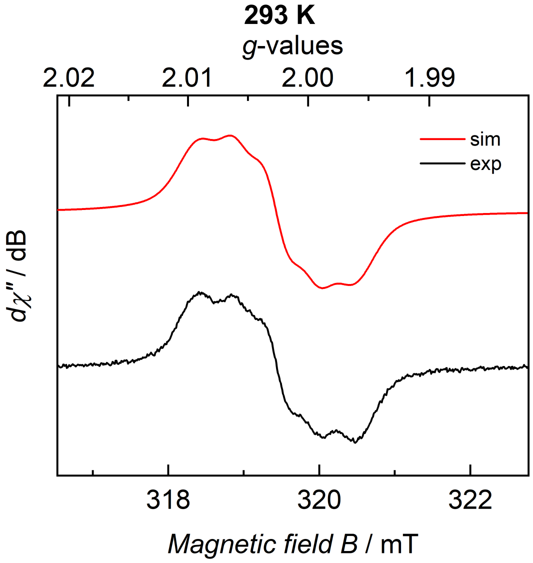

Figure S74: CW X-band EPR spectrum of DTT$^{•+}$, recorded as a 1 mM solution in DCM at 293 K (black trace) and its simulation (red trace). Experimental conditions: microwave frequency v = 8.959 GHz, modulation width = 0.01 mT, microwave power = 1.00 mW, modulation frequency = 100 kHz, time constant = 0.1 s. Simulation parameters: effective spin S = ½, effective g-values g_{iso} = 2.0015, linewidths $W_{FWHM,iso}$ = 0.550 × 10^{-4} cm^{-1} / GHz, Voigt ratios (Lorentz = 0, Gauss = 1) V = 0.30. Super-Hyperfine coupling to two equivalent ^{14}N (I = 1, 99.6% nat. abundance) nuclei was determined as A_{iso} = 4.10 × 10^{-4} cm^{-1}.

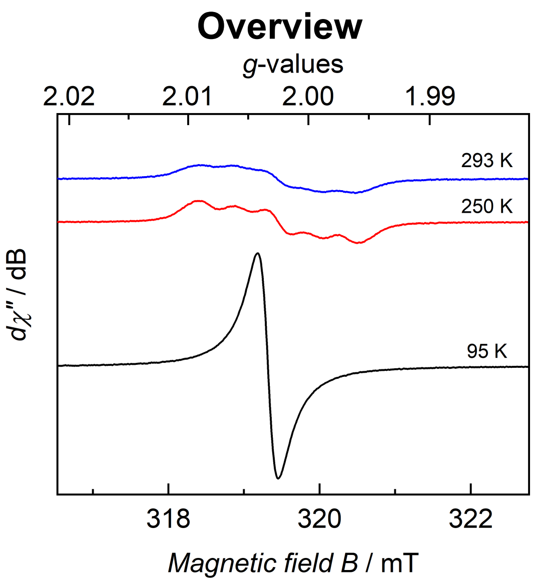

Figure S75: CW X-band EPR spectra of DTT$^{\bullet+}$, recorded as 1 mM solutions in DCM at 95 K (black trace), 250 K (red trace), and 293 K (blue trace). Experimental conditions: microwave frequency v = 8.959 GHz, modulation width = 0.01 mT, microwave power = 1.00 mW, modulation frequency = 100 kHz, time constant = 0.1 s.

References

[1] M. Holzapfel, C. Lambert, D. Stalke, *J. Chem. Soc., Perkin Trans.* **2002**, *2*, 1553–1561.
[2] H. E. Gottlieb, V. Kotlyar, A. Nudelman, *J. Org. Chem.* **1997**, *62*, 7512–7515.
[3] B. J. Gaffney, H. J. Silverstone, in *EMR Paramagn. Mol. Biol. Magn. Reson.*, Springer, Boston, USA, **1993**, pp. 1–57.
[4] M. Samoc, Z. Fang, L. Cai, A. Samoc, T.-L. Teo, Y.-H. Lai, *Org. Lett.* **2008**, *11*, 1–4.
[5] F. Schlütter, F. Rossel, M. Kivala, V. Enkelmann, J. P. Gisselbrecht, P. Ruffieux, R. Fasel, K. Müllen, *J. Am. Chem. Soc.* **2013**, *135*, 4550–4557.
[6] L. Krause, R. Herbst-Irmer, G. M. Sheldrick, D. Stalke, *J. Appl. Crystallogr.* **2015**, *48*, 3–10.
[7] G. M. Sheldrick, *Acta Crystallogr. Sect. A Found. Crystallogr.* **2015**, *71*, 3–8.
[8] G. M. Sheldrick, *Acta Crystallogr. Sect. C Struct. Chem.* **2015**, *71*, 3–8.
[9] M. Mardelli, J. Olmsted, *J. Photochem.* **1977**, *7*, 277–285.
[10] G. A. Reynolds, K. H. Drexhage, *Opt. Commun.* **1975**, *13*, 222–225.
[11] M. J. Frisch, G. W. Trucks, H. B. Schlegel, G. E. Scuseria, M. A. Robb, J. R. Cheeseman, G. Scalmani, V. Barone, G. A. Petersson, H. Nakatsuji, X. Li, M. Caricato, A. V. Marenich, J. Bloino, B. G. Janesko, R. Gomperts, B. Mennucci, H. B. Hratchian, J. V. Ortiz, A. F. Izmaylov, J. L. Sonnenberg, D. Williams-Young, F. Ding, F. Lipparini, F. Egidi, J. Goings, B. Peng, A. Petrone, T. Henderson, D. Ranasinghe, V. G. Zakrzewski, J. Gao, N. Rega, G. Zheng, W. Liang, M. Hada, M. Ehara, K. Toyota, R. Fukuda, J. Hasegawa, M. Ishida, T. Nakajima, Y. Honda, O. Kitao, H. Nakai, T. Vreven, K. Throssell, J. A. Montgomery, Jr., J. E. Peratta, F. Ogliaro, M. J. Bearpark, J. J. Heyd, E. N. Brothers, K. N. Kudin, V. N. Staroverov, T. A. Keith, R. Kobayashi, J. Normand, K. Raghavachari, A. P. Rendell, J. C. Burant, S. S. Iyengar, J. Tomasi, M. Cossi, J. M. Millam, M. Klene, C. Adamo, R. Cammi, J. W. Ochterski, R. L. Martin, K. Morokuma, O. Farkas, J. B. Foresman, D. J. Fox, *Gaussian 16*, Gaussian, Inc., Wallingford CT, **2016**.
[12] M. Renz, K. Theilacker, C. Lambert, M. Kaupp, *J. Am. Chem. Soc.* **2009**, *131*, 16292–16302.
[13] M. Kaupp, M. Renz, M. Parthey, M. Stolte, F. Würthner, C. Lambert, *Phys. Chem. Chem. Phys.* **2011**, *13*, 16973–16986.
[14] A. D. Becke, *Phys. Rev. A* **1988**, *38*, 3098–3100.
[15] C. Lee, W. Yang, R. G. Parr, *Phys. Rev. B* **1988**, *37*, 785–789.
[16] F. Weigend, R. Ahlrichs, *Phys. Chem. Chem. Phys.* **2005**, *7*, 3297–3305.
[17] M. Cossi, N. Rega, G. Scalmani, V. Barone, *J. Comput. Chem.* **2003**, *24*, 669–681.
[18] M. Uebe, A. Ito, *Chem. - An Asian J.* **2019**, *14*, 1692–1696.
[19] P. Mayorga Burrezo, W. Zeng, M. Moos, M. Holzapfel, S. Canola, F. Negri, C. Rovira, J. Veciana, H. Phan, J. Wu, C. Lambert, J. Casado, *Angew. Chem. Int. Ed.* **2019**, *58*, 14467–14471; *Angew.Chem.* **2005**, *117*, 5263–5265.
[20] G. Tan, X. Wang, *Acc. Chem. Res.* **2017**, *50*, 1997–2006.
[21] K. Momma, F. Izumi, *J. Appl. Crystallogr.* **2011**, *44*, 1272–1276.
